# Use of Epic Electronic Health Record System for Health Care Research: Scoping Review

**DOI:** 10.2196/51003

**Published:** 2023-12-15

**Authors:** Jawad Chishtie, Natalie Sapiro, Natalie Wiebe, Leora Rabatach, Diane Lorenzetti, Alexander A Leung, Doreen Rabi, Hude Quan, Cathy A Eastwood

**Affiliations:** 1 Center for Health Informatics University of Calgary Calgary, AB Canada; 2 Alberta Health Services Calgary, AB Canada; 3 Bethany Care Society Calgary, AB Canada; 4 Community Health Sciences University of Calgary Calgary, AB Canada; 5 Health Sciences Library University of Calgary Calgary, AB Canada; 6 Department of Medicine University of Calgary Calgary, AB Canada

**Keywords:** electronic health record, EHR, Epic, research, health care, electronic medical record, EMR, health system

## Abstract

**Background:**

Electronic health records (EHRs) enable health data exchange across interconnected systems from varied settings. Epic is among the 5 leading EHR providers and is the most adopted EHR system across the globe. Despite its global reach, there is a gap in the literature detailing how EHR systems such as Epic have been used for health care research.

**Objective:**

The objective of this scoping review is to synthesize the available literature on use cases of the Epic EHR for research in various areas of clinical and health sciences.

**Methods:**

We used established scoping review methods and searched 9 major information repositories, including databases and gray literature sources. To categorize the research data, we developed detailed criteria for 5 major research domains to present the results.

**Results:**

We present a comprehensive picture of the method types in 5 research domains. A total of 4669 articles were screened by 2 independent reviewers at each stage, while 206 articles were abstracted. Most studies were from the United States, with a sharp increase in volume from the year 2015 onwards. Most articles focused on clinical care, health services research and clinical decision support. Among research designs, most studies used longitudinal designs, followed by interventional studies implemented at single sites in adult populations. Important facilitators and barriers to the use of Epic and EHRs in general were identified. Important lessons to the use of Epic and other EHRs for research purposes were also synthesized.

**Conclusions:**

The Epic EHR provides a wide variety of functions that are helpful toward research in several domains, including clinical and population health, quality improvement, and the development of clinical decision support tools. As Epic is reported to be the most globally adopted EHR, researchers can take advantage of its various system features, including pooled data, integration of modules and developing decision support tools. Such research opportunities afforded by the system can contribute to improving quality of care, building health system efficiencies, and conducting population-level studies. Although this review is limited to the Epic EHR system, the larger lessons are generalizable to other EHRs.

## Introduction

### Background

Electronic medical record (EMR) and electronic health record (EHR) systems are being increasingly used for storing, retrieving, and managing patient health records that can be used for data-driven initiatives, such as clinical research, quality improvement (QI), and decision support tools [1]. Although both terms are commonly used interchangeably, *EMR* specifically refers to patient health data accumulated by patient encounters and limited to care delivery settings, enabled by the interoperability of the system by multiple providers [2,3]. These data typically consist of demographics and health notes, including history, past and current problems, immunizations and treatments, and laboratory and radiology reports. In contrast, *EHR* refers to larger, interconnected systems that bring together EMRs from various care sites and settings to enable robust information exchange [2,3]. EHRs are also designed for access by varied audiences, capturing far more than just clinical information and are used to support continuity and delivery of integrated and efficient health care services [2].

EMR and EHR systems are important sources of research data that can be used to improve patient care journeys, transitions between levels of health care, and delivery of services. With developments in computer systems and the use of digital health tools, EHR systems have evolved significantly since their first use in the 1960s [4], with the global market reaching US $33 billion by 2025 [5]. Epic Systems [6] (Epic Systems Corporation) was one of the early innovators in this space and is currently among the 5 leading EHR providers and the most adopted EHR worldwide [7,8]. In early 2023, Epic was independently rated as the best overall EHR suite for the 13th consecutive year and was in use in 89% of acute care hospitals in the United States [9]. Many academic and clinical organizations, such as Johns Hopkins University, have adopted Epic across their care delivery systems [10,11]. Recently, the province of Alberta, Canada, transitioned its varied EHRs to a unified Epic-based clinical information system called Connect Care, sparking great interest in the research community for the potential use of real-time clinical and health data in Canada [12].

### Rationale

Despite the global reach of the Epic EHR, there remains a gap in literature detailing how the system has been used to facilitate clinical and health research. Literature syntheses are available describing issues related to EHR implementation [13], the transition to the Epic EHR [8], and Epic integration for personalized care [14], whereas a plethora of Epic implementation studies are also available [15,16]. Two reviews on the use of EHRs and EMRs for research included a narrative review from 2009, capturing literature from 2000 to 2007 on health outcomes [17], and a more recent review from 2015 examined population health methods and applications [18]. Both reviews, however, are dated, do not focus on a single EHR, do not capture the breadth of the literature on areas of healthcare research, and do not follow methods for systematic literature syntheses.

To the best of our knowledge, a comprehensive literature review on the use of the Epic EHR for health care research is not available. We address this critical gap in the literature. Hence, the objective of this scoping review is to synthesize literature on use cases of the Epic EHR for research in various areas of clinical and health sciences.

## Methods

### Scoping Review Methodology

We selected the scoping review method because it provides the basis for synthesizing and capturing the breadth of the current peer-reviewed and gray literature [19,20]. Scoping reviews enable researchers to comprehensively assess the breadth and depth of a topic and identify the strengths, weaknesses, and gaps in the existing research base. As the focus of our review was to explore the existing research applications of Epic, we determined that a scoping review methodology would be the best approach for achieving this objective [21]. We followed the Joanna Briggs Institute guidance [22] and foundational guidelines from Arksey and O’Malley’s [21] 2005 framework to conduct scoping reviews. Our approach was further supported by the unpacking details suggested by Levac et al [23] and Peters et al [24]. As Levac et al [23] suggested involving stakeholders, the review was conceptualized in consultation with a multidisciplinary team that included experts from health informatics, clinicians, data scientists, and health system managers. A brief review protocol was developed, but not published.

On the basis of the JBI guidelines, we used the population, concepts, and context framework to formulate the research question. Our population included all individuals with any condition of interest. The concepts included all possible research methods that involve the collection, integration, extraction, and analysis of data or tools built to support research using the Epic EHR. The context could be the use of EHRs in any setting. The review steps comprised determining the research question, identifying relevant studies, title, abstract and full-text screening, and data charting. Synthesis and reporting of the results used the PRISMA-ScR (Preferred Reporting Items for Systematic Reviews and Meta-Analyses extension for Scoping Reviews) checklist [25].

### Eligibility Criteria

To develop the eligibility criteria, we conducted an initial search of the literature on OVID MEDLINE and reviewed the first 50 articles that presented the use of EHRs for research. As a first step, reviewers (JC, NS, NW, LR, and DR) independently screened these 50 pilot articles, followed by a meeting to discuss the conflicts and eligibility criteria. Each pilot article was independently reviewed by 2 reviewers at both stages of title, abstract, and full-text screening.

Working with a multidisciplinary team for this initial phase of the search, we defined the parameters of the review and identified the variety of ways Epic has been used for research. First, we learned that there were 2 clear categories of literature. Aligned with our objective, we focused on the use of Epic EHR *for research*, delineating our inquiry from research *on the Epic EHR system*. The latter body of literature includes the implementation, development of modules, enhancements in different versions, and architectural, legal, and security issues related to the Epic EHR. The consensus of our multidisciplinary team was to exclude literature related to *research on the Epic EHR system* as it diverged from our main objective.

Furthermore, our initial searches revealed major research domains within health care that included clinical and health services research (HSR), population health, clinical decision support (CDS) tools, registries, biobanks, and QI initiatives. The team reached a consensus to use these 6 domains to categorize the research literature while allowing others to emerge during the synthesis phase. The purpose of grouping the literature in this manner kept the team’s focus on the methods and use cases of Epic for conducting research. Each domain was determined according to the stated purpose and method of each study. If the research fits in more than one domain, a primary domain is identified. The eligibility criteria are presented in Textbox 1. We also focused on capturing facilitators and barriers to using the Epic EHR for clinical or health research, which could include specific details about the software, ease or difficulty in extracting data, building new tools or fields, and achieving research objectives in general.

**Eligibility criteria for selection of articles.**

**Inclusion criteria**

- Articles presenting use of Epic-based systems for research that include (1) health services research; (2) registries and biobanks; (3) clinical decision support; (4) clinical research; (5) quality improvement; and (6) population health research
- Any other articles presenting research use of Epic
- Articles on clinical decision support tools, with a research component, for example, testing out the effectiveness of algorithms for clinical care delivery, quality improvement, and health service delivery
- Articles in English language

**Exclusion criteria**

- Articles not in English
- Articles on Epic that include implementation, transitioning from another electronic health record to Epic, legal issues, Epic modules, cognitive testing, human computer interaction studies, or safety concerns
- Articles on development of clinical decision support tools, without a research component
- Editorials, commentaries, trial registrations
- Studies without a research question, clear sample selection or results

### Information Sources

**Figure 1:**
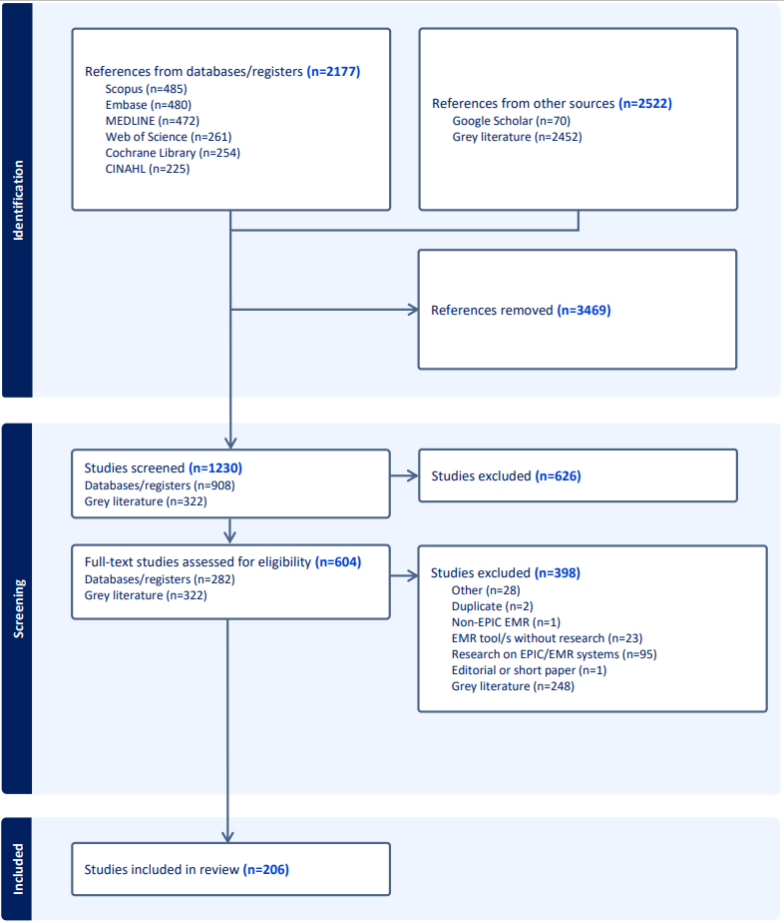
PRISMA (Preferred Reporting Items for Systematic Reviews and Meta-Analyses) flowchart. EMR: electronic medical record.

The search strategy for peer-reviewed literature was formulated and implemented by an information specialist (DL) on the team. We included 3 major health databases (MEDLINE, Embase, and CINAHL) and 2 interdisciplinary databases (Scopus and Web of Science). We did not place a time limit on the search, searching for all sources until April 25, 2023. To identify previously published reviews, we searched the Cochrane Database of Systematic Reviews and the first 250 results of Google Scholar and Google search engines. This allowed us to cast a wide net in our search for relevant literature. The databases, platforms, and their results are presented in the PRISMA diagram (Figure 1). A detailed search strategy is presented in Multimedia Appendix 1.

We developed a list of search keywords based on seminal sources [2,3,14,26-29] and the National Library of Medicine’s Medical Subject Headings trees to search databases [30,31]. This resulted in a single concept search for “Epic” combined with terms that included “electronic health record*” or “electronic medical record*” or “medical record*” or “health record*” or “EHR“ or “EMR” or “system*.” Keywords and word variants were searched as title and abstract words and subject headings (eg, MEDLINE Medical Subject Headings) as appropriate.

For gray literature, we searched conference proceedings for short or full papers. We excluded conference abstracts from the Embase search because it was not possible to glean the methods and results presented in abstracts alone. In addition, we explored other gray literature sources, including Google Scholar, Google search engines, and the Epic Research site [32]. We did not include articles from the Epic Research site as they varied in detail.

The Covidence (Level 10) web platform was used to import citations and facilitate the screening process [33]. Duplicates were removed using Covidence. Endnote (Clarivate) [34] was used to manage the references.

### Selection of Sources of Evidence

After thorough development of the inclusion and exclusion criteria, we commenced the title and abstract screening processes. Each abstract was reviewed in duplicate by 2 independent reviewers (NS and NW), and conflicts were resolved by a third reviewer (JC) in consultation with others. The full-text articles were screened in the same manner.

### Data Coding and Synthesis of Results

The data abstraction platform was developed in consultation with our multidisciplinary team to capture the breadth of findings from the identified sources. The reviewers piloted the form independently and abstracted 10 full-text articles each. There was excellent agreement between the reviewers on the abstraction form, with some clarification required in defining the research domains and study design. The major categories for abstracting the results included (1) study characteristics (year of publication, country, journal, or conference name); (2) research problem and setting (problem studied, study design, population, and settings); (3) research domains; (4) analytic methods; and (5) specific facilitators and barriers to using Epic for research. We followed the PRISMA-ScR checklist to report the review [25]. The checklist is presented in Multimedia Appendix 2.

### Concepts and Definitions

During our preliminary literature search, we found varying uses of terms, such as the interchangeable use of *EMR* and *EHR*, and related concepts we aimed to cover in the review. Here, we detail the operationalized concepts for clarity.

#### EHR and EMR

EMRs and EHRs are 2 different concepts, which Garets and Davis [3] laid out in their seminal 2006 report. An EMR is a “legal record created in hospitals and ambulatory environments that is the source of data for the EHR,” comprising “an application environment composed of the clinical data repository, clinical decision support, controlled medical vocabulary, order entry, computerized provider order entry, pharmacy, and clinical documentation applications” [3]. An EHR, by contrast, represents the “ability to easily share medical information among stakeholders and to have a patient's information follow him or her through the various modalities of care engaged by that individual” [3]. Stakeholders included patients, caregivers, health care providers, employers, and the government. Hence, an EMR forms the basis for a linked, accessible EHR. For this review, we conceptualize the Epic system as an EHR, as it is currently a client-server-based offering that has a wide variety of functions, with strong linkages to enable access by various clinical stakeholders.

#### HSR and Population Health

We used the Institute of Medicine’s definition of HSR, which describes it as a multidisciplinary field of inquiry that examines access to and the use, costs, quality, delivery, organization, financing, and outcomes of health care services to produce new knowledge about the structure, processes, and effects of health services for individuals and populations” [35].

For population health, we used the expanded definition by Kindig [36] that defines it as “research focused on the health of the entire population, taking into account the environmental, social, and economic factors that determine our health potential.”

#### Registries and Biobanks

We followed the definition of the World Health Organization (WHO) for registries as “documents containing uniform information about individual persons, collected in a systematic and comprehensive way, in order to serve a predetermined purpose” [37]. Biobanks are classically defined as storage of biological samples used specifically for medical research [38].

#### CDS Tools, Clinical, and QI Studies

CDS tools imply a variety of software designed to aid in clinical decision-making, by “matching the characteristics of an individual patient to a computerized clinical knowledge base in order to provide patient-specific assessments or recommendations to the clinician for enhanced evidence-based decision practices” [39].

Clinical studies included those used for improving care and those that involved medical interventions. Some qualified as clinical trials, for which we followed the WHO definition of a “study that prospectively assigns human participants or groups of humans to one or more health-related interventions to evaluate the effects on health outcomes” [40].

For QI papers, we followed the classic work of Donabedian [41], which defines it as “systematic, data-guided activities designed to bring about immediate improvements in health delivery in particular settings.” QI studies are typically related to process improvement.

### Epic Modules and Tools

Epic has various software applications, modules, and tools that can be leveraged for research. We curated information from various Epic websites to gather information on the modules mentioned in the literature and provide a high-level overview of their functions. Not every module we encountered had information available on the Epic website; therefore, additional sources were required to discern the use of some modules and applications.

Epic has a variety of specialty care applications, including ASAP for emergency and urgent care, Beacons for oncology care, Radiant for radiology reporting, tracking, and scheduling, and OpTime for surgery and anesthesia [42]. EpicCare is Epic’s ambulatory and inpatient care EHR.

Epic applications that appear to be particularly suited to research include Cosmos, SlicerDicer, Clarity, Chronicles, Reporting Workbench, and Signal. Cosmos is an aggregate data set of over 217 million patients in the United States that has been used in numerous research applications [43]. SlicerDicer is a data extraction tool within the Epic EHR that facilitates the search and visualization of data in large patient populations [44]. Reporting Workbench, a business intelligence tool, can be used to generate research reports [45]. These reports use Chronicles as their data source, which is a real-time database that runs most Epic software. Data from Chronicles are transferred to Clarity, a Microsoft SQL Server database that can be used for complex reporting [46,47]. The analytics tool Signal allows researchers to observe how providers spend time in the EHR, which can help improve workflow and productivity [48].

Some additional Epic modules and tools include MyChart, Healthy Planet, Care Everywhere, and SmartTools. MyChart is Epic’s web-based patient portal that enables patients to access their health information [49]. Healthy Planet, a population health module, allows providers to monitor patients’ health and care over time, as well as reduce barriers to health by addressing the social determinants of health [50]. Care Everywhere, an interoperability platform, assists with the coordination of care across different providers and health care organizations [51].

### Ethics Approval

Ethics committee approval was not required for this scoping review.

## Results

### Overview

A total of 4669 articles were identified through a combined database and gray literature search. A total of 3469 duplicates were removed by Covidence matching of the titles, abstracts, and article identifiers. Table 1 presents the sources, platforms, and search results.

**Table 1:** Databases and search results (N=4669).

Database	Provider or vendor	Search results, n (%)
MEDLINE	OvidSP	366 (7.83)
Embase	OvidSP	480 (10.28)
CINAHL	EBSCO	225 (4.82)
Web of Science	Clarivate	327 (7)
Scopus	Elsevier	485 (10.39)
Cochrane Library	Wiley	264 (5.65)
Gray literature	Not applicable	2452 (52.52)
Google Scholar and Google internet search	Web sites	70 (1.5)

We screened the titles and abstracts of 1230 articles, of which 626 (50.8%) were excluded. Of the 604 articles included in the full-text review, 397 (65.6%) were excluded according to our criteria. Ultimately, 206 articles were selected for this study. The PRISMA flow diagram is shown in Figure 1.

Of the 206 articles, 174 (83.5%) were peer-reviewed journal articles and 32 (15.5%) were gray literature articles. All gray literature articles were conference proceedings with clear objectives, methods, results, and discussion sections. We could not identify or report on other forms of gray literature because of the lack of detail necessary to qualify as research. We present the results under 5 main headings.

### Year, Country, and Setting

**Figure 2:**
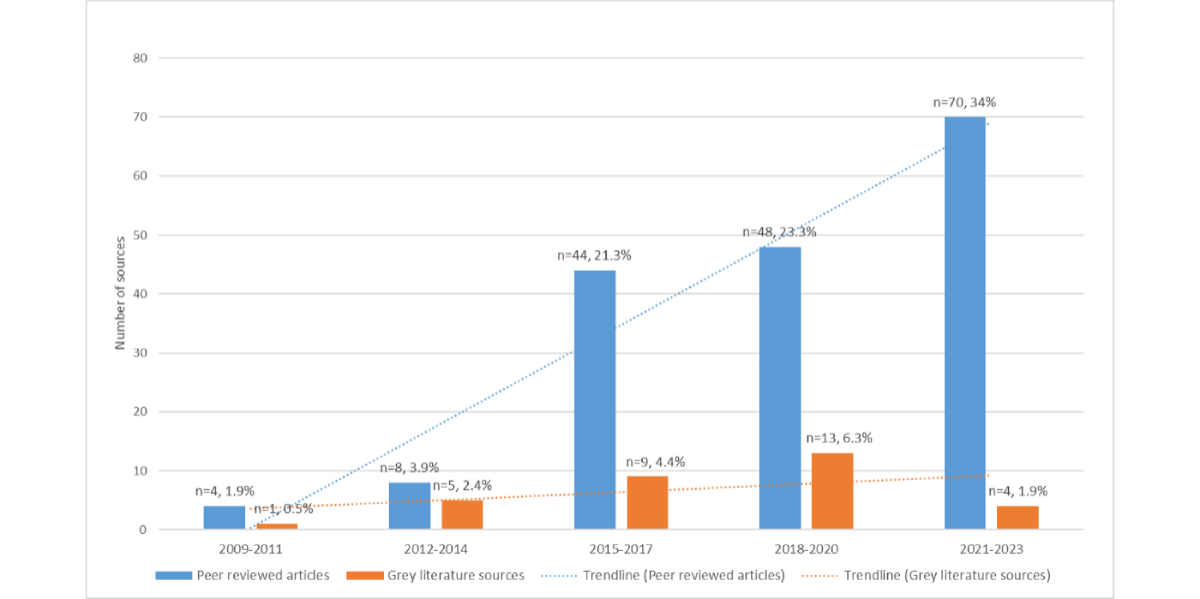
Literature type by year.

**Figure 3:**
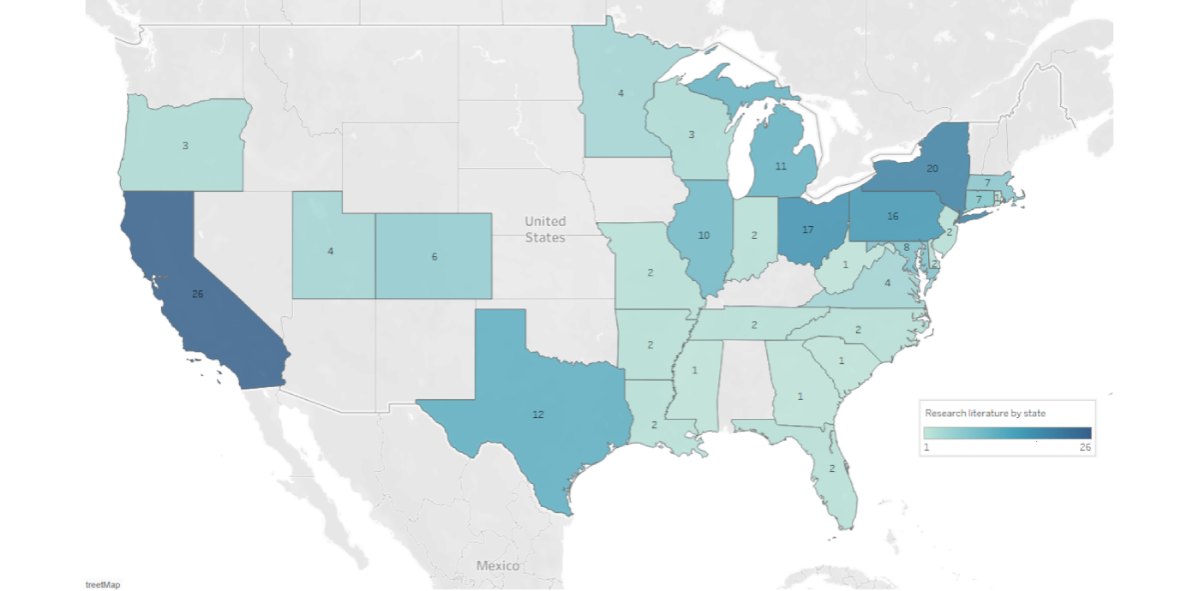
Research literature by US state.

The first articles involving Epic being used for research surfaced in 2009. These trends are shown in Figure 2. Since then, the number of peer-reviewed articles has exponentially increased from 2015 to 2023. The research studies were conducted in the United States (199/206, 96.6%; Figure 3), the Netherlands (3/206, 1.5%), and Saudi Arabia, the United Arab Emirates, Canada, and France (1/206, 0.5% each)

Study settings where the Epic EHR system was used for research included single (133/206, 64.6%) and multiple sites (73/206, 35.4%). As Epic EHR can link across multiple sites, it was difficult to distinguish the number of people and units involved. Single sites included a single department (such as the department of urology or medicine), whereas multiple sites consisted of different practices, departments, and hospitals. Many studies have been conducted in university health systems (44/206, 21.5%), which comprise hospitals of various sizes. Research was also mentioned specifically using Epic EHR data in primary care settings (16/206, 0.1%) and emergency departments and outpatient departments (11/206, 0.1%, both cases).

### Research Domains

The domains of research using the Epic EHR in the literature included clinical research (51/206, 24.8%), HSR (51/206, 24.8%), CDS (45/206, 21.8%), QI (27/206, 13.1%), population health (22/206, 10.7%), registry (8/206, 3.9%), biobanks (1/206, 0.5%), and economic analysis (1/206, 0.5%) emerging as distinct new domains. The research domains, according to the population studied, are presented in Table 2.

**Table 2:** Research domains according to population studied with details of all included articles.

Research domain	Population	Study and year
Biobank	Pediatric	Marsolo et al [52], 2012
**Clinical decision support**		
	Adult	Federman et al [53], 2017; Goehler et al [54], 2019; Horton et al [55], 2018; Howell et al [56], 2014; Klang et al [57], 2021; Li et al [58], 2020; Lindholm et al [59], 2010; Melnick et al [60], 2022; Milne et al [61], 2020; Mitchell et al [62], 2022; Mulhem et al [63], 2020; Park et al [64], 2021; Ritchey et al [65], 2016; Rose et al [66], 2018; Ryan et al [67], 2021; Shelley et al [68], 2017; Sroujieh et al [69], 2016; Toscos et al [70], 2020; Toscos et al [71], 2020; Willis et al [72], 2022
	Adult and pediatric	Del Fiol et al [73], 2020; DeLozier [74], 2021; Jones et al [75], 2022; Jose et al [76], 2020; Keizur et al [77], 2022; Kolb et al [78], 2016; Lilih et al [79], 2017; Nikolic et al [80], 2017; Ray et al [81], 2018; Shelden et al [82], 2021; Siff and Emerman [83], 2016; Sonstein et al [84], 2014; Straub et al [85], 2013; Stutz et al [86], 2018; Tham et al [87], 2016; Wendel et al [88], 2023; Zorn et al [89], 2022
	Not specified	Tsai et al [90], 2015
	Pediatric	Goldberg et al [91], 2016; Hojat et al [92], 2020; Simon et al [93], 2023; Tham et al [87], 2016
	Physicians	Bernstein et al [94], 2017; Nguyen and Fischer [95], 2020; Raja et al [96], 2017
**Clinical research**		
	Adolescent	Snyder et al [97], 2021
	Adult	Adler et al [98], 2019; Alkilany et al [99], 2022; Amaratunga et al [100], 2020; Aslam et al [101], 2019; Boitano et al [102], 2022; Eickholtz et al [103], 2022; Gillen et al [104], 2022; Grigoryan et al [105], 2017; Heidemann et al [106], 2017; Henao et al [107], 2022; Huang et al [108], 2019; Huang et al [109], 2022; Kurin et al [110], 2021; LaHue et al [111], 2022; Martin et al [112], 2017; Milani et al [113], 2017; Mosk et al [114], 2017; Naranjo et al [115], 2021; Narayanan et al [116], 2019; Norton et al [117], 2016; Osterberg et al [118], 2017; Park et al [119], 2022; Pho et al [120], 2019; Putka et al [121], 2009; Rollman et al [122], 2016; Rose et al [123], 2022; Sawalha et al [124], 2021; Stapel et al [125], 2022; Unni et al [126], 2015; Wu et al [127], 2020; Yazdanshenas et al [128], 2022; Zhou et al [129], 2020
	Adult and pediatric	Alzahri [130], 2020; Beck et al [131], 2010; Bhuiyan et al [132], 2018; Dang et al [133], 2016; Erickson et al [134], 2021; Mirro et al [135], 2018; Ram et al [136], 2022; Vemulakonda et al [137], 2017; Vesco et al [138], 2016; Zhang et al [139], 2021
	Pediatric	Brenn et al [140], 2016; Ciricillo et al [141], 2015; Otto [142], 2022; Schleelein et al [143], 2016; Siegfried and Darji [144], 2015; Vemulakonda et al [145], 2022; Williams et al [146], 2019
	Physicians	DeBoer et al [147], 2016
Economic analysis	Adult	Belli et al [148], 2020
**Health services research**		
	Adolescent	Dillon et al 2020 [149], Nolan et al 2020 [150]
	Adult	Austrian et al [151], 2018; Brooks et al [152], 2016; Carter et al [153], 2017; Chiu et al [154], 2018; Clendennen et al [155], 2015; Dowling et al [156], 2018; Flatow et al [157], 2015; Isseh et al [158], 2017; Javier-DesLoges et al [159], 2018; King et al [160], 2020; Loo and Taddei [161], 2015; Makam et al [162], 2013; Mathias et al [163], 2010; McDowell et al [164], 2017; Muqri et al [165], 2022; Nikolian et al [166], 2018; Osband et al [167], 2018; Rizk et al [168], 2020; Rodriguez et al [169], 2021; Salem et al [170], 2023; Serna et al [171], 2022; Su et al [172], 2018; Vlashyn et al [173], 2023; Wagner et al [174], 2015; Zazove et al [175], 2017
	Adult and pediatric	Altice and Gerow [176], 2020; Bellon et al [177], 2015; Burns et al [178], 2022; Everson et al [179], 2016; Handley et al [180], 2022; Nellis et al [181], 2022; Sahu et al [182], 2022; Simon et al [183], 2023
	Not applicable	Palestine et al [184], 2018
	Pediatric	Bush et al [185], 2014; Bush et al [186], 2017; Havrilla et al [187], 2022; Redd et al [188], 2014
	Physicians	Beiser et al [189], 2021; Cheriff et al [190], 2010; Cox et al [191], 2018; Escribe et al [192], 2022; Read-Brown et al [193], 2017; Rove et al [194], 2018; Ruan et al [195], 2022; Wang et al [196], 2019
	Physicians and advanced practice practitioners	Holmgren et al [197], 2022
	Physicians, staff, and patients	Winden et al [198], 2014
	Providers, pediatric patients, and families	Kelly et al [199], 2020
**Population health**		
	Adult	Brant et al [200], 2021; Dhar et al [201], 2019; Egede et al [202], 2021; Kukhareva et al [203], 2022; Mahajan et al [204], 2022; Manzar [205], 2022; McCarthy et al [206], 2021; Mehta et al [207], 2018; Nasehi et al [208], 2018; Patil et al [209], 2022; Reddy et al [210], 2021; Renjithlal et al [211], 2022; Torres et al [212], 2023
	Adult and pediatric	Dinesh et al [213], 2021; Harbison and Gillan [214], 2018; Khanna et al [215], 2021; McCain et al [216], 2022; Rajamani et al [217], 2015; Simmons et al [218], 2022
	Pediatric	Hanna-Attisha et al [219], 2016; Ni et al [220], 2019; Walter et al [221], 2021
**Quality improvement**		
	Adult	Andersen et al [222], 2015; Barclay et al [223], 2019; Behnke et al [224], 2023; Javier-DesLoges et al [225], 2019; Katzan et al [226], 2015; Muñoz et al [227], 2018; Noshad et al [228], 2022; Ter-Minassian et al [229], 2019; van den Broek et al [230], 2021
	Adult and pediatric	Bajracharya et al [231], 2021; Burla et al [232], 2020; Flatow et al [1], 2015; Hensley et al [233], 2019; Kim [234], 2020; Nagi et al [235], 2023; Weng et al [236], 2021; Wu et al [237], 2014; Wyatt et al [238], 2021
	Not specified	Haurani et al [239], 2020
	Pediatric	Koo et al [240], 2020; Lanzo et al [241], 2016; Rameau et al [242], 2018; Wagner et al [243], 2022
	Pediatric and adolescent	Gessner et al [244], 2023
	Physicians	Mehta et al [245], 2016; Wamsley et al [246], 2014
	Providers and clinic staff	Chernitsky et al [247], 2014
**Registry**		
	Adult	Chak et al [248], 2023; Chang et al [249], 2016; Kurian et al [250], 2014; Kuznetsov et al [251], 2013
	Adult and pediatric	Chen et al [252], 2018; Gabel et al [253], 2017; Mou et al [254], 2022
	Not specified	Zetumer et al [255], 2018

Clinical research includes examinations of the prevalence and frequency of clinical conditions and associated factors, as well as the efficacy and safety of clinical interventions. Examples of HSR include investigating the impact of EHR implementation, identifying factors predictive of the use of health services, analyzing EHR use and time spent by clinicians, and measuring health care costs. CDS methods have been used to improve clinical practice, implement screening programs, and increase referrals. QI domain studies assessed the effect of interventions on the quality of care, how to improve access to clinical information, and evaluated quality metrics. Studies in the population health domain have determined factors affecting public health programs and investigated the associations between socioeconomic factors and health. Registries were created and evaluated for use in future studies. The study in the biobank domain discussed the implementation of a biobank using residual clinical samples, whereas the economic analysis focused on behavioral economics to promote appropriate management of a chronic condition.

### Study Design and Population

Longitudinal study designs, including retrospective cohort (85/206, 41.3%) and prospective observational (30/206, 14.6%), were the most common study designs across both peer-reviewed and gray literature. Other designs included pre-post intervention studies (32/206, 15.5%), cross-sectional studies (20/206, 9.7%), and randomized controlled trials (12/206, 5.8%). A variety of other study designs were encountered in the research using the Epic EHR, as presented in Table 3.

**Table 3:** Characteristics of the research studies^a^.

Characteristic		Peer-reviewed articles (n=174), n (%)	Gray literature (n=32), n (%)	Both sources (N=206), n (%)
**Research domains**				
	Clinical research	47 (27)	4 (12.5)	51 (24.8)
	HSR^b^	40 (23)	11 (34.4)	51 (24.8)
	CDS^c^	37 (21.3)	8 (25)	45 (21.8)
	QI^d^	22 (12.6)	0 (0)	27 (13.1)
	Population health	20 (11.5)	7 (21.9)	22 (10.7)
	Registry	6 (3.4)	2 (6.3)	8 (3.9)
	Biobank	1 (0.6)	0 (0)	1 (0.5)
	Economic analysis	1 (0.6)	0 (0)	1 (0.5)
**Study design**				
	Retrospective cohort	73 (42)	12 (37.5)	85 (41.3)
	Prospective	24 (13.8)	6 (18.8)	30 (14.6)
	Pre-post intervention	24 (13.8)	8 (25)	32 (15.5)
	Cross section	19 (10.9)	1 (3.1)	20 (9.7)
	RCT^e^	11 (6.3)	1 (3.1)	12 (5.8)
	Case control	5 (2.9)	0 (0)	5 (2.4)
	Case report	4 (2.3)	0 (0)	4 (1.9)
	Quasi-experimental	3 (1.7)	1 (3.1)	4 (1.9)
	Phenotyping	3 (1.7)	0 (0)	3 (1.5)
	Mixed methods	3 (1.7)	0 (0)	3 (1.5)
	Time series	2 (1.1)	1 (3.1)	3 (1.5)
	Ethnographic	1 (0.6)	0 (0)	1 (0.5)
	Cognitive task analysis	1 (0.6)	0 (0)	1 (0.5)
	Cost analysis	1 (0.6)	0 (0)	1 (0.5)
	Chart review	0 (0)	2 (6.3)	2 (1)
**Study setting**				
	Single site	65 (37.4)	24 (75)	133 (64.6)
	Multisite	109 (62.6)	8 (25)	73 (35.4)
**Population**				
	Adult	89 (51.1)	15 (46.9)	104 (50.5)
	Adult and pediatric	44 (25.3)	9 (28.1)	53 (25.7)
	Pediatric	21 (12.1)	2 (6.3)	23 (11.2)
	Physicians	11 (6.3)	3 (9.4)	14 (6.8)
	Adolescent	3 (1.7)	0 (0)	3 (1.5)
	Physicians, practitioners, staff, and patients	3 (1.7)	1 (3.1)	4 (0.5)
	Pediatric and adolescent	1 (0.6)	0 (0)	1 (0.5)
	Not applicable or specified	2 (1.1)	2 (9.4)	4 (1.9)

^a^Percentages may not necessarily total 100% due to rounding.

^b^HSR: health services research.

^c^CDS: clinical decision support.

^d^QI: quality improvement.

^e^RCT: randomized controlled trial.

The populations studied using the Epic EHR were primarily adults (104/206, 50.5%) or combined adult and pediatric populations (53/206, 25.7%). There were 14 studies (6.8%) researching physician users, which examined their use of the Epic EHR and its various functions and tools, including CDS tools. The specialties of physicians studied varied and included primary care (3/14, 21%), internal medicine (4/14, 29%), and multispecialty physician research (3/14, 21%). The remaining studies focused on pediatric patients (23/206, 11.2%), adolescents (3/206, 1.5%), adolescents and pediatrics (1/206, 0.5%), and other health care staff and patients (4/206, 1.9%; Table 3).

### Analytic Methods

The analytic methods included descriptive statistics (70/206, 34%), multivariable regression (61/206, 29.6%), development and testing of an algorithm (6/206, 2.9%), and machine learning methods (2/206, 1%). One qualitative study used ethnographic methods and another used economic analysis. Further details of the analytical methods used are presented in Multimedia Appendix 3.

### Modules and Tools

Most studies did not use or did not mention using a specific module or application within the Epic EHR for research purposes (161/206, 78.2%). Among the studies that indicated the Epic modules used (45/206, 21.8%), tools included MyChart (10/45, 22%), Clarity (10/45, 22%), SlicerDicer (6/45, 13%), and SmartTools (6/45, 13%). Other modules used were EpicCare for ambulatory and inpatient care (3/45, 7%), ASAP for the emergency room (2/45, 4%), Optime for surgery and anesthesia (2/45, 4%), Beacon for medical oncology (2/45, 4%), and Radiant for radiology (1/45, 2%). Additional Epic modules and applications reported included Healthy Planet (1/45, 2%), Care Everywhere (3/45, 7%), Cosmos (1/45, 2%), Chronicles (1/45, 2%), Reporting Workbench (1/45, 2%), and Signal (2/45, 4%). Two studies reported the use of multiple Epic modules (2/45, 4%).

### Facilitators and Barriers to Using Epic for Research

Related to its use in research, we examined the literature for facilitators and barriers that could be helpful for researchers considering the use of Epic EHR data or module features. Facilitators for using the Epic EHR included developing guideline-based clinical order sets, pooling survey resources into dashboards, using the SlicerDicer visualization tool used for presenting data, identifying cohorts, physician prompts, audit logs for user interaction, and use of the EHR for patient education, and access to data by various audiences, including patients.

Studies listing barriers to the use of the Epic EHR for research discussed inaccurate coding and missing data, missing specific terminology for the detection of particular conditions, underreported social determinants of health and patient-reported outcomes, lack of documentation of mental health issues, dependence on diagnostic codes within Epic to flag conditions and reports, and variation in the use of the EHR by staff, physicians, and across teams.

## Discussion

### Value-Add From the Scoping Review

As scoping reviews capture the breadth of literature [21], this review presents the current state of science on the use of the Epic EHR for research in medicine and health sciences. Currently, reviews related to Epic implementation [13], transition [8], and integration to the Epic EHR for personalized care [14] are available. In addition, our search for systematic and narrative reviews on the same topic related to other major EHRs in use, such as Cerner, could not identify any peer-reviewed published work, at least in the last 2 decades. Hence, this scoping review attempts to fill a critical gap in the literature, showcasing trends, opportunities, and issues in the use of Epic EHR for healthcare research. We discuss and contrast our findings with considerations and recommendations that would be helpful for researchers and practitioners intending to use EHRs as data sources or research tools.

### Current State and Opportunities

We found that Epic EHR has been related to a plethora of research and analytic tools that can be used in a wide variety of ways, including for different study designs and clinical populations, with an increase in the use of studies from 2015 onwards. This increase could be due to EHR enhancements, better understanding of EHR data among researchers, and improved data access. We also found that most studies were from the United States, which could be due to the high uptake of Epic in the country, and capital and recurring costs related to the implementation and maintenance of EHRs.

Epic’s architecture allows the integration of different data sources, including large medical and health databases, population-based registries, and patient-reported data [53,250,256]. This is an important capability that allows linkage and access to administrative and clinical data [53,186,257-259]. Linkage enhances the capacity to discover patient care patterns within and between hospitals and community care providers [249]. In addition, deidentified data can be exported for analysis [185]. Epic can also be used to scale interventions at multiple sites, thereby expanding research capabilities across geographic boundaries [155]. Furthermore, studies point to the advantage afforded by EHRs as a cheaper clinical data source that can be used for research [18].

Among the Epic EHR tools, MyChart, SlicerDicer, and Clarity were the most frequently used. For the research domains of their use, half of MyChart belonged to HSR, whereas others included clinical research, CDS, and QI. Of the studies that reported the use of SmartTools, half belonged to CDS, with the remainder in the QI and clinical research domains. Clarity and SlicerDicer have been used in various research domains. The most reported modules were EpicCare for ambulatory and inpatient care, ASAP for the emergency room, Optime for surgery and anesthesia, and Beacon for medical oncology.

An earlier narrative review of EHRs identified multivariate modeling as the most common method for targeting confounding and selection bias [17]. Epic’s built-in applications and features, CDS tools, and predictive analytic algorithms can guide clinical management and provide a wide variety of opportunities for data analysis and reporting [114,155,159,260,261]. In this review, the most used analytical methods were descriptive statistics, closely followed by multivariable regression modeling, developing algorithms, and other machine learning methods. The least reported methods include ethnographic and economic analysis.

From 2015 onwards, we observed an increasing trend in the use of Epic EHR in population-based studies. An earlier narrative review focusing on population health applications using EHRs also identified diverse research, including cross-sectional and longitudinal studies [18]. A greater number of population-based studies could be due to a better understanding of EHR data in general among epidemiological researchers. For example, one seminal study reported observational data related to the Flint, Michigan, drinking water crisis, which resulted in both local and national population health policy changes [219]. Such studies, although rare, are excellent examples of not only generating and using research data but also translating evidence toward policy change.

With the rapid growth in the use of EHRs, a rise in population health-related research from EHR data can be expected in the near future. Disease surveillance is also a growing area, with examples from preconception screening [85], immunization [92], hypertension, and obesity [262].

Epic allows for the development, implementation, and validation of CDS tools to improve quality of care [55,66,260,263]. In addition, in-system evaluation is possible within the EHR [59]. For clinical research, tools offer support for trials, cohort selection, recruitment, randomization, and data collection [87,114]. QI initiatives using the Epic EHR focused on best practice advisories and workflows [222,245,264,265], reducing prescription and medication dosing errors [266,267], and linking data from health apps to the Epic system [256], which shows the potential for integration of various mobile and eHealth apps. Another important capability of the Epic EHR is reporting patient-reported outcomes, which is enhanced by the ability to integrate outcome assessment tools within the personal health portal [268]. Additional functionality for improving education and communication tools for patients and health care professionals has also been noted [246,269].

From 2021 onwards, we identified 15 COVID-19–related studies [88,101,112,125,128,137,181,189,195,202,209,213,215,216,244]. These ranged from descriptive studies on disparities due to social determinants of health, the association of the virus with other conditions, severity in specific groups, provision of virtual care, treatment options, and changes in physician behavior.

Other varied but little-used research functions using Epic EHR included developing registries, biobanks, and an economic analysis. For example, Stanford University’s breast cancer registry was created by linking 2 independent institutions to identify interventions including surgery, chemotherapy, and diagnostics [250]. We found only one study using the EHR for economic analysis [149] and one for creating a biobank [52]. This points to the opportunity afforded by such a diverse system, as economic analyses are traditionally conducted using administrative data.

### Considerations for Researchers and Practitioners

#### Overview

Although the versatility of the Epic EHR provides extensive opportunities for research, important barriers and challenges warrant consideration for the use of the Epic EHR for research. We contrast our findings with the literature on this subject. It is important to note that these considerations may not be limited to the Epic EHR alone, as issues of data quality, completeness, and accuracy are common [270].

#### Architecture and IT

Architecture and IT issues are related to the consistent use, provider behavior, and flexibility afforded by any EHR system. Epic is currently a client-server–based EHR that offers institutions centralized control of infrastructure and data management [271]. To be successfully used for research, EHRs must be aligned within the complex health system environment where technology health care personnel and patients interact [272]. There can be ongoing costs when there are changes required in the original system setup by appropriate staff [68,167,249].

Furthermore, issues are expected when integrating data with differing or incompatible storage structures, systems, or domain ontologies [87,184]. Such infrastructure issues could make it difficult to transfer innovations from one Epic installation to another [87]. In addition, intuitive interface designs and continued IT support for troubleshooting communications and hardware are known issues [56,176,249,256]. In particular, CDS tools have usability issues, such as formatting and editing challenges, and the need to fill out unnecessary fields that can become barriers to clinicians for data collection [68,176].

#### Data Governance, Access, and Quality

As with all EHR-based research, data governance and quality remain important issues for ensuring information privacy and security. Related common challenges for Epic include barriers to data access, variable data quality, and difficulties in effectively and efficiently handling data for research, including maintaining rigor and consistency across multicenter trials [87]. In this case, Epic’s flexibility for use may be working against it, as users customize the offering based on individual and site needs.

One of the greatest challenges relates to new implementations, particularly where secondary data access has not been considered in the original build [176]. Organizational processes can also hinder access. For instance, preexisting data access and data sharing policies, as well as organizational precedents for secondary data use, may not acknowledge the highly identifiable electronic data used for research or QI [217].

Assessing data quality for research use can prove onerous because of the diversity in data elements, study settings, populations, and clinical conditions across EHR implementations [180,185,250,273]. Diverse settings and users can create serious validity and reliability issues, resulting in the need to design complex algorithms for mining multiple data fields [186]. In an earlier narrative review, a quarter of the identified studies focused on data validation [17].

In addition, analyzing unstructured medical notes requires advanced data analysis skills [250,274]. Finally, if data are extracted from different sites or geographic regions, the quality of the data depends on convergent timelines and alignment with existing administrative and clinical systems [68,241]. These differences can result in incongruent, varied, and missing information [176].

#### Patient-Centered Care

EHRs can affect patient interactions and have been found to both positively and negatively influence patient-provider relationships [268,275]. We found that although most studies highlighted improvements in communication between patients, providers, and researchers, challenges for consumers or patients included lack of computer skills, internet access, and the need to communicate directly with providers rather than through a web-based portal [268]. In particular, studies on patient engagement have reported general satisfaction from respondents relating to confidentiality and monitoring of care [268]. Epic systems also facilitate automated electronic secure messaging via Epic’s MyChart to confirm patient appointments, conduct prescreens, and share information [52,165,268,276]. Collecting patient consent was mentioned as a key benefit, without the need for dedicated research staff.

### Recommendations for Future Use of Epic for Research

On the basis of the opportunities and challenges outlined above, Table 4 summarizes the key considerations and recommendations that can be useful for future research applications. We highlight key aspects and recommendations according to the 5 research domains.

**Table 4:** Considerations and recommendations for conducting research in Epic clinical information system.

Research domain	Epic capabilities	Recommendations
Clinical research	Ability to collect and use data across geographic boundaries (comparative multicenter clinical trials) Potential for custom recruitment alerts for physicians Ability to engage directly with patients through the patient portal (prescreens, research recruitment, referrals, consents, surveys, and knowledge translation)	Identify a priori differences in data and system builds across clinical sites Establish strong governance processes and policies before system build Conduct usability studies on an ongoing basis Provide and tailor training and analytic support Build in funding within studies for system build and changes
Health services research	Ability to integrate data from external devices and applications Capacity to integrate complementary data from large databases, EHRs, and population‐based registries Ability to use predictive analytics, natural language processing, and machine learning Capacity to build patient-reported outcome measures and instruments, with user roles and assigned tasks Automated conversion of diagnostic codes	Train analysts on available discrete data elements and challenges with extensive missing data Ongoing training for complete documentation within important notes Budget for additional analytic support and user training Establish strong governance processes and policies, especially secondary data access and use before system build Continue to train personnel on avoiding note bloat and optimization
Clinical decision support	Standardized tools that can be used at multiple sites Adaptive design and validation Developing custom interfaces Autopopulating patient data	Maintain continuous IT support Obtain clinician buy-in Work with clinicians to avoid fatigue Provide training and analytic support Secure upfront financial investment
Population health	Ability to discover care patterns within and between community and hospital patients, and between health systems Enhanced population surveillance Ability to integrate data from geographic positioning systems Enhanced accessibility of clinical information, including medical images and genomic sequencing tests Market dominance of Epic offers an opportunity for better data integration	Institute organizational processes to facilitate data access Train analysts on handling and reporting available discrete data elements and challenges with extensive missing data (or lack of documentation)
Quality improvement	Adaptable 5 platforms with ability to make rapid changes Ability to access large patient data sets Access to a variety of templates and the ability to generate clinical summaries and reports Ability to create worksheets and checklists	Create a software development team to become familiar with clinical data for care and research Navigate difficulties with the user interface and usability Anticipate and mitigate data integration complications Provide clinician training and IT support
Registries and biobanks	Ability for automatic access to inpatient and outpatient data in all phases of care Ability to embed consent processes, without dedicated staff Ability to create custom interfaces Easy to transfer deidentified data for analysis	Establish strong governance processes and policies before building the system (particularly secondary data access and use) Secure upfront financial investment Provide staff and user training and analytic support

#### Training and Education

##### Overview

EHR implementation presents several challenges, particularly in relation to the ongoing provision of education and training. Researchers, clinicians, and other health care providers may be unaware of EHR data function and structure, storage, use, and limitations [277]. Clinicians’ time is precious, and extensive training may not be feasible or efficient. Finally, there may be a paucity of expertise and absence of validated methodologies for using unstructured data. These challenges highlight the need for investments in continued education and capacity-building.

User behavior and experience are related to the completeness, consistency, and quality of the data. In the case of an EHR, physician input is essential for generating clinical data. Limited user experience and lack of confidence are often cited as barriers to system use and the correct collection and interpretation of data [265]. Additionally, users may be unfamiliar with many system functions, structures, and sources of clinical data. To address these challenges, a ramp-up period of approximately 6 months results in an incremental increase in productivity for clinical practitioners [190]. In addition, site-specific training information and modules that are incorporated help increase knowledge access [278].

##### Policy and Governance

Access to granular patient-level data is required to inform research [279]. Consequently, policies regulating data access and use should ideally be implemented before the implementation of any EHR [280]. Epic implementation sites may have preexisting data access and sharing policies and precedents for secondary data use, which may not acknowledge the value of these data for the purpose of continuous QI or research in general. In addition, there may be differences in policies and processes between data custodians, local health authorities, and university ethics boards regarding how data are provisioned, handled, and stored.

User roles, such as “super users” and “physician builders,” are assigned to individuals within organizations implementing the Epic EHR. Researchers, university personnel, and staff affiliated with health care settings may not be included as system users, which can be a serious hindrance in building research capacity using EHR data. Hence, early access and training for future researchers involved in health system improvement should be considered.

##### Importance of Transinstitutional Partnerships

For effective research, partnerships with health authorities, universities, and government organizations to strengthen research capacity using any EHR should not be overemphasized. These partnership teams could provide input regarding direction setting, validation, testing, and deployment of EHR systems across institutions and health care organizations. Partnerships can be developed through working groups, with the primary goal of ensuring value for both frontline clinical use and secondary data research through the creation of site-specific documentation and resources [163]. Further, such working groups could aid in developing sample datasets, identifying security and privacy issues, laying down governance structures for accessibility, and paving the way for analytic approaches [186]. Finally, working groups can advocate researchers and clinicians affiliated with universities and other learning organizations to create a large pool of super users and physician builders.

Partnerships with local health systems can create opportunities to improve system performance and the quality of care [186]. In addition, these partnerships can enhance the codevelopment of performance indicators. Finally, having a transinstitutional working group could enhance communication among researchers, clinicians, and IT departments in both universities and local health authorities to enhance data extraction, management, and use.

### Generalizability of Findings and Future Research

Although our review focuses on the use of the Epic EHR as a widely adopted system, the major lessons, facilitators, and barriers can be appreciated, compared, and generalized to other EHR systems. Researchers and practitioners can be better aware of the known and potential opportunities and challenges related to the use of an EHR system for research. We contend that our study is not only timely, but also highly relevant for health care institutions, as 8 out of 10 instances of EHR implementation involve an Epic EHR offering. As we observed, this body of literature is expanding rapidly as Epic sites become better established with the provision of collaborative research support, education, and governance processes.

Future research can inform many directions for the use of EHR data, not only for Epic EHR, but also for other major EHRs. There is a critical need for updated information on the use of EHRs and data and newer methodologies, such as the use of machine learning methods, particularly natural language processing [281], and visual analytic methods for knowledge generation and decision support [282,283]. Although EHR clinical notes are gold mines for research, data quality, completeness, and accuracy remain major challenges for which emerging methodologies for validation and reducing variability are important areas of research.

### Limitations

As a scoping review, this paper presents an overview of a large and diverse body of literature pertaining to areas of research using Epic EHR. There are several limitations to consider, especially pertaining to the feasibility of any scoping review. Although we conducted a broad search of the literature using databases and gray literature sources, we could not include articles from languages other than English. To improve the completeness of the literature, we diligently searched for and abstracted full-text articles that were previously published as conference proceedings. Thus, we were able to include updated information with key lessons for researchers and practitioners. As scoping reviews mainly aims to capture the breadth of literature, assessing the quality of literature is optional. For this review, we did not assess the quality of the literature; however, given our wide search of the databases and inclusion of mostly peer-reviewed literature, we are reasonably sure that the literature included in the review is of high quality.

As strengths, we employed established scoping review methods, along with constituting a multidisciplinary team, including an information specialist, to guide the research question, search strategy, and operationalize the scoping review. We also spent considerable time and effort in conceptualizing and reaching a consensus on the research domains and presented our results in a manner that would illustrate the breadth of literature, while making it more accessible.

### Conclusions

EHRs are a new source of health data with an ever-expanding repertoire of functions. This makes them a unique tool for primary and secondary research. Epic is a globally known, well-established, and growing EHR. This review attempts to fill a critical gap in the literature regarding the use of EHRs for research, outlining the current state of science in this field. In addition, we offer opportunities, key considerations, and recommendations for the future use of Epic and other EHRs.

## Appendix

Multimedia Appendix 1 Search strategy for scoping review.

Multimedia Appendix 2 PRISMA-ScR (Preferred Reporting Items for Systematic Reviews and Meta-Analyses extension for Scoping Reviews) checklist.

Multimedia Appendix 3 Detailed results.

## Abbreviations

CDS: clinical decision support
EHR: electronic health record
EMR: electronic medical record
HSR: health services research
QI: quality improvement
PRISMA-ScR: Preferred Reporting Items for Systematic Reviews and Meta-Analyses extension for Scoping Reviews
WHO: World Health Organization
