# Supplementary material for: Use of Epic Electronic Health Record System for Health Care Research: Scoping Review
Source: J Med Internet Res. 2023 Dec 15;25:e51003. doi: 10.2196/51003 (PMC10757236; doi:10.2196/51003)
Supplement: Multimedia Appendix 1 [file jmir_v25i1e51003_app1.docx]

Multimedia Appendix 1: Search Strategy

**Date: April 28 2023**

**Database: Ovid MEDLINE(R) and Epub Ahead of Print, In-Process, In-Data-Review & Other Non-Indexed Citations and Daily <1946 to April 25, 2023>**
**Search Strategy:**
**1**  (EPIC adj10 (electronic health record* or electronic medical record* or medical record* or health record* or EHR or EMR or system*)).tw,kf. (577)
**2**  limit 1 to english language (**472)**

**Database: Embase <1974 to 2023 April 26>**
**Search Strategy:**
**1**  (EPIC adj10 (electronic health record* or electronic medical record* or medical record* or health record* or EHR or EMR or system*)).tw,kf. (1932)
**2**  limit 1 to english language (1927)
**3**  limit 2 to conference abstracts (1447)
**4**  2 not 3 (**480**)

**CINAHL**

TI ( (EPIC N10 (electronic health record* or electronic medical record* or medical record* or health record* or EHR or EMR or system*) ) OR AB ( (EPIC N10 (electronic health record* or electronic medical record* or medical record* or health record* or EHR or EMR or system*) )

Limit to English Language, Articles

**Total: 225**

**Cochrane Library (Wiley)**

((EPIC NEAR/10 (electronic health record* or electronic medical record* or medical record* or health record* or EHR or EMR or system*))):ti,ab,kw (Word variations have been searched)

**Total: 254**

**Scopus**

TITLE-ABS-KEY ( epic  W/10  ( "electronic health record*"  OR  "electronic medical record*"  OR  "medical record*"  OR  "health record*"  OR  ehr  OR  emr ) )  AND  ( LIMIT-TO ( DOCTYPE ,  "ar" ) )  AND  ( LIMIT-TO ( LANGUAGE ,  "English" ) )

**Total: 284**

TITLE-ABS-KEY ( epic  W/3  ( system  OR  systems ) )  AND  ( LIMIT-TO ( LANGUAGE ,  "English" ) )  AND  ( LIMIT-TO ( DOCTYPE ,  "ar" ) )  AND  ( LIMIT-TO ( SUBJAREA ,  "MEDI" )  OR  LIMIT-TO ( SUBJAREA ,  "HEAL" )  OR  LIMIT-TO ( SUBJAREA ,  "PHAR" )  OR  LIMIT-TO ( SUBJAREA ,  "NURS" )  OR  LIMIT-TO ( SUBJAREA ,  "NEUR" )  OR  LIMIT-TO ( SUBJAREA ,  "IMMU" ) )

**Total: 201**

**Web of Science**

TS=(EPIC NEAR/10 ("electronic health record*" or "electronic medical record*" or "medical record*" or "health record*" or EHR or EMR ))

Limit to Articles or Proceedings Papers

Limit to English Language

**Total: 261**
