# Supplementary material for: Use of Epic Electronic Health Record System for Health Care Research: Scoping Review
Source: J Med Internet Res. 2023 Dec 15;25:e51003. doi: 10.2196/51003 (PMC10757236; doi:10.2196/51003)
Supplement: Multimedia Appendix 3 [file jmir_v25i1e51003_app3.docx]

# **Supplementary File 3: Main abstraction table with details of research studies that used the EPIC EHR**

|  | **Author and year** | **Ref** | **Country** | **US**  **State** | **Research problem** | **Domain** | **Population** | **Settings** | **Design** | **Analytic methods** | **Major findings** | **EPIC EHR related facilitators** | **EPIC EHR related barriers** | **EPIC module(s) used** |
| --- | --- | --- | --- | --- | --- | --- | --- | --- | --- | --- | --- | --- | --- | --- |
|  | Adler 2019 | (1) | USA | NY | Determine factors for responding to behavioral intervention to enhance cancer screening | Clinical Research | Adult | Single site | RCT | Descriptive and comparative statistics | Factors associated with non-adherence included Hispanic/Latina ethnicity, lack of health insurance, lack of a normal provider of women’s health care, and cigarette smoking | None stated | None stated. | None stated. |
|  | Alkilany 2022 | (2) | USA | OH | Evaluate uric acid (UA) level and the use of urate-lowering therapies (ULTs) in patients with gout and end-stage renal disease (ESRD) on hemodialysis (HD). | Clinical Research | Adult | Multi-site | Retrospective cohort | Single-sample *t* test, paired *t* test. | Initiation of hemodialysis is associated with a decrease in uric acid levels in end-stage renal disease patients. Need for improved monitoring of gout (serum UA and urate-lowering therapies) in ESRD patients. | None stated | None stated | None stated. |
|  | Altice 2020 | (3) | USA | VA | Compare outcomes of heart failure patients attending and not attending cardiac rehab after 8 weeks. | HSR | Adult and pediatric | Multi-site | Quasi-experimental | Descriptive statistics, chi-square test. | Statistically significant improvement in 30-day and 6 week all cause readmission rates, and 6-week heart failure in the cardiac rehab group, supporting early access to cardiac rehab in reducing rehospitalization rates. | None stated | None stated | None stated. |
|  | Alzahri 2020 | (4) | Saudi Arabia | N/A | serum creatine kinase (CK) measurement as a predictor of acute kidney injury (AKI) in emergency department (ED) patients who present with possible substance-use related conditions. | Clinical Research | Adult and pediatric | Single site | Retrospective cohort | Descriptive statistics, univariate logistic regression | No identifiable CK level associated with AKI risk. Limited value in repeated CK measurements. | None stated | Pull down problem list and history section of Epic EMR was often skipped by ED clinicians | ASAP ER Module |
|  | Amaratunga 2020 | (5) | USA | PA | Evaluate the association between COVID-19 and bradycardia. | Clinical Research | Adult | Single site | Case report | Descriptive statistics | Transient sinus bradycardia is a possible manifestation of COVID-19. | None stated | None stated | None stated. |
|  | Andersen 2015 | (6) | USA | PA | Familial hypercholesterolemia (FH) community initiative using EHRs (EHR) | QI | Adult | Single site | Pre-post intervention | Descriptive analysis | Direct outreach method was successful in increasing the identification of the condition in the community | None stated | None stated | None stated. |
|  | Aslam 2019 | (7) | USA | IL | Determine treatment patterns for clostridium difficile in hospital | Clinical Research | Adult | Single site | Retrospective cohort | Descriptive statistics, Welch's t-test | Compliance to treatment guidelines was poor and barriers to compliance still exist despite education and guideline availability | Developed guidelines-based order sets in Epic from data pulled from the EMR | None stated. | None stated. |
|  | Austrian 2018 | (8) | USA | NY | Evaluate financial and clinical impact of a surgery pathway integrated in the EHR. | HSR | Adult | Multi-site | Retrospective cohort | Interrupted time series, segmented regression analysis, gamma regression, logistic models, difference-in-difference. | Statistically significant decrease in costs in the colon surgery cohort. No significant changes in 30 day readmission rates. | None stated | None stated | None stated. |
|  | Bajracharya 2021 | (9) | USA | MA | Implement a patient engagement intervention to monitor and improve the care of diabetic patients. | QI | Adult and pediatric | Multi-site | Prospective | Descriptive statistics | Pilot demonstrated the potential to identify patients with gaps in diabetes care who could benefit from outreach specialist support. | EPIC EHR reporting workbench used to identify patients with gaps in recommended diabetes care & self-management. | None stated | None stated. |
|  | Barclay 2019 | (10) | USA | NC | Develop and evaluate screening and intervention for people with alcohol abuse | QI | Adult | Single site | Mixed methods | Descriptive statistics, survey of key informants | Intervention resulted in screening rates exceeding national averages; key informants rated the Epic tools as positive | Prior EHR tool builds for other services with similar workflow and EHR functionality helped the development team efficiently produce our project’s tools. | None stated. | Smart Tools |
|  | Beck 2010 | (11) | USA | PA | Identify the incidence of scapholunate (SL) dissociation in aspiration-confirmed gout of the wrist | Clinical Research | Adult and pediatric | Multi-site | Retrospective cohort | Retrospective chart review | SL dissociation associated with gouty arthropathy of wrist; 60% of patients using definition 1 and 25% of patients using definition 2. Incidence decreases with a more strict definition. | None stated | Does not account for inaccurate coding of each encounter. | None stated. |
|  | Behnke 2023 | (12) | USA | VA | Evaluate an intervention in patients with elevated serum calcium to screen for undiagnosed primary hyperparathyroidism. | QI | Adult | Single site | Pre-post intervention | None mentioned | Sending a letter to patients with elevated serum calcium advising them to discuss PTH testing with their primary care provider had clinically significant results, with 5.1% of these patients having a PTH test in the follow-up period. | EPIC was queried to find patients with elevated serum calcium and no PTH results. | None stated | None stated. |
|  | Beiser 2021 | (13) | USA | NY | Evaluate the effect of telemedicine implementation during the COVID-19 pandemic on provider experience. | HSR | Physicians | Single site | Pre-post intervention | Descriptive statistics | Post telemedicine implementation, providers had fewer appointments per day, spent less time in the EHR outside normal working errors, and closed more notes on the same day. | EPIC signal used to collect data on actions logged in EHR. | None stated | Signal |
|  | Belli 2020 | (14) | USA | NY | Develop and test a behavioral economics EHR module to promote recommended management of diabetes. | Economic Analysis | Adult | Multi-site | Pre-post intervention | Descriptive statistics; compliance rates, utilization rates | Decision support tool improved adherence to clinical guidelines for diabetes management. | CDS tool can be built and integrated into Epic as a module | None stated | None stated. |
|  | Bellon 2015 | (15) | USA | PA | Compare two e-visit models and their providers as well as associated outcomes. | HSR | Adult and pediatric | Single site | Retrospective cohort | Cross-frequency tables, multivariate analyses, t-test, chi-squared, kolmogorov-smirnov goodness of fit, linear regression. | Users of e-services more likely to be female, white, married. Continuity of care may be less important for younger, single, high income patients than convenience. | None stated | None stated | MyChart |
|  | Bernstein 2017 | (16) | USA | CT | Describe the impact and use of a clinical decision support tool addressing tobacco dependence for inpatients. | CDS | Physicians | Multi-site | RCT | Logistic regression, Spearman's correlation coefficient | Physicians assigned to CDS intervention were more likely to order tobacco treatment medication, add tobacco use disorder to the patient problem list, and make a referral to the state smoker's quitline. | CDS and order sets embedded in EPIC EHR. | None stated. | None stated. |
|  | Bhuiyan 2018 | (17) | USA | MD | Assess the effects of adding allopurionol to standard maintenance therapy in acute lymphoblastic leukemia and lymphoblastic lymphoma patients. | Clinical Research | Adult and pediatric | Single site | Prospective | None stated. | Initial results show allopurinol is associated with a decrease in hepatotoxcity, toxic metabolite ratio, and required 6-MP dose. | None stated | None stated. | None stated. |
|  | Boitano 2022 | (18) | USA | MA | Evaluate the outcome of a nurse-navigator run program which uses a natural language processing (NLP) algorithm applied to the EHR to identify patients with abdominal aortic aneurysm (AAA) who are not being actively followed. | Clinical Research | Adult | Single site | Retrospective cohort | None stated | The NLP AAA program successfully identified patients with AAA who were not receiving appropriate surveilliance, repair, and counselling. | None stated | Imaging report needs to discuss the aneurysm or aortic diameter for the NLP program to flag it. | None stated. |
|  | Brant 2021 | (19) | USA | OH | Assess the rates of Long-Acting Reversible Contraceptive (LARC) use in the immediate postpartum period before and after a state policy mandating inpatient access. | Population Health | Adult | Multi-site | Case control | Descriptive statistics, two-sample t test, Wilcoxon rank sum test, Pearson's chi-square test, Fisher exact test, univariate and multivariable logistic models. | After the state policy change, LARC use increased and resulted in decreased pregnancy rates in the first year post delivery. | None stated | None stated | None stated. |
|  | Brenn 2016 | (20) | USA | DE | Determine rate of post-operative complications, and satisfaction with quality of care. | Clinical Research | Pediatric | Single site | Prospective | Chi-square test, phi coefficient. | Pain most frequently reported complication. 0.31% overall dissatisfaction, with "time issues" being second largest category of dissatisfiers. Minimal relationship between complication and dissatisfaction. | Survey responses pooled into dashboards in Epic. | None stated | Optime |
|  | Brooks 2016 | (21) | USA | MA | A pragmatic approach for measuring and monitoring hospitalizations in patients receiving chemotherapy for pancreatic cancer | HSR | Adult | Single site | Time series | Descriptive analysis | EHRs provide efficient approaches for monitoring monthly hospitalization across chemotherapy patients. | None stated | None stated | None stated. |
|  | Burla 2020 | (22) | USA | MI | Compare the difference in clinical outcomes and completion of sepsis core measures when a resuscitation resident is involved versus not involved. | QI | Adult and pediatric | Single site | Quasi-experimental | Descriptive statistics, chi-square test, Fisher's exact test, two-sided t-test, Wilcoxon rank-sum test. | Trained resuscitation resident involvement was associated with improvement in various sepsis core measures (such as time for lactic acid draw) but not mortality outcomes. | None stated | None stated | None stated. |
|  | Burns 2022 | (23) | USA | Multistate | The impact of modifiable risk factors on total encounter charges, LOS, & related emergency department visits/hospital admissions within 90 days after shoulder arthroplasty. | HSR | Adult and pediatric | Multi-site | Retrospective cohort | Linear regression, poisson regression & logistic regression | Anemia, malnutrition, and obesity contribute to increased costs after shoulder arthroplasty. LOS was higher in patients with anemia, malnutrition and uncontrolled diabetes. | None stated | None stated | None stated. |
|  | Bush 2014 | (24) | USA | Multistate | To use EHR data to determine factors predictive of patient no-shows. | HSR | Pediatric | Multi-site | Retrospective cohort | Multivariate logistic regression, chi-square test, ANOVA, secondary data prediction | Interoperability allowing for rapid and efficient data collection for use in interventions, further studies & analysis. | Interoperability of Epic systems across three locations allowed for large sample size. | Data collected for clinical and billing uses instead of research can lack the temporal relationship to determine disease cause or diagnosis. The three institutions using Epic were in different phases of implementation, so some variables were missing from one site. | None stated. |
|  | Bush 2017 | (25) | USA | CA | Design an algorithm to extract autism spectrum disorder data from the EHR. | HSR | Pediatric | Multi-site | Retrospective cohort | Chi-square test, EHR queries to extract demographics, scheduling, procedures, and prescribed medications for all patient-related encounters. | Importance of using a variety of data extraction methods to capture patients in different clinical workflows. EHR data a rich potential source of data for various forms of research. | Ability to employ queries within a heterogeneous healthcare delivery system - all used same Epic EHR pediatric system. | Challenges encountered included, locating available discrete data elements and missing data. Must query multiple fields to ID cases. | Clarity |
|  | Carter 2017 | (26) | USA | TX | Develop and evaluate a teledermatology store-and-forward system in Epic EHR to improve access to dermatologic care. | HSR | Adult | Multi-site | Prospective | Mann-Whitney test, one-sided proportion test, two-sided Fisher exact test. | Teledermatology reduced median time to evaluation from 70 days to 0.5 days, and median time to treatment from 73.5 days to 3.0 days when compared with in-person dermatology visits. | None stated | None stated | MyChart |
|  | Chak 2022 | (27) | USA | CA | Evaluate an automated, electronic health registry based method for mass chronic hepatitis B screening in an at risk population. | Registry | Adult | Multi-site | Pre-post intervention | Multinomial model | The intervention increased screening by 824% across the 11 primary care clinics. | Used Healthy Planet, a population health module of Epic Systems. | None stated | Healthy Planet |
|  | Chang 2016 | (28) | USA | CA | Describe the implementation outcomes of a EHR-based standardized patient database (Registry for Stones of the Kidney and Ureter). | Registry | Adult | Multi-site | Prospective | Interviews, clinical measures/instruments. | Registry had the ability to collect inpatient and outpatient data in all phases of care. Automatically extracted from the medical record and then organized in a secure online database. | Specific data collection instruments were created within Epic to facilitate automated data extraction. | Automated data extraction requires an upfront investment for programming both structured data capture into clinical notes and data extraction from the EHR. Collaboration between clinicians and analysts needed to optimize quality and appropriate use of data. | None stated. |
|  | Chen 2018 | (29) | USA | CA | Develop a radiation-oncology registry integrated into EHR workflow for use in research and quality assurance. | Registry | Adult and pediatric | Single site | Pre-post intervention | Descriptive statistics | 54 independent research studies used queries supported by the registry. Registry enhanced research efficiency and facilitated quality assurance. | Customized interface between EPIC EMR and the registry - automatic data deposition. | None stated. | None stated. |
|  | Cheriff 2010 | (30) | USA | NY | Evaluate the impact of the implementation of an ambulatory EHR on physician productivity. | HSR | Physicians | Single site | Pre-post intervention | Wilcoxon-signed rank test, multivariate regression | Incremental increase in productivity after six months experience with the EHR. | None stated | None stated | EpicCare |
|  | Chernitsky 2014 | **(31)** | USA | CA | Implement a quality improvement program to improve documentation of quality metrics for rheumatoid arthritis disease management. | QI | Providers and clinic staff | Single site | Pre-post intervention | Descriptive statistics | Project resulted in improvement in all rheumatoid arthritis documentation metrics. | Ability to enhance out of the box solutions to quality metrics in Epic | Many personnel required including IT programmers. | None stated. |
|  | Chiu 2018 | (32) | USA | CT | Association of Lowering Default Pill Counts With Postoperative Opioid Prescribing. | HSR | Adult | Multi-site | Pre-post intervention | Multivariable regression analysis | Lowering the default number of opioid pills is a simple, effective, cheap, and potentially scalable intervention to change prescriber behavior and decrease amounts prescribed. | Changing electronic prescribing defaults helped decrease the mean amount of opioid analgesia prescribed to patients undergoing outpatient procedures by more than 15%. Minimal cost for making the changes | None stated | EpicCare |
|  | Ciricillo 2015 | (33) | USA | OH | Evaluate the frequency and risks associated with clostridium difficile infection (CDI) in the pediatric solid organ transplantation population | Clinical Research | Pediatric | Single site | Retrospective cohort | Bivariate analyses - chi square test & 1-way ANOVA, multiple regression | Clostridium difficile infection occurs in 12% of transplant patients. Age, gender, ethnicity, obesity, recent hospitalization, antibiotic duration/intensity and calcineurin inhibitor choice were not associated. Liver transplant was associated with higher risk of CDI. Acid-blockage was protective. | None stated | None stated | None stated. |
|  | Clendennen 2015 | (34) | USA | TX | Compare risk factors of hospital readmissions between 30-90 day readmissions in low socioeconomic status population. | HSR | Adult | Multi-site | Retrospective cohort | Descriptive statistics, multinomial regression, multiple imputation method for missing data | Days to follow-up and race associated with increased odds of 60 or 90 day readmission. Hispanic patients at greater risk for 30-day readmission. | None stated | None stated | None stated. |
|  | Cox 2018 | (35) | USA | Multistate | Analyze EHR usage by general surgery residents and its association with case volume. | HSR | Physicians | Single site | Retrospective cohort | Binary time series, Fisher's exact test, Wilcoxon rank sum test, longitudinal linear mixed-effect model, Pearson's correlation coefficient. | 30% of general surgery resident time was spent utilizing the EHR. 1/3 of this usage was outside their scheduled shift. No overall correlation between number of operative cases and EHR usage. | Login and logout time data extracted from EPIC. | None stated. | None stated. |
|  | Dang 2016 | (36) | USA | NY | Assess outcomes and treatment plans in patients with HPV-positive head and neck squamous cell carcinoma (HNSCC). | Clinical Research | Adult and pediatric | Single site | Retrospective cohort | Chart review; calculated progression-free survival and overall survival using date of death or last date of follow-up. | 11 patients analyzed, 4 were alive and disease-free with aggressive systemic treatment. | None stated | None stated | None stated. |
|  | De Boer 2016 | (37) | USA | CO | Creation of custom bronchoscopy procedure note to evaluate flexible bronchoscopy use and outcomes | Clinical Research | Physicians | Single site | Prospective | Descriptive statistics, fisher exact test, relative risk ratio, t-test, spearman's correlation | 2.1% of flexible bronchoscopies had unplanned outcomes. No difference in complication rate when procedure performed by trainee or faculty. | Note implemented into EPIC | None stated | None stated. |
|  | DelFiol 2020 | (38) | USA | UT | Develop a clinical decision support to identify and manage patients who meet guidelines for genetic evaluation of familial cancer. | CDS | Adult and pediatric | Single site | Mixed methods | Rule-based algorithm | The CDS platform was successfully piloted and identified a relatively large number of patients who could benefit from genetic evaluation, including individuals who were later found to have pathogenic variants in cancer predisposition genes. | Used an open sourced software that interfaced with EPIC via standard compliant APIs. Has potential to scale and spread. | Relied on often inaccurate or incomplete documentation in Epic. Since EHRs support customization and may use different terms, a term-mapping framework had to be developed for finding data in EHR. | None stated. |
|  | DeLozier 2021 | (39) | USA | TN | Real-time clinical note monitoring to detect conditions for rapid follow-up | CDS | Adult and pediatric | Single site | Prospective | Poisson regression | Event alert system improved recall of the 2 adverse drug events from 43% to 94% compared to the manual approach. | Text match alerts used within EHR. Study took place during a transition to EPIC, and alerts maintained enrollment during this transition. | EPIC BPAs was not used to minimize effort required from providers. | None stated. |
|  | Dhar 2019 | (40) | USA | MI | Determine the frequency of high risk human papillomavirus (HR-HPV) and HPV vaccination in female patients with systemic lupus erythematosus. | Population Health | Adult | Single site | Retrospective cohort | Student t-test, chi-square test, Z-test | HR-HPV infection significantly higher (2 times) in the SLE cohort compared with a national health survey. 4.6% of women with SLE who were eligible for the HPV vaccine received it. | None stated | None stated. | None stated. |
|  | Dillon 2020 | (41) | USA | CA | Determine the personnel costs and revenue generated from a behavioral health nurse practitioner in primary care clinics managing adolescents. | HSR | Adolescent | Multi-site | Cost analysis | Descriptive statistics, cost analysis | Personnel costs were 63% of potential revenue generated, suggesting a primary care behavioral nurse practitioner could be cost-saving. | Found encounters and hours scheduled in Epic | None stated | None stated. |
|  | Dinesh 2021 | (42) | USA | NY | Investigate hospitalizations and outcomes in the public health system during the initital COVID-19 pandemic response. | Population Health | Adult and pediatric | Multi-site | Retrospective cohort | Descriptive statistics, univariate analysis, chi-square test | Higher COVID-19 mortality was associated with increased age, diabetes, hypertension and cardiovascular disease. Variations between hospitals in hospitalizations and outcomes. | None stated | None stated | None stated. |
|  | Dowling 2018 | (43) | USA | IL | Improving lung cancer screening completion rates in a primary care practice in large urban academic medical center | HSR | Adult | Single site | Pre-post intervention | Epic report showed number of BPAs. Process improvement interventions were put in place to alert patients and physicians of need for exams. | BPAs lacked specificity; physicians were skeptical regarding evidence for screening; no time with competing demands | None stated | None stated | None stated. |
|  | Egede 2021 | (44) | USA | WI | Investigate the association between mental and physical health diagnoses and COVID-19 outcomes. | Population Health | Adult | Multi-site | Cross section | Descriptive statistics, logistic regression, cox proportional hazards model, ANOVA, chi-square test, Fisher's exact test | Patients with combined physical and mental health diagnoses had a lower risk of testing positive for COVID-19 but were more likely to be hospitalized compared to individuals with physical health diagnosis alone, but this relationship lost significance after fully adjusting for other factors | None stated | None stated | None stated. |
|  | Eickholtz 2022 | (45) | USA | MI | Investigate the incidence of seizures and continuous EEG abnormalities after TBI (traumatic brain injury) and associated risk factors. | Clinical Research | Adult | Multi-site | Retrospective cohort | Kruskal Wallis test, chi-square test, Fishers exact test. | 12.2% of TBI patients developed seizures within the first week post-injury. 53.3% of seizures were electrographic-only, therefore would have been missed without continuous EEG. | None stated | None stated | None stated. |
|  | Erickson 2021 | (46) | Canada | N/A | Determine the rate at which beta blockers were held in patients with heart failure who were previously on beta blockers, and if there was a difference in blood pressure between days beta blockers were held, or not held. | Clinical Research | Adult and pediatric | Single site | Retrospective cohort | Welch's paired t-test | 23% chance of beta blocker being held for 24 hours. Though the difference in blood pressure and heart rate when beta blockers are held was statistically significant, it was not clinically significant. | None stated | None stated | None stated. |
|  | Escribe 2022 | (47) | USA | MA | Analyze primary care physicians EHR inbox management work. | HSR | Physicians | Single site | Retrospective cohort | Latent Dirichlet allocation model | Study identified themes of inbox messages received by primary care physicians messages - 50.8% medical issues, 34.1% administrative issues, to inform future workflow design and eliminate unnecessary workload. | EHR inbox messages were obtained from Epic. | None stated | None stated. |
|  | Everson 2016 | (48) | USA | MI | Evaluate if electronic health information exchange is associated with improved emergency department care processes and utilization. | HSR | Adult and pediatric | Multi-site | Pre-post intervention | Multivariate analysis, request-to-access time, ordinary least squares regression, meditation analysis. | Health information exchange was associated with faster outside information access which was associated with changes in emergency department care. | Epic Care Everywhere health information exchange platform. | For Epic Care Everywhere, information was returned somewhat less often when compared to phone/fax (72% vs 84%). | Care Everywhere |
|  | Federman 2017 | (49) | USA | NY | Evaluate the impact of an EHR prompt for hepatitis C virus testing in adults born between 1945 and 1965. | CDS | Adult | Multi-site | RCT | Generalized estimating equations, binomial distribution, logit link function, covariance matrix, exploratory analysis. | The EHR BPA increased Hepatitis C virus testing among the cohort, but most eligible patients did not receive testing - a more effective method is needed. | Ability to create specific EHR alert. | None stated | None stated. |
|  | Flatow 2015 | (50) | USA | NY | Evaluate quality of care indicators in the surgical intensive care unit before and after Epic EHR implementation. | QI | Adult and pediatric | Single site | Retrospective cohort | Chi-square test, independent sample t-test | EHR implementation was associated with lower CLABSI (central line associated bloodstream infection) and SICU mortality, however, QI initiatives to reduce CLABSI and mortality in SICU were implemented concurrently. EHR had a role in the QI initiatives. | None stated | None stated | None stated. |
|  | Flatow 2015 | (51) | USA | NY | Trends in ICU quality after implementation of an EHR | HSR | Adult | Single site | Pre-post intervention | Chart review | The EHR had an integral role in ongoing quality improvement endeavors which may explain the changes in central line associated blood stream infection and mortality | None stated | None stated | None stated. |
|  | Gabel 2017 | (52) | USA | CA | Create and validate an algorithm to calculate mechanical ventialtion for cardiac postoperative patients. | Registry | Adult and pediatric | Single site | Prospective | Descriptive statistics, Lin Concordance Correlation Coefficient, Bland-Altman agreement, McNemar test. | Successfully created an algorithm that duplicated the manual process of data extraction and produce higher accuracy results. | Chart review and data abstraction was done using EPIC EHR. | None stated. | None stated. |
|  | Gessner 2023 | (53) | USA | TX | Assess utilization and patient experience of pediatric and adolescent gynecological telehealth visits during the COVID-19 pandemic. | QI | Pediatric & Adolescent | Single site | Cross section | None mentioned | The number of telehealth visits was approximately half the ambulatory volume in the time period assessed. 87.3% of telehealth patients had their expectations met by telehealth services. (Many more findings but cannot fit in 30 words). | Aggregate data was extracted from the EPIC EHR on patient demographics, payer characteristics, and visit type and purpose. EPIC was also used for the telehealth visits. | None stated | None stated. |
|  | Gillen 2022 | (54) | USA | FL | Case presentation of Yamaguchi-variant cardiomyopathy in an athlete. | Clinical Research | Adult | Single site | Case report | None stated. | Study proposes that all athletes receive an ECG and echocardiogram prior to competition, and that AEDs should be common in athletic and other areas. | None stated | None stated | None stated. |
|  | Goehler 2019 | (55) | USA | CT | Develop a CDS algorithm to stratify patient risk and provide recommendations for pulmonary embolism (PE) work-up. | CDS | Adult | Single site | Prospective | Chi-square test, binomial distribution, Forrest plots | CDS algorithm was accurate in identifying patients with PE, but minimally impacted ordering practices. | CDS algorithm integrated into EPIC Radiology Information Ordering System. | None stated. | Radiant |
|  | Goldberg 2016 | (56) | USA | Multistate | Evaluate a clinical decision support tool used in a trial testing brain injury prediction rules for children with minor blunt head trauma. | CDS | Pediatric | Multi-site | RCT | Performance metrics. | CDS system was the source of recommendations in over half of real time cases and nearly all real-time cases. | None stated | Lack of granularity on the EHR clock prevented exploration of the magnitude of execution time differences between the two systems. | None stated. |
|  | Grigoryan 2017 | (57) | USA | TX | Evaluate antibiotic prescribing patterns for uncomplicated acute bronchitis. | Clinical Research | Adult | Multi-site | Retrospective cohort | Chi-square test | Despite guidelines recommending against, antibiotics were commonly prescribed for uncomplicated acute bronchitis, particularly in younger adults. | None stated | None stated | Clarity |
|  | Handley 2022 | (58) | USA | Multistate | Compare length of stay and hospital readmission among postpartum patients before and during the COVID-19 pandemic. | HSR | Adult and pediatric | Multi-site | Retrospective cohort | Standardized difference, Bayesian logistic mixed-effect regression model. | Shorter postpartum hospital length of stay was more common during the COVID-19 pandemic. There was no change in postpartum hospital readmission. | Data pulled from Epic Cosmos research platform, a framework to aggregate EHR data for research, public health, and healthcare operations. | None stated | Cosmos |
|  | Hanna-Attisha 2016 | (59) | USA | MI | Analyze the differences in pediatric blood lead levels after the water source change in Greater Flint, Michigan. | Population Health | Pediatric | Multi-site | Retrospective cohort | Spatial analysis. Interpolation methods (inverse distance weighting and Kriging), chi-square test, ANOVA | Statistically significant increase in the percentage of children with elevated blood lead levels (2.4% to 4.9%) after the water source change. Greater increase in socioeconomically disadvantaged neighborhoods. | None stated | None stated | None stated. |
|  | Harbison 2018 | (60) | USA | PA | Determine how and why women choose prenatal care providers (obstetricians, midwives, or family medicine physicians). | Population Health | Adult and pediatric | Single site | Retrospective cohort | Descriptive statistics | Majority of patients saw an obstetrician. Choices were made based off recommendations and prior usage. | List of patients was obtained from EPIC EMR, these patients were surveyed. | None stated. | None stated. |
|  | Haurani 2020 | (61) | USA | OH | Evaluate time to interpretation, study time, wait time, technologist productivity and critical results reporting in a vascular laboratory after a process improvement strategy. | QI | Not specified | Single site | Retrospective cohort | Time to interpretation analysis, study time analysis, wait time analysis. | Time to interpretation and study time improved after the process improvement strategy, wait time and critical results reporting remained the same. | Most of the data to track time between orders, lab, imaging, results are available in Epic EMR | None stated | None stated. |
|  | Havrilla 2022 | (62) | USA | PA | Development and implementation of PheNominal, a point-of-care web application in EPIC, to assist with capture of standardized phenotype data. | HSR | Pediatric | Single site | Retrospective cohort | None stated. | PheNominal was used to capture phenotype data for 1500 individuals during 16 months. Time to enter HPO (Human Phenotype Ontology) terms on patients was 5 minutes, compared to 15 minutes in manual workflow, with fewer errors. | None stated | None stated | None stated. |
|  | Heidemann 2017 | (63) | USA | MI | To develop an accurate, reliable, and efficient method of identifying patients with bonafide drug induced liver injury (DILI) in an EMR system | Clinical Research | Adult | Single site | Retrospective cohort | Algorithm development for case identification | Text searching method identified more cases than ICD-9 codes | None stated | None stated | EpicCare, ASAP ER Module, Chronicles, Clarity |
|  | Henao 2022 | (64) | USA | Multistate | Determine the difference between feeding tube insertion between White and Black patients with advanced dementia, and evaluate other demographics and clinical characteristics which may contribute. | Clinical Research | Adult | Multi-site | Retrospective cohort | Multivariable logistic regression | Black patients and male patients had higher odds of having feeding tubes inserted than white patients and female patients. Age, etiology, and palliative care consults did not have a statistically significant impact on feeding tube insertion. | None stated | None stated | None stated. |
|  | Hensley 2019 | (65) | USA | MD | Implementation of bedside bar code transfusion verification for intraoperative blood component administration. | QI | Adult and pediatric | Single site | Prospective | Population based auditing | Compliance on patient documentation improved to over 96% after the implementation of bedside bar code transfusion verification and the EPIC anesthesia information management system. | Transition to EPIC anesthesia information management system took place during the QI initiative, after which automated EPIC reports were generated to audit the process. | None stated. | Optime |
|  | Hojat 2020 | (66) | USA | OH | Use an EHR point-of-care alert to promote testing of infants born to mothers with hepatitis C virus. | CDS | Pediatric | Single site | Quasi-experimental | Kaplan-Meier method, log-rank test, interrupted time series analysis. | The alert significantly improved rates and timelessness of hepatitis C virus testing in perinatally exposed pediatrics. | Alert built in Epic | Current alert model could not be applied to regional hospital systems - reason not given. | None stated. |
|  | Holmgren 2022 | (67) | USA | Multistate | Evaluate if there is an association between the time spent in the EHR and the threat of malpractice. | HSR | Physicians and advanced practice practitioners | Multi-site | Retrospective cohort | 2 sided *t* test, ordinary least square regression | No association was found between time spent in the EHR and location in a state in the top-quartile of malpractice payouts. | EHR metadata was from EPIC. | None stated | None stated. |
|  | Horton 2018 | (68) | USA | UT | Assess the impact of a clinical decision support intervention targeting early recognition of sepsis decompensation. | CDS | Adult | Single site | Pre-post intervention | Chi-square test, t-test, descriptive statistics, interrupted time series analysis | Intervention associated with reduced length of stay and total direct cost of hospitalization, without an increase in ICU utilization or broad-spectrum antibiotic use. | None stated | None stated | None stated. |
|  | Howell 2014 | (69) | USA | CA | Evaluate the impact of automated EHR alerts on the compliance of pap test ordering with screening guidelines. | CDS | Adult | Multi-site | Time series | Time series analysis, adjusted relative frequency ratios, multinomial logistic regression, Durbin-Watson statistics. | Interruptive alerts can discourage ordering of Pap tests that do not meet clinical guidelines. | None stated | EHR CDS can result in alert fatigue. CDS tools require continuous IT/EHR support. Provider compliance with alerts is a challenge | None stated. |
|  | Huang 2019 | (70) | USA | MD | Assess rates of drug-induced pruritus. | Clinical Research | Adult | Multi-site | Retrospective cohort | Chi-square test, pairwise student t-tests, Bonferroni correction | Increased rates of drug-induced pruritis found with some antibiotic and cardiovascular drugs. | Data from Epic SlicerDicer | None stated. | SlicerDicer |
|  | Huang 2022 | (71) | USA | TX | Compare the efficacy of clinical breast exams (CBEs) and breast self exams (BSEs). | Clinical Research | Adult | Single site | Retrospective cohort | Fisher's exact test, two-sample t-test | No significant difference was found in the efficacy of CBEs and BSEs, however BSEs identified most cancers and positive ultrasound findings. | None stated | None stated | None stated. |
|  | Isseh 2017 | (72) | USA | OH | Time to procedure: A major factor in deterimning inpatient bowel preparation adequacy | HSR | Adult | Single site | Retrospective cohort | ANOVA, Kruskal-Wallis , Pearson's chisquare, Fisher's Exact test. | More than half of inpatients exposed to single dose evening before bowel preparations have an inadequate bowel preparation. | The use of a structured queried language (SQL) to pull data from Epic. | None stated | None stated. |
|  | Javier-DesLoges 2018 | (73) | France | N/A | To identify risk factors associated with post operative urinary retention | HSR | Adult | Single site | Retrospective cohort | Descriptive analysis, multivariate regression | Age, COPD, stroke, benign prostatic hyperplasia, and longer operative times were associated with greater risk of post operative urinary retention | None stated | None stated | None stated. |
|  | Javier-DesLoges 2019 | (74) | USA | CT | Evaluate the use of a method in Epic EHR to log ureteral stent insertion and removal and decrease lost ureteral stents. and identify patient characteristics influencing risk of failure to follow-up. | QI | Adult | Multi-site | Retrospective cohort | T-test, chi-square test, multivariate logistic regression, binomial regression | 0.9% of patients failed to follow-up for stent explanation. Stents placed emergently and black race were predictors of failure to follow-up. Use of EPIC facilitates easy identification of lost ureteral stents. | EPIC data used to identify ureteral stents and patients at risk for loss to follow-up. | None stated. | None stated. |
|  | Jones 2022 | (75) | USA | CO | Develop and evaluate an EHR tool which measures adenoma detection rate. | CDS | Adult and pediatric | Single site | Prospective | Chart review | Tool correctly identified 76/78 colonoscopies with adenoma and 67/67 colonoscopies with no adenoma. No difference in adenoma detection rate in the tool versus manual chart review. | EPIC functions SmartList and Reporting Workbench used to create adenoma capture tool. | None stated | SmartTools |
|  | Jose 2020 | (76) | USA | MN | Implement a best practice advisory to refer ambulatory oncology patients to tobacco use treatment. | CDS | Adult and pediatric | Single site | Pre-post intervention | Descriptive statistics of pre post analytics of referral rates | BPA did not include a patient decline option, all patients were referred regardless of intent to stop, and it did not require active involvement by clinicians, making it feasible within oncology clinical practices. | Automatic referrals/rooming staff initiating the referral helped with less clinician burnout from alert fatigue. Enabled med support staff to enter data in Epic, not MDs. | Further work needed to address barriers to responding to alerts. | None stated. |
|  | Katzan 2015 | (77) | USA | OH | Describe the implementation and outcomes of an electronic stroke CarePath to manage ischemic stroke patients and improve quality of care. | QI | Adult | Single site | Pre-post intervention | Logistic regression, chi-square test, sensitivity analysis, functional outcome measures, patient-reported outcome measures | Decline in inpatient mortality rates in ischemic stroke patients compared with the controls. Trend towards improved functional outcomes. | CarePath care pathways are integrated into Epic EHR. | Customization of Epic EHR required additional programming resources that were not internally available. | None stated. |
|  | Keizur 2022 | (78) | USA | IL | Evaluate a BPA for its effectiveness in identifying patients who meet the criteria for pre-exposure prophylaxis (PrEP) to prevent HIV. | CDS | Adult and pediatric | Single site | Prospective | None stated. | The BPA was effective at identifying patients, but provider initiation of PrEP or provider acknowledgement of the BPA was low. | BPA initiated in EPIC. | BPA was initiated for any patient encounter, including telephone calls and medication orders, so it may not have always been viewed by a provider. | None stated. |
|  | Kelly 2020 | (79) | USA | WI | Evaluate provider experiences with bedside table inpatient portal app given to hospitalized pediatric patients and their families. | HSR | Providers, pediatric patients & families. | Single site | Cross section | Descriptive statistics, provider survey analysis | 80% of families used the bedside tablet to access the child's health record. Over 90% of providers supported use after implementation. | The availability of patient information at bedside facilitated by Epic MyChart Bedside was beneficial for patients and added no burden to providers. | None stated | MyChart |
|  | Khanna 2021 | (80) | USA | MD | Evaluate the association between COVID-19 infection and demographic and socioeconomic factors. | Population Health | Adult and pediatric | Multi-site | Retrospective cohort | Multivariable binary logistic regression, chi-squared test, latent class analysis | Racial & ethnic minorities had a higher risk of COVID-19 compared with non-Hispanic white and other nonblack racial groups. Age and gender did not have a significant association with COVID-19 infection rate. | None stated | None stated | None stated. |
|  | Kim 2020 | (81) | USA | MI | Determine the relationship between cognitive impairment among Medicare patients and readmissions. | QI | Adult and pediatric | Single site | Ethnographic | Review of processes, equipment, people | MoCA (Montreal cognitive assessment) useful in identifying patients at increased risk of readmission. | None stated | None stated | None stated. |
|  | King 2020 | (82) | USA | PA | Evaluate the cost of robotically assisted gastric bypass surgery vs conventional laparoscopy. | HSR | Adult | Single site | Retrospective cohort | Descriptive statistics, sample t-test, Mann-Whitney rank sum test, chi-square test, Fisher's exact test, comparative statistics. | No statistically significant difference in cost between groups. | None stated | None stated | None stated. |
|  | Klang 2021 | (83) | USA | NY | Use machine learning to predict in-hospital mortality during emergency department triage using a free-text-like method. | CDS | Adult | Single site | Prospective | Machine learning prediction model | AUC of 0.99 for 48 hour mortality and AUC 0.96 for overall in-hospital mortality, however, positive predictive value was low (PPV). | Data from EPIC used to train the models | None stated | None stated. |
|  | Kolb 2016 | (84) | USA | DE | Improving care of patients with sickle cell disease through discrete clinical data extraction and automated analysis of the electronic healthcare records | CDS | Adult and pediatric | Single site | Chart review | Custom flowsheet to capture adverse events and form to track adherence. | Automated reports permit focused efforts targeting adherence to health maintenance needs; Highlights the utility of enhanced granular data entry to inform clinical and research decisions. | Able to add enhanced granular data entry to Epic | None stated | SmartTools |
|  | Koo 2020 | (85) | USA | CA | Increase accuracy of hand off printouts in the pediatric intensive care unit by using handout generated by the EHR. | QI | Pediatric | Single site | Pre-post intervention | Pre-post analysis, surveys | Reduction in printed handoff errors and incompleteness without adding time to handoff procedure. | Only took 6 months to make the changes in Epic EMR; EMR generated tool decreases risk of human error. | EPIC specific changes made, might not be applicable in other EMRs. | SmartTools |
|  | Kukhareva 2022 | (86) | USA | UT | Identify limitations in EHR smoking data and propose a potential approach for addressing these limitations. | Population Health | Adult | Multi-site | Retrospective cohort | McNemar's chi-squared test, Wilcoxon signed rank test, sensitivity analysis, polynomial regression | More than 80% of evaluated records had smoking data inaccuracies. Using longitudinal data to address these issues identified 49.4% more patients potentially eligible for lung cancer screening. | None stated | None stated | None stated. |
|  | Kurian 2014 | (87) | USA | CA | Analyze integrated breast cancer data from three sources - EMRs of two healthcare systems and the state registry. | Registry | Adult | Single site | Retrospective cohort | Algorithm to link records across data sources, chi-square test. | Integrating data from EMRs and population-based registries allowed a more comprehensive understanding of breast cancer care and treatment utilization. | EMRs contain important clinical data not available in other sources. | Unstructured data in clinician notes contains important concepts that could not be extracted, such as nuances of decision making, physician recommendations and patient preferences. | Beacon |
|  | Kurin 2021 | (88) | USA | Multistate | Evaluate the association between irritable bowel syndrome (IBS) and mast cell disorders (MCDs). | Clinical Research | Adult | Multi-site | Retrospective cohort | Bivariate analysis (Pearson chi-square test). | Strong association between IBS and primary and idiopathic mast cell disorders. | Epic SlicerDicer tool (contains de-identified data from EPIC EHR). | None stated | SlicerDicer |
|  | Kuznetsov 2013 | (89) | USA | CA | Use the EHR to identify a real-time cohort of breast cancer patients including treatment process and outcome measures. | Registry | Adult | Multi-site | Prospective | Cost analysis, clinical outcomes analysis | 98% of the EHR cohort matched a tumor registry cohort. Used Epic's BEACON staging module to create sub-cohorts of these patients by stage, and used Beacon to identify chemo regimens. | Epics BEACON staging module facilitated variation analysis across cancer types and stages. | None stated | Beacon |
|  | LaHue 2022 | (90) | USA | CA | Measure the association between COVID-19 severity and the presence of delirium during hospitalization. | Clinical Research | Adult | Single site | Retrospective cohort | Multivariable regressions, Wilcoxon rank-sum tests/t-tests, chi-square tests/Fisher's exact tests. | COVID-19 severity at admission was independently associated with increased risk of delirium during hospitalization. Delirium may be underdiagnosed in COVID-19 patients. | None stated | None stated | Clarity |
|  | Lanzo 2016 | (91) | USA | MD | Increasing patient portal usage: Outcomes from the mychart genius pilot project | QI | Pediatric | Single site | Prospective | Descriptive analysis | MyChart Genius provides youth-friendly tech support during clinical visits. | None stated | Need to enhance technology integration to better individualize communication types for optimal care delivery | MyChart |
|  | Li 2020 | (92) | USA | TN | Evaluate the impact of a CDS tool to optimize perioperative glucose management. | CDS | Adult | Single site | Retrospective cohort | Interrupted time series one-way ANOVA, 3 phases | Intraoperative glucose surveillance rates improved. Decreased rates of postoperative hyperglycemia in at-risk patients. | Outcomes worsened when old system was turned off (no BPAs) and improved when new BPA system was built in Epic | None stated | None stated. |
|  | Lilih 2017 | (93) | Netherlands | N/A | Implement CDS in EPIC and analyze its effect on GI prophylaxis prescription | CDS | Adult and pediatric | Single site | Pre-post intervention | Chi-square test; univariate analysis, logistic regression; descriptive statistics | 78.2% reduction in number of drug safety alerts. GI prophylaxis prescribed more frequently in response to drug safety alert. | Provided access to pharmacy data, as well as alert fatigue data. | None stated | None stated. |
|  | Lindholm 2010 | (94) | USA | WI | Adapt Epic EHR to improve identification and intervention of tobacco users. | CDS | Adult | Multi-site | Pre-post intervention | Descriptive statistics | The EHR modification led to a higher percentage of patients having their tobacco use identified. Pre-implementation data not available on interventions. | High adaptability of Epic EHR. | None stated | None stated. |
|  | Loo 2015 | (95) | USA | CT | Using a European Value-Based Medicine Approach to Evaluate Hepatocellular Carcinoma Care at a US Tertiary Care Center | HSR | Adult | Single site | Retrospective cohort | Chart review | Sicker population with more advanced condition than the Italian cohort. | None stated | None stated | None stated. |
|  | Mahajan 2022 | (96) | USA | VA | Identify patients with incidental pulmonary nodules (IPNs) with an EMR-based protocol. | Population Health | Adult | Single site | Pre-post intervention | Descriptive statistics | 753 patients were identified with true IPNs, lung navigators were able to contact 87% of these individuals, of these, 2% were diagnosed with cancer. | None stated | None stated | None stated. |
|  | Makam 2013 | (97) | USA | TX | Survey the use and satisfaction of primary care providers with performing common tasks in Epic EHR. | HSR | Adult | Multi-site | Cross section | Multivariate logistic regression, descriptive statistics, chi-square test, Hosmer-Lemeshow goodness of fit. | Survey respondents had a high level of use and satisfaction with EHR functions facilitating transactional tasks such as electronic prescribing. | None stated | Variability in use and satisfaction with functions aimed at facilitating medical decision-making, including clinical documentation, health maintenance and preventive screening, problem list updating and electronic messaging. | MyChart |
|  | Manzar 2022 | (98) | USA | LA | Evaluate the social determinants of health (SDoH) of pregnant women using data from EPIC EHR. | Population Health | Adult | Single site | Cross section | None stated. | Lack of physical activity, social support, tobacco use, housing & transport, stress, food insecurity, financial instability were identified as SDoH concerns in pregnant women. | None stated | None stated | None stated. |
|  | Marsolo 2012 | (99) | USA | OH | Describe the implementation of an opt-in biobank using residual clinical samples. | Biobank | Pediatric | Single site | Pre-post intervention | Descriptive statistics | Patient participation higher than anticipated. | Consent in EHR requires no additional research staff and does not significantly impede patient flow. | Consent forms were problematic as Epic can only capture one electronic signature per document. Four different documents needed to be created. Document duplication another issue. | Clarity |
|  | Martin 2017 | (100) | USA | IL | Evaluate treatment outcomes for temporal bone resection and parotidectomy in advanced parotid malignancies. | Clinical Research | Adult | Single site | Retrospective cohort | None mentioned | 60% 2-year survival in patients who underwent parotidectomy with lateral temporal bone resection and neck dissection, despite advanced presentation of parotid malignancy. | None stated | None stated | None stated. |
|  | Mathias 2010 | (101) | USA | IL | Use of EHR data to assess Pap smear overuse in a general internal medicine clinic | HSR | Adult | Single site | Retrospective cohort | Chart review | Secondary EHR data analysis can accurately measure the use of low-value services such as Pap testing. | Possible to use complex algorithms for Epic EHR data to monitor overutilization and qualify overuse. | None stated | None stated. |
|  | McCain 2022 | (102) | USA | GA | Determine if there is a COVID-19 mortality difference between equally insured Black and White patients. | Population Health | Adult and pediatric | Single site | Cross section | Multivariable logistic regression | The difference in mortality between Black and White COVID-9 patients was not statistically significant when controlling for age and insurance type. | None stated | None stated | None stated. |
|  | McCarthy 2021 | (103) | USA | NY | Implement physical activity screening into the EPIC electronic kiosk check-in process and identify factors associated with not meeting physical activity recommendations. | Population Health | Adult | Single site | Cross section | Descriptive statistics, analysis of variance, chi-square test, multivariable logistic regression | 72% of patients completed the physical activity questions. 1/3 of those were reaching recommended levels of physical activity. Female, Black, retired, unemployed, unknown relationship type were associated with not meeting recommended physical activity levels. | Physical activity vital sign embedded into EPIC, | None stated | None stated. |
|  | McDowell 2017 | (104) | USA | MA | Evaluate the effect of Epic EHR implementation on surgical case turnover time. | HSR | Adult | Multi-site | Time series | Two-sample t-test, F-test. | Significant association between implementation of EPIC EHR and decreased operating room efficiency (measured by turnover time). Return to baseline by six months. | EHR may result in more accurate documentation, so values captured may be more indicative of true efficiency data. | Bottlenecks due to computer availability, log-in and software activation delays. Number of charting steps required in new system. | None stated. |
|  | Mehta 2016 | (105) | USA | FL | Evaluate the use of evidence-based problem-oriented templates as a clinical decision support tool on documentation practices. | QI | Physicians | Single site | Retrospective cohort | Unpaired t-test, ANOVA, Kaplan-Meier estimate | Problem-oriented templates were associated with improved quality of documentation without a significant change in total charting time. | None stated | None stated | None stated. |
|  | Mehta 2018 | (106) | USA | MD | Determine the frequency of central serous chorioretinopathy in African Americans/blacks. | Population Health | Adult | Single site | Cross section | Descriptive statistics | Central serous chorioretinopathy may be underestimated in African Americans. | None stated | None stated. | None stated. |
|  | Melnick 2022 | (107) | USA | Multistate | Determine the effect of a user centered CDS on buprenorphine initiation rates in the emergency department. | CDS | Adult | Multi-site | RCT | Wilcoxon signed rank test, generalized estimating equations, binomial family with logistic link, sensitivity analysis | The CDS did not increase patient level rates of buprenorphine initiation in the emergency department. | CDS implemented in EPIC | None stated | None stated. |
|  | Milani 2017 | (108) | USA | LA | Improving hypertension control and patient engagement using digital tools. | Clinical Research | Adult | Single site | Pre-post intervention | Descriptive and logistic regression analysis | Digital health intervention program was effective in activating patients for hypertension control. | None stated | None stated | MyChart |
|  | Milne 2020 | (109) | USA | TX | Implement a CDS tool to improve care of prostate cancer patients at risk for bone complications of androgen deprivation therapy. | CDS | Adult | Single site | Prospective | Descriptive statistics | Referrals for bone testing and other preventive measures improved. | BPA added when test was ordered then "at risk of osteoporosis" added to Epic problem list. | None stated | None stated. |
|  | Mirro 2018 | (110) | USA | Multistate | Examine the long-term impact of wearable cardioverter defibrillators (WCD) on guideline-directed medical therapy (GDMT) in HFrEF patients. | Clinical Research | Adult and pediatric | Single site | Retrospective cohort | Descriptive statistics, Pearson's chi-square test, ANOVA. | WCD use was associated with prescribing adherence to GDMT and recommendations for follow-up echocardiographic studies. | None stated | None stated. | None stated. |
|  | Mitchell 2022 | (111) | USA | MO | Evaluate a CDS alert and reporting tools which identify patients with wild-type transthyretin amyloid cardiomyopathy (ATTR-CM). | CDS | Adult | Multi-site | Phenotyping | Machine learning prediction model | The CDS tool facilitated identification of patients with wild-type ATTR-CM. EPIC Clarity report provided a comprehensive database of ATTR-CM patients. | None stated | None stated | Clarity, Reporting Workbench, SlicerDicer |
|  | Mosk 2017 | (112) | Netherlands | N/A | Assess risk factors for delirium in hip fracture patients, and investigate effects of dementia on delirium | Clinical Research | Adult | Single site | Retrospective cohort | Chi-square test, Fisher's exact test, Kolmogorov-Smirnov, Mann-Whitney U test, Student's t-test, Kruskal-Wallis tests; Kaplan-Meier survival rate and log-rank test. | 35% had delirium during stay and follow-up. Of those with/without dementia, 35%/21% had postop delirium. | None stated | Missing data within EMR. | None stated. |
|  | Mou 2022 | (113) | USA | CA | Creation & evaluation of an emergency general surgery (EGS) patient registry. | Registry | Adult and pediatric | Single site | Prospective | Descriptive statistics | The EHR EGS registry reliably and automatically captured data to support quality improvement and research. | Registry was developed in EPIC EHR. | None stated | Clarity |
|  | Mulhem 2020 | (114) | USA | MI | Evaluate the effectiveless of a hepatitis C screening reminder in EPIC. | CDS | Adult | Multi-site | Retrospective cohort | Analysis of variance test, chi-square test | The hepatitis C screening reminder & corresponding education resulted in a significant increase in the number of hepatitis C screening tests ordered. | None stated | None stated | None stated. |
|  | Munoz 2018 | (115) | USA | CA | Evaluate the effect of a gastrointestinal oncology nurse navigator on quality of care. | QI | Adult | Single site | Prospective | Wilcoxon rank sum test, Pearson's chi-square test, Fisher's exact test, paired t-test | Patients in the oncology nurse navigator program had a significantly shorter time between diagnosis and treatment. No difference found in missed appointment rates. | Study endpoints from EPIC - time from diagnosis to treatment and average number of missed appointments | None stated. | None stated. |
|  | Muqri 2022 | (116) | USA | NY | Patient benefit and cost-effectiveness of a diabetic retinopathy telemedicine screening program in an underserved population. | HSR | Adult | Multi-site | Cross section | Subgroup analysis | The diabetic retinopathy telemedicine screening program was cost effective and has the potential for profitability. Societal benefit of 14.66 quality adjusted life years. | Fundus photography was uploaded to EPIC and remotely interpreted by ophthalmologists. EPIC used for chart review. | None stated | None stated. |
|  | Nagi 2023 | (117) | USA | NY | Investigate psychiatry Consultation-Liaison service referrals to improve the appropriateness and precision of consult requests. | QI | Adult and pediatric | Single site | Cross section | None mentioned | Only 45.8% were valid consults, resulting in inappropriate resource utilization. Details regarding patient demographics and consulting departments were also provided. | Referral report was generated from EPIC, which included details about consults. EPIC was also used for patient demographics. | None stated | None stated. |
|  | Naranjo 2021 | (118) | USA | MN | Determine the safety of ipsilateral IV line placement following breast cancer surgery. | Clinical Research | Adult | Single site | Case control | Continuity adjusted chi-square test | Frequency of complications did not differ significantly between the contralateral arm IV placement group and the ipsilateral arm IV placement group. Not necessary to avoid IV placement in ipsilateral arm post breast cancer surgery. | None stated | None stated | None stated. |
|  | Narayanan 2019 | (119) | USA | IL | Evaluate factors correlating with cognition and depression in epilepsy patients. | Clinical Research | Adult | Single site | Retrospective cohort | Multivariable linear regression, longitudinal analysis | Male gender and lower number of anti-seizure medications were correlated with lower risk of depressive symptoms. | None stated | None stated. | None stated. |
|  | Nasehi 2018 | (120) | USA | OH | Determine the cumulative breast cancer incidence for patients with benign intraductal papillomas on core needle biopsy. | Population Health | Adult | Single site | Retrospective cohort | Descriptive statistics, chi-square test, Fisher exact test, Mann-Whitney *U* test. | Cumulative breast cancer incidence for patients with benign intraductal papillomas was 14% at a median of 9 years. Findings support continued breast cancer surveillance in this population. | Clinical follow-up information obtained from EPIC EMR. | Other data obtained from CoPath Plus (Cerner) clinic anatomic pathology informatic system. | None stated. |
|  | Nellis 2022 | (121) | USA | NC | Evaluate direct operating room supply costs for cardiac surgery using an integrated reporting tool in EPIC. | HSR | Adult and pediatric | Single site | Retrospective cohort | None stated. | Five procedures were identified as accounting for over 50% of the total direct operating room supply costs. | None stated | None stated | None stated. |
|  | Nguyen 2020 | (122) | USA | PA | Examine the impact of a decision support alert with a human-centered design. | CDS | Physicians | Multi-site | Pre-post intervention | Mann-Whitney test, difference-in-differences analysis, paired Wilcoxon signed rank test. | May be a small but modest increase in action on decision support alerts designed with a human-centered approach. | None stated | None stated | None stated. |
|  | Ni 2019 | (123) | USA | IL | Assess the association between antibiotic use in the first year of life and the development of asthma and allergic rhinitis. | Population Health | Pediatric | Single site | Retrospective cohort | Binomial logistic regression model | Significant association found between antibiotic use in the first year of life and beyond, and with development of asthma and allergic rhinitis. | None stated | None stated. | None stated. |
|  | Nikolian 2018 | (124) | USA | MI | Pilot Study to Evaluate the Safety, Feasibility, and Financial Implications of a Postoperative Telemedicine Program. | HSR | Adult | Single site | Prospective | Chi-square test, t test, wilcoxon rank sum tests. | An easy-to-use, secure eClinic platform in Epic facilitated postoperative care for elective and emergent surgery patients; proved to be safe, feasible and sustainable to improve patient satisfaction and increasing hospital revenue. | None stated | None stated | None stated. |
|  | Nikolic 2017 | (125) | USA | OH | Implement CDS to reduce stool culture samples and parasitological exams | CDS | Adult and pediatric | Single site | Pre-post intervention | Descriptive statistics, chi-square test | 54% reduction in exams, 49% reduction in stool cultures. | CDS implemented into EPIC | None stated | None stated. |
|  | Nolan 2020 | (126) | USA | SC | Evaluate the clinical management and risk factors for Trichomonas vaginalis in adolescents. | HSR | Adolescent | Single site | Case control | Descriptive statistics, comparative statistics, univariable logistic regression. | High prevalence of T. vaginalis, gaps in care and inconsistent care. | None stated | None stated | None stated. |
|  | Norton 2016 | (127) | USA | CA | Evaluation of safety and efficacy of nurse practitioner-run outpatient direct current cardioversion program compared to MD-run. | Clinical Research | Adult | Single site | Retrospective cohort | SOFA, 2-way tables, chi-square test, odds and log odds ratios | NP-run DCCV are as safe and effective as MD-run DCCV. LOS shorter in NP versus MD group. Acuity of patients higher in MD group than NP. | Patient identification done through EPIC. | None stated | None stated. |
|  | Noshad 2022 | (128) | USA | CA | Use unsupervised process mining to identify practice patterns in stroke patients. | QI | Adult | Single site | Retrospective cohort | Unsupervised process mining algorithm | The generated process mining maps mimicked the code stroke clinical care pathways. | Data pulled from EPIC event logs. | None stated | None stated. |
|  | Osband 2018 | (129) | USA | RI | Evaluate the benefits of patient portal usage in non-high risk kidney transplant recipients. | HSR | Adult | Single site | Retrospective cohort | Generalized linear model, Kaplan-Meier survival estimation | 35% of patients enrolled in the patient portal. Patient engagement through EHR portal may have greatest utility in moderate risk patients. | None stated | None stated | None stated. |
|  | Osterberg 2017 | (130) | USA | CA | Characterize men who initiated active surveillance for prostate cancer | Clinical Research | Adult | Single site | Retrospective cohort | Descriptive statistics, Kaplan-Meier curve | Serial PSA checks decreased to 20% over 5 years. Initial adherence to PSA and biopsy was high. | None stated | Patient-reported outcomes and social factors not capture in EMR and are underestimated. | CareEverywhere |
|  | Otto 2022 | (131) | USA | PA | Develop and evaluate an automated tool for extracting blood culture and antimicrobial susceptibility results from the EHR. | Clinical Research | Pediatric | Single site | Prospective | Chart review | Tool was 100% accurate when compared with manual chart review. | Tool developed to pull data from EPIC. | None stated | None stated. |
|  | Palestine 2018 | (132) | USA | MD | Assessing the Precision of ICD-10 Codes for Uveitis in 2 EHR Systems | HSR | Not applicable | Single site | Retrospective cohort | ICD-10 coding of uveitis using 2 EHR systems | Substantial disparity in the ICD-10 codes generated for specific uveitides by the 2 EHR systems, implying analysis of large databases generated from the pooling of EHR data could produce biased results. | None stated | None stated | None stated. |
|  | Park 2021 | (133) | USA | NJ | Implement a risk-based HCV screening program for emergency department patients and link identified patients to follow-up care. | CDS | Adult | Single site | Cross section | Chi-square test, logistic regression | The HCV screening program was successfully implemented. 33% of patients with a positive screening test had chronic infection, males > females. In patients born after 1965 with chronic hepatitis C, the prevalence of opioid use was 73.3%. | HCV risk based screening algorithm was implemented in EPIC and triggered an alert. | None stated | None stated. |
|  | Park 2022 | (134) | USA | TX | Find association between flame retardant clothing (FRC) and mycosis fungoides (MF), a type of T-cell lymphoma | Clinical Research | Adult | Single site | Retrospective cohort | Chart review | 8 patients identified with MF who had worn FRC. 3 patients who discontinued use of FRC had almost complete remission of MF. | None stated | None stated | None stated. |
|  | Patil 2022 | (135) | USA | NY | Evaluate the association between herpes zoster virus and COVID-19 vaccination. | Population Health | Adult | Single site | Retrospective cohort | Chi-square test | No statistically significant association found between COVID-19 vaccination and herpes zoster virus. | None stated | None stated | None stated. |
|  | Pho 2019 | (136) | USA | TX | Evaluate how mobile devices have affected oncology patients use of the MyChart electronic health portal. | Clinical Research | Adult | Single site | Cross section | Mann-Whitnet U test, chi-square test, linear Gaussian regression model | Patients with mobile logins to MyChart increased from 4% to 13% over the four year study period. Mobile logins were more frequent among younger, black, and Hispanic patients. | Used EPIC for demographic data, MyChart allows patients to access health information within EPIC. | None stated. | MyChart |
|  | Putka 2009 | (137) | USA | OH | Identify referral, patient follow-through on referral, and factors predicting referral for hepatitis C virus (HCV) patients | Clinical Research | Adult | Single site | Retrospective cohort | Retrospective chart review, descriptive statistics, univariate analysis, multivariate logistic regression. | HCV positive had a 67% referral rate to specialist. Factors contributing to poor referral: test location, specialty referring, RNA status, ALT elevation. Poor attendance predicted by marital/RNA status. | None stated | Psychiatric co-morbidities or substance abuse that would preclude pt from attending referral were "not widely documented". | None stated. |
|  | Raja 2017 | (138) | USA | NY | Develop CDS to recommend return to work decision after lower back pain diagnosis. | CDS | Physicians | Single site | Pre-post intervention | Odds ratio, impact analysis | Physicians more likely to order meds with EHR order set. 10-fold increase in nurse-delivered counseling with EHR intervention. | Interventions done through EPIC. | None stated | SmartTools |
|  | Rajamani 2015 | (139) | USA | MN | Analyze the utilization of clinical decision support tools for immunization and its variability across providers and EHRS. | Population Health | Adult and pediatric | Single site | Prospective | Descriptive statistics, usage patterns | Similar utilization patterns across time periods with the exception of one organization. | High EHR adoption in Minnesota and market dominance of Epic offers an opportunity to promote use of immunization information system through EHRs with better workflow integration. | Provider access and use at point of care is essential to impact immunization services, which may be influenced by organizational processes and technical factors. | None stated. |
|  | Ram 2022 | (140) | United Arab Emirates | N/A | Assess the COVID-19 mortality rate in dialysis patients. | Clinical Research | Adult and pediatric | Single site | Retrospective cohort | Univariate Cox regression analysis | Outcome of COVID-19 infection in hemodialysis patients in Dubai hospital was better in comparison to previously reported studies in other countries. | None stated | None stated | None stated. |
|  | Rameau 2018 | (141) | USA | CA | Implement a difficult airway alert within EPIC EMR to improve access to airway information. | QI | Pediatric | Single site | Prospective | Descriptive statistics, chi-square test | Implementation led to reduction in time required to obtain airway information and resulted in greater provider satisfaction in identify difficult airways and accessing airway information. | None stated | None stated. | None stated. |
|  | Ray 2018 | (142) | USA | MA | Identify clinical decision support malfunctions using statistical anomaly detection models | CDS | Adult and pediatric | Multi-site | Retrospective cohort | Statistical testing of 6 models: Poisson changepoint model, autogressive integrated moving average (ARIMA) model, hierarchical divisive changepoint (HDC) model, Bayesian model, SHESD model, E-Divisive with medican (EDM) model. | Anomaly detection models are useful tools to aid in detection of CDS alert system anomalies. | Retrospective anomaly detection can help find and prevent anomalies. | Anomalies (failure or stoppage of best practice alerts) occur and often go undetected. Offline (retro) detection might be better than detecting online anomalies (in Epic live) where no future data is available. | None stated. |
|  | Read-Brown 2017 | (143) | USA | OR | Examine the time spent by ophthalmologists for EHR use. | HSR | Physicians | Single site | Retrospective cohort | Mixed linear model, aggregate data | Variability in EHR use patterns between ophthalmologists, EHR use takes a significant portion of ophthalmologist time during patient office visits. | None stated | EHR interface decreases efficiency | None stated. |
|  | Redd 2014 | (144) | USA | OR | Evaluate the effect of epic EHR implementation on productivity and efficiency in a pediatric ophthalmology practice. | HSR | Pediatric | Multi-site | Pre-post intervention | Linear regression, unpaired two-tailed t test, descriptive statistics | EHR implementation was associated with lower efficiency and productivity. Overall clinical volume decreased after EHR implementation, half of EHR chart completion occurred outside business hours. Variation between providers. | None stated | Need for improved EHR interface design | None stated. |
|  | Reddy 2021 | (145) | USA | VA | Compare mortality and risk factors among alcoholic cirrhosis patients of two age groups (under 40 & over 40) after their first hospitalization. | Population Health | Adult | Single site | Retrospective cohort | Descriptive statistics, median survival rates, multivariate Cox proportional hazard analysis, MELD score (model for end-stage liver disease), Kaplan-Meier survival curve. | Alcoholic cirrhosis patients under 40 years of age had a five year cumulative mortality of 49.7% after their first hospitalization. Factors associated with increased late mortality include old age, most recent NLR (neutrophils to lymphocytes ratio), hepatic encephalopathy, MELD score on admission. | None stated | No data possible from other facilities "due to logistic reasons", no data on drinking habits or drinking after discharge. Hospitalized patients only no clinic data. | None stated. |
|  | Renjithlal 2022 | (146) | USA | NY | Investigate dual-energy X-ray absorptiometry (DXA) bone mineral density scoring as a predictor of atherosclerotic cardiovascular disease in women. | Population Health | Adult | Multi-site | Retrospective cohort | Logistic regression, descriptive statistics, mixed linear regression | Combined low bone mineral density was independently associated with significant risk of atherosclerotic cardiovascular disease events. | EPIC SlicerDicer used to identify comorbidities and outcomes. | Time to death or ASCVD outcomes were not calculated. | SlicerDicer |
|  | Ritchey 2016 | (147) | USA | OH | Can an EHR order set improve analgesia and sedation in the intubated emergency department patient? | CDS | Adult | Single site | Chart review | Order set creation and use. | EHR order set with higher rates of appropriate analgesia and sedation administration. | Good continued uptake of order set at 6 months | None stated | None stated |
|  | Rizk 2020 | (148) | USA | PA | Evaluate the impact of a dedicated day unit, an approach that fast-tracks patients to an observation unit from an emergency department | HSR | Adult | Single site | Mixed methods | Descriptive statistics, comparative statistics, sample t-tests | Fast-tracking patients through the emergency department to observation unit improved outcomes. | None stated | None stated | None stated |
|  | Rodriguez 2021 | (149) | USA | PA | Determine palliative care consultation rates & influencing factors in patients receiving cytoreductive surgery (CRS) with hyperthermic intraperitoneal chemotherapy (HIPEC). | HSR | Adult | Multi-site | Retrospective cohort | Descriptive statistics, odds ratio, multivariate logistic regression. | 25% were referred for palliative care, compared with national average of 11-16%. Comorbidities and greater age were associated with higher likelihood of referral. | None stated | None stated | None stated |
|  | Rollman 2016 | (150) | USA | PA | Examine effectiveness of combining internet support groups with CBT vs CBT alone for treating depression and anxiety. | Clinical Research | Adult | Multi-site | RCT | t-test, linear mixed models, poisson regression, kaplan-meier | Significant favoring of CCBT+ISG for 60-75 yo patients; similar improvements for CCBT+ISG and CCBT alone groups. CCBT alone significant improvement compared to usual care arm. | EMR-generated prompt for patient identification through EPIC | Proper identification dependent on diagnostic codes in EMR. | None stated |
|  | Rose 2018 | (151) | USA | CA | Population-based analysis of an EHR-embedded decision support tool for left atrial appendage closure device in patients with atrial fibrillation | CDS | Adult | Multi-site | Retrospective cohort | Patient level analyses were performed to compare treatment strategies. | Ability to embed a decision support tool with decision analytic computational engine into Epic to assist clinicians and patients with decisions about thrombolytics | None stated | None stated | None stated |
|  | Rose 2022 | (152) | USA | CA | Investigate if contextual factors in EHR audit log data can explain variations in time to deliver tPA to patients with acute ischemic stroke (door to needle time). | Clinical Research | Adult | Multi-site | Retrospective cohort | Descriptive statistics, multivariate model, bivariate model | Prior team experience was associated with shorter door to needle time, team busyness was not consistently associated with door to needle time. | Used EPIC audit log data which tracks EHR user-interactions. | None stated | None stated |
|  | Rove 2018 | (153) | USA | CO | Understand factors surrounding unplanned inpatient consultations of pediatric urologists and interventions after consultation. | HSR | Physicians | Single site | Prospective | Two-sided Student's t-test, Fisher's exact test, chi-square test, linear regression, bivariate logistic regression, multivariate logistic regression | 36% of consults required an intervention, factors associated with intervention included consultation from the emergency department, relevant radiologic or laboratory findings, and consultation after hours. | Consults tracked using a consult note template with embedded data collection fields directly within EPIC. | None stated. | None stated |
|  | Ruan 2022 | (154) | USA | NY | Analyze the effect of telemedicine implementation during COVID-19 on volume, physician efficiency, and burden. | HSR | Physicians | Single site | Retrospective cohort | Mann-Whitney U test, linear regression, Wilcoxon signed rank test, Kruskal-Wallis test/analysis of variance, Spearman rank correlation coefficient. | At the start of the pandemic, physicians had fewer appointments, higher same day closure rates, and spent less time writing notes in the EHR outside of working hours. Male physicians had spent less time in the EHR outside working hours and had better efficiency measures. | Metrics from EPIC Signal | None stated | Signal |
|  | Ryan 2021 | (155) | USA | CO | Compare the performance of three predictive models of readmission: LACE, LACE+, LACE-SDH (social determinants of health) and Epic, in an urban safety-net population. | CDS | Adult | Multi-site | Retrospective cohort | Logistic regression, predictive modelling | Epic model had the best performance, LACE-SDH (social determinants of health) performed significantly better than LACE and LACE+ in this population. | None stated | Epic model for readmission prediction is limited to users of Epic EMR system | None stated |
|  | Sahu 2022 | (156) | USA | NJ | Assess continuity of care of patients receiving buprenorphine treatment via tele-health versus in-person visits. | HSR | Adult and pediatric | Multi-site | Pre-post intervention | Chi-square test | Continuity of care was higher for patients using telehealth. | None stated | None stated | None stated |
|  | Salem 2023 | (157) | USA | PA | Compare the direct medical costs of robotic sleeve gastrectomy (R-SG) to a standard laparoscopic approach (L-SG). | HSR | Adult | Single site | Retrospective cohort | Student's *t* test, Mann-Whitney rank sums test, chi-square test. | Direct medical cost of R-SG was slightly lower than L-SG, but the difference was not statistically significant. When patients who also underwent a paraesophageal hernia repair were excluded, the direct medical cost of the R-SG was significantly lower than L-SG. | Direct cost data was from StrataJazz reporting module which receives data from EPIC. | None stated | None stated |
|  | Sawalha 2021 | (158) | USA | AR | Case series of two patients with COVID-19 induced sinus bradycardia treated with theophylline. | Clinical Research | Adult | Single site | Case report | Chart review | Theophylline was used after atropine to restore sinus rhythm in the two cases. | None stated | None stated | None stated |
|  | Schleelein 2016 | (159) | USA | PA | Determine perioperative adverse event prevalence and case characteristics | Clinical Research | Pediatric | Single site | Case control | Summary statistics, Wilcoxon-rank sum test, fisher-s and chi-squared tests, univariate and multivariate log reg, ORs. | Age, comorbidity, multiple surgical services, location, and provider experience were risk factors for perioperative adverse events requiring rapid response. | None stated | None stated | None stated |
|  | Serna 2022 | (160) | USA | MA | Determine if EPIC readmission risk score is a predictor of post discharge 6-month mortality, and if it is a potential identifier of patients who may benefit from serious illness conversation (SIC). | HSR | Adult | Multi-site | Retrospective cohort | Reciever operating characteristic (ROC) curve, AUROC, multivariable logistic regression | Epic RRS was predictive of post-discharge 6 month mortality, and was significantly associated with serious illness conversation documentation. | EPIC Readmission Risk Score | None stated | None stated |
|  | Shelden 2021 | (161) | USA | MI | Integrate and evaluate an IV insulin calculator in Epic EHR in the cardiovascular intensive care unit (CVICU). | CDS | Adult and pediatric | Single site | Pre-post intervention | Nonparametric Wilcoxon rank-sum test | Overall the implementation led to a trend towards less hypoglycemia/hyperglycemia and increased frequency of blood sugar at various glycemic targets. | The IV insulin calculator used functionalities available within EPIC, preventing the need for purchasing a third-party software, the cost of which was previously prohibitive. | None stated | None stated |
|  | Shelley 2017 | (162) | USA | NY | Assess the impact of EHR intervention on tobacco treatment performance measures. | CDS | Adult | Multi-site | RCT | Chi-square test. | Smart phrase used more in intervention group. Difficult to modify physician behaviour, but possible with EMR integrated learning and decision making tools. | CDS implemented into EPIC. | None stated | None stated |
|  | Siegfried 2015 | (163) | USA | MO | Evaluate confirmed eczema herpeticum ADEH+/- patients who received intereron gamma for eczema treatment | Clinical Research | Pediatric | Single site | Retrospective cohort | Descriptive statistics | ADEH + patients have higher presence of microbiologic markers. Interferon gamma did not result in dramatic improvement. | None stated | None stated | None stated |
|  | Siff 2016 | (164) | USA | OH | Design an EMR-assisted intervention to reduce rate of patients discharged from ED with abnormal vital signs. | CDS | Adult and pediatric | Single site | Pre-post intervention | Likelihood ratio chi-square | BPA successful in reducing clinically relevant abnormal vital signs at discharge from ED. | BPA integrated into EPIC. | None stated | None stated |
|  | Simmons 2022 | (165) | USA | Multistate | Investigate the association between eosinophilic esophagitis (EoE) and chronic rhinosinusitis (CRS). | Population Health | Adult and pediatric | Multi-site | Cross section | One-way analysis of variance | 8 times greater prevalence of concurrent EoE in patients with CRS. | Quantitative data from EPIC - SlicerDicer | SlicerDicer prevented assessment of temporality - cannot determine the timeframe between isolated and concurrent disease. Contributing comorbidities and disease burden could not be assessed given limited ability to search individual patient history. | SlicerDicer |
|  | Simon 2023 | (166) | USA | OH | Identify patients with a positive influenza test for emergency department (ED) visits within the prior 14 days, indicating a missed opportunity for influenza vaccinations, and evaluate their subsequent healthcare resource utilization. | HSR | Adult and pediatric | Multi-site | Cross section | Chi-square test, Fisher's exact test, t-test | 21.3% of patients with a positive influenza test in the ED had a missed opportunity to be vaccinated for influenza. Of those, 14.4% had influenza related ED visits and inpatient admissions. | None stated | None stated | None stated. |
|  | Simon 2023 | (167) | USA | TX | Assess the utility of a BPA in EPIC to recruit pediatric patients with autism spectrum disorder (ASD) for the SPARK study, the largest study in the USA on ASD. | CDS | Pediatric | Multi-site | Prospective | Fisher's exact test | Provider action was recorded in 51.2% cases of the BPA firing. 64.0% of patients were interested in participation in the SPARK study, and 20% of those enrolled. The BPA contributed to 23.0% of all SPARK enrollments in a 12 month period. | BPA was implemented in EPIC. | Alert fatigue in EHR may cause some providers to dismiss the BPA. | None stated. |
|  | Snyder 2021 | (168) | USA | CA | Compare the effectiveness and tolerability of intramuscular chlorpromazine versus intramuscular olanzapine for aggression in adolescent psychiatric patients. | Clinical Research | Adolescent | Single site | Retrospective cohort | Descriptive statistics, chi-square test, analysis of variance, regression analysis | Both intramuscular chlorpromazine and olanzapine were effective and tolerable. Olanzapine showed greater change in BARS score (behavioral activity rating scale), chlorpromazine had higher likelihood of returning patients to baseline. | None stated | None stated | None stated. |
|  | Sonstein 2014 | (169) | USA | TX | Implement order set into EHR to reduce variation in steroid administration in COPD patients | CDS | Adult and pediatric | Single site | Pre-post intervention | T-test, chi-squared test, wilcoxon test, F test | Standardized care using BPA reduces variation in care. Post group had 49% decrease in steroid use. No change in LOS or mortality; post had higher rates of follow-up appts booked at time of discharge. | Created, validated and implemented BPA using EPIC. | None stated | None stated. |
|  | Sroujieh 2016 | (170) | USA | OH | Using EHR (EHR) beset practice alert (BPA) to improve RBC transfusion practices and adherence to the guidelines | CDS | Adult | Single site | Pre-post intervention | BPA creation and use | BPA helpful as a guide and a reminder to providers about the most recent evidence based guidelines. | None stated | None stated | None stated. |
|  | Stapel 2022 | (171) | USA | MS | Investigate if patients taking ET-1 receptor antagonists (ERAs) have improved glycemic control. | Clinical Research | Adult | Multi-site | Case control | Nonparametric Mann-Whitney test, Willcoxon t-test, Fisher's exact test, multivariate linear regression | Patients in the ERA group had a HbA1c significantly decreased from baseline, where patients not prescribed an ERA had a baseline increase in HbA1c. No statistically significant decreases in weight/BMI in either group. | Patient Cohort Explorer is a clinical data bank containing EHR records deidentified and extracted from EPIC, for 1 million patients at the University of Mississippi Medical Center. | None stated | None stated. |
|  | Straub 2013 | (172) | USA | IL | Validate novel EHR case-finding algorithm to facilitate preconception care | CDS | Adult and pediatric | Multi-site | Cross section | Analytic methods not described; survey of random sample conducted to validate algorithm cases | automated algorithm via EHR to identify at-risk women is feasible and can inform pre-conception patient education | None stated | Absent EHR documentation lead to poor algorithm results on certain risk factors. | Clarity |
|  | Stutz 2018 | (173) | USA | CA | Clinical decision support to improve of end-of-life planning at inpatient discharge | CDS | Adult and pediatric | Single site | Pre-post intervention | Descriptive statistics, Fisher's exact test | Within EPIC, they developed logic to provide a passive text prompt within the discharge navigator for providers to complete a POLST form if the patient was DNR. | None stated | None stated | None stated. |
|  | Su 2018 | (174) | USA | CT | Examine post-operative opioid prescribing patterns following urologic surgery. | HSR | Adult | Multi-site | Retrospective cohort | Multivariable logistic regression | Significant variation in opioid prescribing practices following urologic surgery. Psychiatric diagnosis was a factor associated with higher opioid doses. Optimizing post-operative opioid estimates necessary to improve safety. | Ability to view and compare data across multiple sites. | None stated | None stated. |
|  | Ter-Minassian 2019 | (175) | USA | MD | Evaluate quality metrics used for adults with sickle cell disease at John Hopkins and how they may apply to Kaiser Permanente Mid-Atlantic states patients. | QI | Adult | Multi-site | Cross section | T-test, chi-square test, multivariate logistic regression, binomial regression | Interventions to improve adherence to quality metrics are necessary in both health systems. | Demographics, genotype, and data on quality metrics were collected from EPIC. | None stated. | None stated. |
|  | Tham 2016 | (176) | USA | Multistate | Describe the implementation of traumatic brain injury prediction tools as an EHR clinical decision support. | CDS | Pediatric | Multi-site | RCT | Sociotechnical analysis, structured data collection from EHR. | The CDS system in Epic EHR supported a multicenter clinical trial for traumatic brain injury in pediatrics. | Allowed for a centralized CDS build. Data was extracted from nightly EHR data in | Customized workflows led to varying completion rates across sites as well as differences in the types of providers completing the electronic data form; Challenging to maintain the same rigor across sites. | None stated. |
|  | Tham 2016 | (177) | USA | Multistate | Detail methods and lessons learned to build and implement CDS in 10 emergency departments | CDS | Adult and pediatric | Multi-site | RCT | No analysis reported | Each site customized own workflow, leading to varying completion rates and differing rigor across sites. Centralized build and export of CDS into multiple sites was successful. | CDS implemented into EPIC. | None stated | None stated. |
|  | Torres 2023 | (178) | USA | Multistate | Examine the associations between race, ethnicitiy, language, social risk screening, and patient reported social risks. | Population Health | Adult | Multi-site | Cross section | Multivariable logistic regression | 30% of health centers conducted social risk screening. 11% of patients in the study population were screened for social risks in the analysis period. Screening varied significantly by race/ethnicity/language. | None stated | None stated | None stated. |
|  | Toscos 2020 | (179) | USA | IN | Examine patients’ experiences interacting with their remote monitoring data through digital dashboard as part of daily life. | CDS | Adult | Single site | RCT | Descriptive statistics, paired T-tests, qualitative description | Interacting with remote monitoring data was associated with more patient engagement with their data/dashboard, no usability issues | Built a new dashboard in MyChart which increased engagement with their numbers and linkage to symptoms and activities. Well received | 5 declined looking at it over time with no change in values (stable). Required more time of clinicians to educate patients on what they were seeing. | MyChart |
|  | Toscos 2020 | (180) | USA | IN | Improve disease knowledge and medication adherence among a sample of AF patients using tailored education and nudges in MyChart | CDS | Adult | Single site | RCT | Descriptive statistics, generalized linear modelling to predict adherence vs actual adherence | Tailored educational and reminder messages contributed to increased adherence and disease knowledge among AF patients, though certain patient characteristics moderated the intervention’s effectiveness | Improved knowledge level and adherence can be achieved with nudges and education in MyChart. | Even though it was an RCT, Still hard to isolate the effects of the education and reminders vs other variables (e.g. length of time with diagnosis) | MyChart |
|  | Tsai 2015 | (181) | USA | IL | Electronic best practice advisories' effectiveness in detecting sepsis in the emergency department | CDS | Not specified | Single site | Retrospective cohort | Chart review | BPAs were an effective EHR-based tool that detected potentially septic patients with moderate sensitivity and high specificity. | None stated | None stated | None stated. |
|  | Unni 2015 | (182) | USA | UT | Estimate fracture risk using variables in EMR from FRAX risk calculator | Clinical Research | Adult | Single site | Retrospective cohort | Descriptive statistics, sensitivity analyses. | Low frequency in EMR of risk factors used to calculate risk estimate. Therefore, 10-year absolute fracture risk and treatment-eligible patient proportion are underestimated. | None stated | EMR had missing observations, only one of the clinics recorded data for all of the risk factors. | None stated. |
|  | Van den Broek 2021 | (183) | Netherlands | N/A | Investigated the feasibility of mandatory documentation of the indication for all hospital antibiotic prescriptions. | QI | Adult | Multi-site | Prospective | Descriptive statistics | Mandatory documentation was reliable and time-efficient. Considerable variation in guideline adherence across the three hospitals for community acquired pneumonia, complicated UTI and cystitis. | Since EPIC EMR can be personalized for the needs of a facility, specification of extracted data is necessary before comparisons can be made with datasets of other hospitals. | None stated | None stated. |
|  | Vemulakonda 2017 | (184) | USA | Multistate | Assess adherence to recommendations for annual follow up of children with vesicoureteral reflux. | Clinical Research | Adult and pediatric | Multi-site | Retrospective cohort | Chi-square test, univariate analyses and logistic regression models for multivariable analyses | Follow-up adherence associated with site of care, age, initial evaluation, and treatment plan. | None stated | None stated | None stated. |
|  | Vemulakonda 2022 | (185) | USA | Multistate | Assess adherence to American Urological Association recommendations for annual followup in patients with vesicoureteral reflux. | Clinical Research | Pediatric | Multi-site | Retrospective cohort | Kruskal-Wallis test, chi-square test, logistic regression | Overall adherence to guidelines for annual follow-up is low (less than 25% of cohort). Practice level variations in adherence found. | None stated | None stated | None stated. |
|  | Vesco 2016 | (186) | USA | OR | Health system-based intervention to improve diagnosis and treatment of genitourinary syndrome of menopause (GSM) | Clinical Research | Adult and pediatric | Multi-site | RCT | Retrospective chart review. | Clinicians will use electronic charting tools for GSM, but diagnosis of GSM only significantly increased in gynecologists. | Tools available in EPIC for diagnosis, treatment, patient education of GSM. | None stated | SmartTools |
|  | Vlashyn 2023 | (187) | USA | OH | Assess the benefit of using the iVent tracking tool, which is embedded in the EPIC EHR, to track pharmacist interventions in the ICU, compared to progress note documentation alone. | HSR | Adult | Single site | Prospective | One-sample chi square test, Kruskal-Wallis *H* test, Dunn's procedure | The iVent tool increased the capture of pharmacist interventions by 563% when compared with progress note documentation alone. 84.9% of pharmacist interventions were captured using the iVent tool. | iVent is a tracking tool embedded in the EPIC EHR. | None stated | None stated. |
|  | Wagner 2015 | (188) | USA | IL | Integrate patient reported outcome assessment into the EHR. | HSR | Adult | Single site | Quasi-experimental | Patient Reported Outcomes Measurement Information System (PROMIS), computer adaptive tests (CATs), item response theory (IRT). | 79% of MyChart messages sent were reviewed by patients, the patient reported outcome assessment was started by 37% of patients, and 93% of those completed it. Lower level of physical function and elevated anxiety found when compared to the general population. | None stated | None stated | MyChart |
|  | Wagner 2022 | (189) | USA | CT | Develop a process in EPIC EHR to track indewelling ureteral stents and determine the incidence of forgotten stents. | QI | Pediatric | Single site | Prospective | Descriptive statistics | The automated EPIC report could identify patients who had ureteral stents placed. No forgotten stents were found. | Stent tracking implemented in EPIC. | None stated | None stated. |
|  | Walter 2021 | (190) | USA | WV | Identify the prevalence of obesity and comorbidities in rural pediatric population with migraines. | Population Health | Pediatric | Single site | Cross section | Descriptive statistics, chi-square test, student's *t*-test. | Almost half of the pediatric patients with migraines were overweight or obese, and 26% were obese. No significant differences in age, gender, or race between patients with migraines classified as obese or not obese. | None stated | None stated | Clarity |
|  | Wamsley 2014 | (191) | USA | CA | Use EHR tools to facilitate alcohol assessment, intervention, and related skills in internal medicine residents. | QI | Physicians | Single site | Prospective | Descriptive statistics, chart review | Few residents used the EHR tools - must be better integrated into workflow to be useful. Screening, brief intervention, and referral to treatment skills were inconsistently applied in practice. | Specific alcohol documentation tools within Epic | Adoption of EHR tools in clinical practice was low - tool had to be initiated by user. Integration into workflow with active prompts requires EHR customization. | None stated. |
|  | Wang 2019 | (192) | USA | CA | Evaluate EHR usage of residents across inpatient medicine rotations. | HSR | Physicians | Single site | Retrospective cohort | Descriptive statistics, Welch's t-test | Night shift activity in EHR stopped at end of shift, where day shift activity in EHR continued post-shift. 1st and 2nd year residents spent 2.4-4.1 times more time on information review than information entry. | Event log data extracted from EPIC. | None stated. | None stated. |
|  | Wendel 2023 | (193) | USA | CO | To develop and analyze the utilization of COVID-19 clinical pathways embedded in the EPIC EHR. | CDS | Adult and pediatric | Multi-site | Prospective | None stated | 69 facility-specific pathways were created. Between March 14 to December 31, 2020, the pathways were opened and viewed 21,099 times. 81% of utilization was in the emergency department. | None stated | None stated | None stated. |
|  | Weng 2021 | (194) | USA | TX | Develop and evaluate the results of an optimized workflow in a high-volume radiation oncology department. | QI | Adult and pediatric | Single site | Pre-post intervention | Patient flow analysis | Implementation resulted in significantly shorter patient wait times and shorter consult duration. | Used Epic status board to collect timepoints, updated by clinical staff. | None stated | None stated. |
|  | Williams 2019 | (195) | USA | CO | Describe the clinical reasons for anti-infective use in an inpatient setting. | Clinical Research | Pediatric | Single site | Retrospective cohort | Descriptive statistics | Most common indication was medical/surgical prophylaxis, followed by sepsis/bacteremia and pneumonia/sinusitis. | Orders, drug information, and provider-selected order indications extracted from Epic. | None stated. | None stated. |
|  | Willis 2022 | (196) | USA | Multistate | Use the EHR to identify patients with heart failure at risk for having wild-type transthyretin amyloid cardiomyopathy (ATTRwt-CM). | CDS | Adult | Multi-site | Phenotyping | Random Forest Model, quantitative comparative analysis | CDS showed potential usefulness in identifying patients at risk for ATTRwt-CM. | EPIC was one of three EHRs the CDs was implemented within. EPIC Clarity report comprehensive & allowed for longest search period and most diagnosis data sources. | SlicerDicer allowed only one phenotype combination to be implemented at a time, resulting in greater manual workload. SlicerDicer presents population data, not patient level data. | Clarity, SlicerDicer |
|  | Winden 2014 | (197) | USA | MN | Evaluation of health information exchange tool Care Everywhere and its impact on patient care | HSR | Physicians, staff, and patients | Multi-site | Cross section | Qualitative analysis of focus group; Inter-observer agreement for chart review | Point-to-point HIE reduces the amount of diagnostic testing needed in ER by helping staff provide more efficient care. | EHR ED clinical notes extracted from EPIC, tool implemented in EPIC | None stated | None stated |
|  | Wu 2014 | (198) | USA | PA | Describe the use of Epic EHR implementation to improve the pre-transplantation process for kidney and pancreas transplants. | QI | Adult and pediatric | Single site | Retrospective cohort | Descriptive statistics, time analysis | Decrease in referral to evaluation and evaluation to listing times. Zero-preventable errors occurred. Increases in new patients and number of living and deceased donor kidney transplants. | Epic used to improve referral and evaluation processes, initiate new safety checks and record keeping tools. Use of electronic templates and tracking within Epic. | None stated | Care Everywhere |
|  | Wu 2020 | (199) | USA | CT | Investigate viral interference between rhinovirus and IAV with use of clinical data and an experimental model. | Clinical Research | Adult | Single site | Retrospective cohort | Descriptive statistics. x2, Fisher's exact tests, and odds ratios | Infection with one virus can make a host less susceptible to another virus and interrupt a cycle that can lead to an epidemic | None stated | None stated. | None stated. |
|  | Wyatt 2021 | (200) | USA | NY | Develop a phenotyping algorithm to identify patients with diagnosed-but-untreated chronic hepatitis C, link patients to care, and identify barriers to treatment. | QI | Adult and pediatric | Multi-site | Phenotyping | Phenotyping algorithm, chart review, multivariable logistic regression, bivariate logistic regression, weighted one-sample chi-square test, two sample *t* test. | Compared with manual chart review, the phenotyping algorithm had high PPV and NPV and could risk stratify the cases found. | Algorithm is designed to accommodate diverse data formats, therefore is portable to other EPIC EMRs and potentially non-Epic EMRs. | Informatics experts are required to apply the algorithm. Incomplete EMRs limits data quality | None stated. |
|  | Yazdanshenas 2022 | (201) | USA | CA | Evaluate the safety and effectiveness of the DTRAX facet system in treating cervical radiculopathy. | Clinical Research | Adult | Single site | Case report | Descriptive statistics | DTRAX facet system was found to be safe and effective. No differences in outcomes between single and multi-level DTRAX operations. Small cohort (14 patients). | Used surgical cases report function of EPIC EHR | None stated | None stated. |
|  | Zazove 2017 | (202) | USA | MI | Explore how clinicians perceive and use BPAs & design a more effective BPA for hearing loss screening based on that information. | HSR | Adult | Multi-site | Cognitive task analysis | Qualitative methods, cognitive task analysis. | Six-fold increase in hearing loss detection with the BPA. Issues included: Poor standardization/formatting, time pressure, clinic workflow variations, mental model of the condition being prompted, Epic barriers/health system & government regulations. | None stated | Rigidity of Epic - difficult or impossible to implement changes such as font, color, and word placement that would help with usability. Functioning not intuitive - too many fields to fill out that are irrelevant to the disease. BPAs presented in different formats, leading to more time to fill each one out. Too many clicks. | None stated. |
|  | Zetumer 2018 | (203) | USA | CA | Evaluate the accuracy of data automatically extracted from the EHR compared to manually extracted data. | Registry | Not specified | Single site | Retrospective cohort | Logistic regression | Discrepancy rate between automatically and manually extracted data was 4%. Automatic extraction of data from EHRs possible. | None stated | Reducing free response entry should be considered in EHR - had 2.1x chance of being discrepant compared with non free text data. | None stated. |
|  | Zhang 2021 | (204) | USA | PA | Determine the safety of laser treatments for burn scars. | Clinical Research | Adult and pediatric | Single site | Cross section | Descriptive statistics, t-test, mixed-effect logistic regression, chart review. | Minimal complications or adverse effects found, laser treatments for burn scars were found to be safe and effective. | None stated | None stated | None stated. |
|  | Zhou 2020 | (205) | USA | OH | Evaluate if machine learning algorithms can be implemented to predict CTRCDs in cancer patients according to clinically relevant variables | Clinical Research | Adult | Single site | Retrospective cohort | Descriptive stats and predictive model development and testing | Model inspection revealed variables significantly associated with CTRCDs, including age, hypertension, glucose levels, left ventricular ejection fraction, creatinine, and aspartate aminotransferase levels | None stated | None stated. | None stated. |
|  | Zorn 2022 | (206) | USA | AR | Implement and evaluate a BPA to increase referrals for cancer genetic counseling and testing. | CDS | Adult and pediatric | Single site | Prospective | Descriptive statistics | BPA was completed for 14.9% of patients in 7.2% of encounters. Most common response to BPA was dismissal due to: lack of belief it was a clinical priority, slowing workflow, competition with other BPAs. | None stated | EPIC could not gather the complex clinical data needed for the referral decision automatically (such as if breast cancer has triple-negative histology), so a questionnaire needed to be used. | None stated. |

# **References**

1. Adler D, Abar B, Wood N, Bonham A. Interventions to Improve Adherence to Cervical Cancer Screening Recommendations among Emergency Department Patients: enrollment Data in Anticipation of Interventional Trial Results. 2019;57(3):423‐4.

2. Alkilany R, Einstadter D, Antonelli M. Urate-lowering therapy for patients with gout on hemodialysis. International journal of rheumatic diseases. 2022;25(7):769-74.

3. Altice N, Gerow R. Early Cardiac Rehab to Reduce Heart Failure Readmissions. Heart & Lung. 2020;49(2):211-.

4. Alzahri MS. The Utility of Serum Creatinine Kinase in Emergency Department Patients with Possible Substance-use Related Conditions. The western journal of emergency medicine. 2020;21(5):1195-200.

5. Amaratunga EA, Corwin DS, Moran L, Snyder R. Bradycardia in Patients With COVID-19: A Calm Before the Storm? Cureus. 2020;12(6):e8599.

6. Andersen RL, Ibarra J, Davis T, Estrella L, Andersen L, Oluleye M. Familial hypercholesterolemia (FH) community initiative using electronic health records (EHR). Journal of Clinical Lipidology. 2015;9(3):451.

7. Aslam R, Tabbaa H, Yarbrough A. A retrospective evaluation of compliance to clostridium difficile treatment guidelines. 2019;114:S135.

8. Austrian JS, Volpicelli F, Jones S, Bernstein MA, Padikkala J, Bagheri A, et al. The financial and clinical impact of an electronic health record integrated pathway in elective colon surgery. Applied Clinical Informatics. 2020;11(01):095-103.

9. Bajracharya A, Gerber B, Amante DJ. Id plus care: nudging patients towards guidelineconcordant diabetes care. 2021;36(SUPPL 1):S9‐S10.

10. Barclay C, Viswanathan M, Ratner S, Tompkins J, Jonas DE. Implementing Evidence-Based Screening and Counseling for Unhealthy Alcohol Use with Epic-Based Electronic Health Record Tools. Joint Commission journal on quality and patient safety. 2019;45(8):566-74.

11. Beck JD, Deegan JH, Riehl JT, Klena JC. Incidence of scapholunate ligament dissociation in patients with aspiration-confirmed gout. Journal of Hand Surgery. 2010;35(12):1938-42.

12. Behnke AJ, Tershak DR, Abel WF, Bankole AA. A Therapeutic Approach to Primary Hyperparathyroidism: A Collaborative Evaluation Between Endocrinology and Surgery. Cureus. 2023;15(1):e34157.

13. Beiser M, Lu V, Paul S, Ni J, Nazar N, Epstein E, et al. Electronic Health Record Usage Patterns: Assessing Telemedicine's Impact on the Provider Experience during the COVID-19 Pandemic. Telemedicine and e-Health. 2021;27(8):934-8.

14. Belli HM, Chokshi SK, Hegde R, Troxel AB, Blecker S, Testa PA, et al. Implementation of a Behavioral Economics Electronic Health Record (BE-EHR) Module to Reduce Overtreatment of Diabetes in Older Adults. Journal of general internal medicine. 2020;35(11):3254-61.

15. Bellon JE, Stevans JM, Cohen SM, James AE, Reynolds B, Zhang Y. Comparing Advanced Practice Providers and Physicians as Providers of e-Visits. Telemedicine journal and e-health : the official journal of the American Telemedicine Association. 2015;21(12):1019-26.

16. Bernstein SL, Rosner J, DeWitt M, Tetrault J, Hsiao AL, Dziura J, et al. Design and implementation of decision support for tobacco dependence treatment in an inpatient electronic medical record: a randomized trial. 2017;7(2):185‐95.

17. Bhuiyan MM, Cohen G, Cooper S. The effect of allopurinol on pediatric patients undergoing maintenance chemotherapy for acute lymphoblastic leukemia or lymphoblastic lymphoma. Journal of Clinical and Translational Science. 2018((Cohen, Cooper) Johns Hopkins University, School of Medicine, United States):48-9.

18. Boitano L, DeVivo G, Robichaud DI, Okuhn S, Steppacher RC, Simons JP, et al. Successful Implementation of a Nurse-Navigator Run Program Utilizing Natural Language Processing Identifying patients with an abdominal aortic aneurysm. Journal of vascular surgery. 2022((Boitano, DeVivo, Robichaud, Steppacher, Simons, Aiello, Jones, Judelson, Nguyen, Sorensen, Schanzer) UMass Chan Medical School, Worcester, MA, United States(Okuhn) VA San Francisco Healthcare System; San Francisco, CA).

19. Brant AR, Kollikonda S, Yao M, Mei L, Emery J. Use of Immediate Postpartum Long-Acting Reversible Contraception Before and After a State Policy Mandated Inpatient Access. Obstetrics and gynecology. 2021;138(5):732-7.

20. Brenn BR, Choudhry DK, Sacks K. Outpatient outcomes and satisfaction in pediatric population: Data from the postoperative phone call. Paediatric Anaesthesia. 2016;26(2):158-63.

21. Brooks G, Dalby CK, Chaudary S, Jacobson JO. A pragmatic approach for measuring and monitoring hospitalizations in patients receiving chemotherapy for pancreatic cancer. American Society of Clinical Oncology; 2016.

22. Burla MJ, Shinthia N, Boura JA, Qu L, Berger DA. Resuscitation Resident Impact in the Treatment of Sepsis. Cureus. 2020;12(7):e9257.

23. Burns KA, Robbins LM, LeMarr AR, Fortune K, Morton DJ, Wilson ML. Modifiable risk factors increase length of stay and 90-day cost of care after shoulder arthroplasty. Journal of shoulder and elbow surgery. 2022;31(1):2-7.

24. Bush RA, Vemulakonda VM, Corbett ST, Chiang GJ. Can we predict a national profile of non-attendance paediatric urology patients: A multi-institutional electronic health record study. Informatics in Primary Care. 2014;21(3):132-8.

25. Bush R, Connelly C, Pérez A, Barlow H, Chiang G. Extracting autism spectrum disorder data from the electronic health record. Applied Clinical Informatics. 2017;8(3):731-41.

26. Carter ZA, Goldman S, Anderson K, Li X, Hynan LS, Chong BF, et al. Creation of an internal teledermatology store-and-forward system in an existing electronic health record: A pilot study in a safety-net public health and hospital system. JAMA Dermatology. 2017;153(7):644-50.

27. Chak EW, Luna R, MacDonald S, Stewart SL, Chen MS, Bowlus C. Automated Electronic Health Registry-Based Hepatitis B Screening. Journal of viral hepatitis. 2022((Chak, Bowlus) UC Davis School of Medicine, Division of Gastroenterology and Hepatology, Sacramento, CA, United States(Luna, MacDonald) UC Davis Medical Center, Division of Clinical Informatics, Sacramento, CA, United States(Stewart) UC Davis Department o).

28. Chang HC, Tzou DT, Usawachintachit M, Duty BD, Hsi RS, Harper JD, et al. Rationale and Design of the Registry for Stones of the Kidney and Ureter (ReSKU): A Prospective Observational Registry to Study the Natural History of Urolithiasis Patients. Journal of Endourology. 2016;30(12):1332-8.

29. Chen AM, Kupelian PA, Wang P-C, Steinberg ML. Development of a Radiation Oncology-Specific Prospective Data Registry for Research and Quality Improvement: A Clinical Workflow-Based Solution. JCO clinical cancer informatics. 2018;2(101708809):1-9.

30. Cheriff AD, Kapur AG, Qiu M, Cole CL. Physician productivity and the ambulatory EHR in a large academic multi-specialty physician group. International Journal of Medical Informatics. 2010;79(7):492-500.

31. Chernitskiy V, DeVito A, Neeman N, Sehgal N, Yazdany J, Gross AJ, editors. Integrating Collection of Rheumatoid Arthritis Disease Activity and Physical Function Scores into an Academic Rheumatology Practice to Improve Quality of Care. ARTHRITIS & RHEUMATOLOGY; 2014: WILEY-BLACKWELL 111 RIVER ST, HOBOKEN 07030-5774, NJ USA.

32. Chiu AS, Jean RA, Hoag JR, Freedman-Weiss M, Healy JM, Pei KY. Association of Lowering Default Pill Counts in Electronic Medical Record Systems with Postoperative Opioid Prescribing. JAMA Surgery. 2018;153(11):1012-9.

33. Ciricillo J, Haslam D, Blum S, Kim MO, Liu C, Paulsen G, et al. Frequency and risks associated with C lostridium difficile‐associated diarrhea after pediatric solid organ transplantation: a single‐center retrospective review. Transplant infectious disease. 2016;18(5):706-13.

34. Clendennen SL, Bowden RG, Griggs JO, Morgan GB, Umstattd Meyer MR. Risk factors associated with the timing of hospital readmission in an underserved low socioeconomic population. Hospital practice (1995). 2015;43(5):284-9.

35. Cox ML, Farjat AE, Risoli TJ, Peskoe S, Goldstein BA, Turner DA, et al. Documenting or Operating: Where Is Time Spent in General Surgery Residency? Journal of surgical education. 2018;75(6):e97-e106.

36. Dang RP, Le VH, Miles BA, Teng MS, Genden EM, Bakst RL, et al. Clinical outcomes in patients with recurrent or metastatic human papilloma virus-positive head and neck cancer. Anticancer Research. 2016;36(4):1703-9.

37. De Boer EM, Prager JD, Kerby GS, Stillwell PC. Measuring pediatric bronchoscopy outcomes using an electronic medical record. Annals of the American Thoracic Society. 2016;13(5):678-83.

38. Del Fiol G, Kohlmann W, Bradshaw RL, Weir CR, Flynn M, Hess R, et al. Standards-Based Clinical Decision Support Platform to Manage Patients Who Meet Guideline-Based Criteria for Genetic Evaluation of Familial Cancer. JCO clinical cancer informatics. 2020;4(101708809):1-9.

39. DeLozier S, Speltz P, Brito J, Tang LA, Wang J, Smith JC, et al. Real-time clinical note monitoring to detect conditions for rapid follow-up: A case study of clinical trial enrollment in drug-induced torsades de pointes and Stevens-Johnson syndrome. Journal of the American Medical Informatics Association : JAMIA. 2021;28(1):126-31.

40. Dhar JP, Essenmacher L, Dhar R, Ragina N, Sokol RJ. Lack of Uptake of Prophylactic Human Papilloma Virus Vaccine Among Women With Systemic Lupus Erythematosus Seen at a Regional Medical Center. Journal of clinical rheumatology : practical reports on rheumatic & musculoskeletal diseases. 2019;25(8):348-50.

41. Dillon EC, Erlich KJ, Li J, Li M, Becker DF. Primary care nurse practitioner management of adolescent behavioral health. The American journal of managed care. 2020;26(9):e295-e9.

42. Dinesh A, Mallick T, Arreglado TM, Altonen BL, Engdahl R. Outcomes of COVID-19 Admissions in the New York City Public Health System and Variations by Hospitals and Boroughs During the Initial Pandemic Response. Frontiers in public health. 2021;9(101616579):570147.

43. Dowling L, Zimmermann L, Shah P, Powell L, Dunham D. P3. 11-06 Improving Lung Cancer Screening Completion Rates in a Primary Care Practice in Large Urban Academic Medical Center. Journal of Thoracic Oncology. 2018;13(10):S961.

44. Egede J, Campbell JA, Walker RJ, Garacci E, Dawson AZ, Egede LE. Relationship between physical and mental health comorbidities and COVID-19 positivity, hospitalization, and mortality. Journal of affective disorders. 2021;283(h3v, 7906073):94-100.

45. Eickholtz A, Abbas S, James E, Gibson C, Iskander G, Lypka M, et al. Ride the Wave: Continuous Electroencephalography is Indicated in the Management of Traumatic Brain Injury. Clinical EEG and neuroscience. 2022;53(6):513-8.

46. Erickson M, O'Dell K, Malpartida JC, Mok J, Khan R, Patel D. Effects of Holding Beta-Blockers on the Vital Signs of Heart Failure Patients. Cardiology research. 2021;12(1):2-9.

47. Escribe C, Eisenstat SA, O'Donnell WJ, Levi R. Understanding Primary Care Physicians' Work via Text Analytics on EHR Inbox Messages. American Journal of Managed Care. 2022;28(1):E24-E30.

48. Everson J, Kocher KE, Adler-Milstein J. Health information exchange associated with improved emergency department care through faster accessing of patient information from outside organizations. Journal of the American Medical Informatics Association. 2017;24(e1):e103-e10.

49. Federman A SEBCFPJJJLMASWMKJ. Challenges optimizing the after visit summary. International Journal of Medical Informatics. 2018;120:14-9.

50. Flatow VH, Ibragimova N, Divino CM, Eshak DSA, Twohig BC, Bassily-Marcus AM, et al. Quality Outcomes in the Surgical Intensive Care Unit after Electronic Health Record Implementation. Applied Clinical Informatics. 2015;06(04):611-8.

51. Flatow V, Eshak D, Ibragimova N, Divino C, Twohig B, Marcus AB, et al. Trends in ICU Quality After Implementation of an Electronic Health Record. Chest. 2015;148(4):477A.

52. Gabel E, Hofer IS, Satou N, Grogan T, Shemin R, Mahajan A, et al. Creation and validation of an automated algorithm to determine postoperative ventilator requirements after cardiac surgery. Anesthesia and Analgesia. 2017;124(5):1423-30.

53. Gessner M, Seeland G, Gonzalez DA, Dietrich JE. Telehealth Care in a Pediatric and Adolescent Gynecology Clinic During the COVID-19 Pandemic. Journal of Pediatric and Adolescent Gynecology. 2023((Gessner, Seeland, Gonzalez, Dietrich) Department of Obstetrics & Gynecology, Baylor College of Medicine, Houston, Texas, United States(Gonzalez) Department of Pediatrics, Baylor College of Medicine, Houston, Texas, United States(Dietrich) Division of Ped).

54. Gillen JP, Riveros D, Azari L. Unusual Cause of a Cardiac Arrest in a Former African American Collegiate Athlete. Cureus. 2022;14(11):e31645.

55. Goehler A, Moore C, Manne-Goehler JM, Arango J, D'Amato L, Forman HP, et al. Clinical Decision Support for Ordering CTA-PE Studies in the Emergency Department-A Pilot on Feasibility and Clinical Impact in a Tertiary Medical Center. Academic radiology. 2019;26(8):1077-83.

56. Goldberg HS, Paterno MD, Grundmeier RW, Rocha BH, Hoffman JM, Tham E, et al. Use of a remote clinical decision support service for a multicenter trial to implement prediction rules for children with minor blunt head trauma. International Journal of Medical Informatics. 2016;87:101-10.

57. Grigoryan L, Zoorob R, Shah J, Wang H, Arya M, Trautner BW. Antibiotic prescribing for uncomplicated acute bronchitis is highest in younger adults. Antibiotics. 2017;6(4):22.

58. Handley SC, Gallagher K, Lindgren E, Lo JY, Burris HH, Dysart KC, et al. Postpartum Length of Stay and Hospital Readmission Before and During the Coronavirus Disease 2019 (COVID-19) Pandemic. Obstetrics and gynecology. 2022;139(3):381-90.

59. Hanna-Attisha M, LaChance J, Sadler RC, Schnepp AC. Elevated blood lead levels in children associated with the flint drinking water crisis: A spatial analysis of risk and public health response. American Journal of Public Health. 2016;106(2):283-90.

60. Harbison A, Gillan M. How women choose prenatal care providers in the Twin Tiers. Osteopathic Family Physician. 2018;10(6):10-5.

61. Haurani MJ, Kiser D, Vaccaro PS, Satiani B. Addition of Efficiency Measures to Current Accuracy Measures in the Vascular Laboratory Can Be Used for Future Accreditation and Payment Models. Annals of vascular surgery. 2020;65(avs, 8703941):145-51.

62. Havrilla JM, Singaravelu A, Driscoll DM, Minkovsky L, Helbig I, Medne L, et al. PheNominal: an EHR-integrated web application for structured deep phenotyping at the point of care. BMC medical informatics and decision making. 2022;22(Suppl 2):198.

63. Heidemann L, Law J, Fontana RJ. A Text Searching Tool to Identify Patients with Idiosyncratic Drug-Induced Liver Injury. Digestive Diseases and Sciences. 2017;62(3):615-25.

64. Henao D, Gregory C, Walters G, Stinson C, Dixon Y. Race and prevalence of percutaneous endoscopic gastrostomy tubes in patients with advanced dementia. Palliative & supportive care. 2022(101232529):1-6.

65. Hensley NB, Koch CG, Pronovost PJ, Mershon BH, Boyd J, Franklin S, et al. Wrong-Patient Blood Transfusion Error: Leveraging Technology to Overcome Human Error in Intraoperative Blood Component Administration. Joint Commission journal on quality and patient safety. 2019;45(3):190-8.

66. Hojat LS, Greco PJ, Bhardwaj A, Bar-Shain D, Abughali N. Using preventive health Alerts in the electronic health record improves hepatitis C virus testing among infants perinatally exposed to hepatitis C. The Pediatric Infectious Disease Journal. 2020;39(10):920-4.

67. Holmgren AJ, Rotenstein L, Downing NL, Bates DW, Schulman K. Association between state-level malpractice environment and clinician electronic health record (EHR) time. Journal of the American Medical Informatics Association : JAMIA. 2022;29(6):1069-77.

68. Horton D, Sanford M, Colledge T, Graves KK, editors. AN EHR-BASED MODIFIED EARLY WARNING SCORE PREDICTS IN-HOSPITAL MORTALITY IN PATIENTS WITH SEPSIS. JOURNAL OF GENERAL INTERNAL MEDICINE; 2018: SPRINGER 233 SPRING ST, NEW YORK, NY 10013 USA.

69. Howell L, MacDonald S, Jones J, Tancredi D, Melnikow J. Can automated alerts within computerized physician order entry improve compliance with laboratory practice guidelines for ordering Pap tests? Journal of Pathology Informatics. 2014;5(37).

70. Huang AH, Kaffenberger BH, Reich A, Szepietowski JC, Stander S, Kwatra SG. Pruritus Associated with Commonly Prescribed Medications in a Tertiary Care Center. Medicines (Basel, Switzerland). 2019;6(3).

71. Huang N, Chen L, He J, Nguyen QD. The Efficacy of Clinical Breast Exams and Breast Self-Exams in Detecting Malignancy or Positive Ultrasound Findings. Cureus. 2022;14(2):e22464.

72. Isseh M, Isseh N, Garber A, Hayat U, Burke CA, Rothberg M, et al. Sa1099 Time to Procedure: A Major Factor in Determining Inpatient Bowel Preparation Adequacy. Gastrointestinal Endoscopy. 2017;85(5):AB190.

73. Javier-DesLoges JF, Ghabili Amirkhiz K, Su JJ, Kenney PA, Advani S, Hittelman AB, et al. MP80-02 leveraging structured data in an enterprise-wide electronic health record to identify risk factors for postoperative urinary retention. The Journal of Urology. 2018;199(4S):e1089-e.

74. Javier-DesLoges JF, Johnson KK, Kenney PA, Motamedinia P. Novel Use of the Epic Electronic Medical Record Platform to Identify Lost Ureteral Stents. Journal of endourology. 2019;33(10):858-62.

75. Jones B, Scott FI, Espinoza J, Laborde S, Chambers M, Wani S, et al. Leveraging electronic medical record functionality to capture adenoma detection rate. Scientific reports. 2022;12(1):9679.

76. Jose T, Ohde JW, Taylor Hays J, Burke MV, Warner DO. Design and pilot implementation of an electronic health record-based system to automatically refer cancer patients to tobacco use treatment. International Journal of Environmental Research and Public Health. 2020;17(11):1-11.

77. Katzan IL, Fan Y, Speck M, Morton J, Fromwiller L, Urchek J, et al. Electronic stroke CarePath: integrated approach to stroke care. Circulation: Cardiovascular Quality and Outcomes. 2015;8(6_suppl_3):S179-S89.

78. Keizur E, Robinson E, Sha BE, Aziz M, Shankaran S. Effectiveness of an electronic health record model for HIV pre-exposure prophylaxis. International journal of STD & AIDS. 2022;33(5):499-502.

79. Kelly MM, Coller RJ, Hoonakker PLT, Nacht CL, Dean SM. Provider Experiences With Offering Families Bedside Health Record Access Across a Children's Hospital. Hospital pediatrics. 2020;10(11):1002-5.

80. Khanna N, Klyushnenkova EN, Kaysin A. Association of COVID-19 With Race and Socioeconomic Factors in Family Medicine. Journal of the American Board of Family Medicine : JABFM. 2021;34(Suppl):S40-S7.

81. Kim A. The Montreal Cognitive Test Intervention: The Relationship Between Cognitive-Impaired Patients and Readmissions. Professional case management. 2020;25(2):85-91.

82. King K, Galvez A, Stoltzfus J, Claros L, El Chaar M. Cost Analysis of Robotic Roux-en-Y Gastric Bypass in a Single Academic Center: How Expensive Is Expensive? Obesity surgery. 2020;30(12):4860-6.

83. Klang E, Levin MA, Soffer S, Zebrowski A, Glicksberg BS, Carr BG, et al. A simple free-text-like method for extracting semi-structured data from electronic health records: Exemplified in prediction of in-hospital mortality. Big Data and Cognitive Computing. 2021;5(3).

84. Kolb EA, Coyne E, Crowgey E, Raymond V, Wadman J, Jacobs-Allen S, et al. Improving Care of Patients with Sickle Cell Disease through Discrete Clinical Data Extraction and Automated Analysis of the Electronic Healthcare Records. Blood. 2016;128(22):2331.

85. Koo JK, Moyer L, Castello MA, Arain Y. Improving Accuracy of Handoff by Implementing an Electronic Health Record-generated Tool: An Improvement Project in an Academic Neonatal Intensive Care Unit. Pediatric quality & safety. 2020;5(4):e329.

86. Kukhareva PV, Caverly TJ, Li H, Katki HA, Cheung LC, Reese TJ, et al. Inaccuracies in electronic health records smoking data and a potential approach to address resulting underestimation in determining lung cancer screening eligibility. Journal of the American Medical Informatics Association : JAMIA. 2022;29(5):779-88.

87. Kurian AW, Mitani A, Desai M, Yu PP, Seto T, Weber SC, et al. Breast cancer treatment across health care systems: Linking electronic medical records and state registry data to enable outcomes research. Cancer. 2014;120(1):103-11.

88. Kurin M, Elangovan A, Alikhan MM, Al Dulaijan B, Silver E, Kaelber DC, et al. Irritable bowel syndrome is strongly associated with the primary and idiopathic mast cell disorders. Neurogastroenterology and motility : the official journal of the European Gastrointestinal Motility Society. 2022;34(5):e14265.

89. Kuznetsov JL, Bailey K, Bombach PK, Carmichael S, Chen X, Herberg ML, et al. Real-time extraction of breast cancer treatment process and outcome measures from an EPIC electronic health record (EHR). American Society of Clinical Oncology; 2013.

90. LaHue SC, Escueta DP, Guterman EL, Patel K, Harrison KL, Boscardin WJ, et al. COVID-19 severity and age increase the odds of delirium in hospitalized adults with confirmed SARS-CoV-2 infection: a cohort study. BMC psychiatry. 2022;22(1):151.

91. Lanzo E, Taeger A, Huston-Paterson H, Tomaszewski K, Trent M. Increasing Patient Portal Usage: Outcomes From the MyChart Genius Pilot Project. Journal of Adolescent Health. 2016;58(2):S18.

92. Li G, Dietz CJK, Freundlich RE, Shotwell MS, Wanderer JP. The Impact of an Intraoperative Clinical Decision Support Tool to Optimize Perioperative Glycemic Management. Journal of Medical Systems. 2020;44(10).

93. Lilih S, Pereboom M, van der Hoeven RTM, Mantel-Teeuwisse AK, Becker ML. Improving the effectiveness of drug safety alerts to increase adherence to the guideline for gastrointestinal prophylaxis. International Journal of Medical Informatics. 2017;97((Lilih, Pereboom, van der Hoeven, Becker) Pharmacy Foundation of Haarlem Hospitals, Boerhaavelaan 24, Haarlem 2035 RC, Netherlands(Lilih, Mantel-Teeuwisse) Division of Pharmacoepidemiology & Clinical Pharmacology, Utrecht Institute for Pharmaceutical Scie):139-44.

94. Lindholm C, Adsit R, Bain P, Reber PM, Brein T, Redmond L, et al. A demonstration project for using the electronic health record to identify and treat tobacco users. WMJ: official publication of the State Medical Society of Wisconsin. 2010;109(6):335.

95. Loo NM-M, Taddei TH, editors. Using a European Value-Based Medicine Approach to Evaluate Hepatocellular Carcinoma Care at a US Tertiary Care Center2015: American Society of Clinical Oncology.

96. Mahajan AK, Collar N, Bari M, Nader A, Muldowney F, Patel PP, et al. Effectiveness of an Electronic Medical Record-Based Recognition Tool for the Identification of Incidental Pulmonary Nodules. Journal of bronchology & interventional pulmonology. 2022(101496866).

97. Makam AN, Lanham HJ, Batchelor K, Samal L, Moran B, Howell-Stampley T, et al. Use and satisfaction with key functions of a common commercial electronic health record: a survey of primary care providers. BMC medical informatics and decision making. 2013;13(1):1-7.

98. Manzar S. Social Determinants of Health Among Pregnant Women. Neonatology Today. 2022;17(2):3-10.

99. Marsolo K, Corsmo J, Barnes MG, Pollick C, Chalfin J, Nix J, et al. Challenges in creating an opt-in biobank with a registrar-based consent process and a commercial EHR. Journal of the American Medical Informatics Association. 2012;19(6):1115-8.

100. Martin JR, Filip P, Thorpe EJ, Leonetti JP. Treatment of locally advanced parotid malignancies with parotidectomy and temporal bone resection. American Journal of Otolaryngology - Head and Neck Medicine and Surgery. 2017;38(4):380-2.

101. Mathias J, Gossett D, Thompson J, Baker D, editors. USE OF ELECTRONIC HEALTH RECORD DATA TO ASSESS PAP SMEAR OVERUSE IN A GENERAL INTERNAL MEDICINE CLINIC. Journal of General Internal Medicine; 2010: SPRINGER 233 SPRING ST, NEW YORK, NY 10013 USA.

102. McCain JL, Wang X, Connell K, Morgan J. Assessing the impact of insurance type on COVID-19 mortality in black and white patients in the largest healthcare system in the state of georgia. Journal of the National Medical Association. 2022;114(2):218-26.

103. McCarthy MM, Fletcher J, Heffron S, Szerencsy A, Mann D, Vorderstrasse A. Implementing the physical activity vital sign in an academic preventive cardiology clinic. Preventive medicine reports. 2021;23(101643766):101435.

104. McDowell J, Wu A, Ehrenfeld JM, Urman RD. Effect of the Implementation of a New Electronic Health Record System on Surgical Case Turnover Time. Journal of Medical Systems. 2017;41(3).

105. Mehta R, Radhakrishnan NS, Warring CD, Jain A, Fuentes J, Dolganiuc A, et al. The use of evidence-based, problem-oriented templates as a clinical decision support in an inpatient electronic health record system. Applied clinical informatics. 2016;7(03):790-802.

106. Mehta PH, Chhablani J, Wang J, Meyerle CB. Central Serous Chorioretinopathy in African Americans at Wilmer Eye Institute. Journal of the National Medical Association. 2018;110(3):297-302.

107. Melnick ER, Nath B, Dziura JD, Casey MF, Jeffery MM, Paek H, et al. User centered clinical decision support to implement initiation of buprenorphine for opioid use disorder in the emergency department: EMBED pragmatic cluster randomized controlled trial. BMJ (Clinical research ed). 2022;377(8900488, bmj, 101090866):e069271.

108. Milani RV, Lavie CJ, Bober RM, Milani AR, Ventura HO. Improving Hypertension Control and Patient Engagement Using Digital Tools. American Journal of Medicine. 2017;130(1):14-20.

109. Milne M, Roehrborn C, Gruntmanis U. Androgen Deprivation Therapy for Prostate Cancer: Specialty Clinic Collaboration and the Electronic Medical Record Can Improve Bone Health Monitoring. Urology Practice. 2020;7(4):294-8.

110. Mirro MJ, Keltner EE, Roebuck AE, Sears SF. Playing it close to the VEST and the clinical guidelines: Clinical guideline compliance in HFrEF patients-Role of WCD. Pacing and clinical electrophysiology : PACE. 2018;41(10):1314-20.

111. Mitchell JD, Lenihan DJ, Reed C, Huda A, Nolen K, Bruno M, et al. Implementing a Machine-Learning-Adapted Algorithm to Identify Possible Transthyretin Amyloid Cardiomyopathy at an Academic Medical Center. Clinical Medicine Insights Cardiology. 2022;16(101525768):11795468221133608.

112. Mosk CA, Mus M, Vroemen JPAM, Van Der Ploeg T, Vos DI, Elmans LHGJ, et al. Dementia and delirium, the outcomes in elderly hip fracture patients. Clinical Interventions in Aging. 2017;12((Mosk, Mus, Vroemen, Vos, Van Der Laan) Department of Surgery, Amphia Hospital, Breda, Netherlands(Van Der Ploeg) Department of Public Health, Erasmus MC-University Medical Center, Rotterdam, Netherlands(Elmans) Department of Orthopedic Surgery, Amphia Ho):421-30.

113. Mou Z, Sitapati AM, Ramachandran M, Doucet JJ, Liepert AE. Development and implementation of an automated electronic health record-linked registry for emergency general surgery. The journal of trauma and acute care surgery. 2022;93(2):273-9.

114. Mulhem E, Brown I, Song K. Electronic Health Record Reminder Effect on Hepatitis C Antibody Screening. Journal of the American Board of Family Medicine : JABFM. 2020;33(6):1016-9.

115. Munoz R, Farshidpour L, Chaudhary UB, Fathi AH. Multidisciplinary Cancer Care Model: A Positive Association Between Oncology Nurse Navigation and Improved Outcomes for Patients With Cancer. Clinical journal of oncology nursing. 2018;22(5):E141-E5.

116. Muqri H, Shrivastava A, Muhtadi R, Chuck RS, Mian UK. The Cost-Effectiveness of a Telemedicine Screening Program for Diabetic Retinopathy in New York City. Clinical ophthalmology (Auckland, NZ). 2022;16(101321512):1505-12.

117. Nagi T, Somvanshi S, Balasubramania Pandian GSD, Mohan S, Altonen B. An Audit of Inpatient Consultation-Liaison Psychiatry Services at an Inner New York City Safety Net Hospital. Cureus. 2023;15(2):e34801.

118. Naranjo J, Portner ER, Jakub JW, Cheville AL, Nuttall GA. Ipsilateral Intravenous Catheter Placement in Breast Cancer Surgery Patients. Anesthesia and analgesia. 2021;133(3):707-12.

119. Narayanan J, Simon KC, Choi J, Dobrin S, Rubin S, Taber J, et al. Factors Affecting Cognition and Depression in Adult Patients with Epilepsy. Journal of epilepsy research. 2019;9(2):103-10.

120. Nasehi L, Sturgis CD, Sharma N, Turk P, Calhoun BC. Breast Cancer Risk Associated With Benign Intraductal Papillomas Initially Diagnosed on Core Needle Biopsy. Clinical breast cancer. 2018;18(6):468-73.

121. Nellis JR, Prabhu NK, Hoover AC, Muller MJ, Overbey DM, Chen EP, et al. Understanding and Managing Direct Operating Room Supply Costs in Cardiac Surgery. The Annals of thoracic surgery. 2022(15030100R).

122. Nguyen ML, Fischer G. Re-framing hepatitis c decision-support alerts: a pilot project in human-centered design. 2020;35(SUPPL 1):S254.

123. Ni J, Friedman H, Boyd BC, McGurn A, Babinski P, Markossian T, et al. Early antibiotic exposure and development of asthma and allergic rhinitis in childhood. BMC pediatrics. 2019;19(1):225.

124. Nikolian VC, Williams AM, Jacobs BN, Kemp MT, Wilson JK, Mulholland MW, et al. Pilot Study to Evaluate the Safety, Feasibility, and Financial Implications of a Postoperative Telemedicine Program. Annals of surgery. 2018;268(4):700-7.

125. Nikolic D, Richter SS, Asamoto K, Wyllie R, Tuttle R, Procop GW. Implementation of a clinical decision support tool for stool cultures and parasitological studies in hospitalized patients. Journal of Clinical Microbiology. 2017;55(12):3350-4.

126. Nolan MS, Lynn MK, Lacroix R, Brownlee J, Kelly D. Adolescent Trichomonas vaginalis in a High-burdened Region of the Southern United States. Sexually transmitted diseases. 2020;47(7):499-502.

127. Norton L, Tsiperfal A, Cook K, Bagdasarian A, Varady J, Shah M, et al. Effectiveness and Safety of an Independently Run Nurse Practitioner Outpatient Cardioversion Program (2009 to 2014). American Journal of Cardiology. 2016;118(12):1842-6.

128. Noshad M, Rose CC, Chen JH. Signal from the noise: A mixed graphical and quantitative process mining approach to evaluate care pathways applied to emergency stroke care. Journal of biomedical informatics. 2022;127(100970413, d2m):104004.

129. Osband A, Machan J, Morrissey P, editors. Benefit of Patient Portal Usage in Non-High Risk Kidney Transplant Recipients. AMERICAN JOURNAL OF TRANSPLANTATION; 2018: WILEY 111 RIVER ST, HOBOKEN 07030-5774, NJ USA.

130. Osterberg EC, Palmer NRA, Harris CR, Murphy GP, Blaschko SD, Chu C, et al. Outcomes of men on active surveillance for low-risk prostate cancer at a safety-net hospital. Urologic Oncology: Seminars and Original Investigations. 2017;35(11):e9-663.

131. Otto W. #41 Manual Validation of an Automated Tool to Extract Blood Culture and Susceptibility Data from the Electronic Health Record for Children with Acute Myeloid Leukemia...St. Jude/Pediatric Infectious Diseases Society (PIDS) Conference, virtual, March 3-5. Journal of the Pediatric Infectious Diseases Society. 2022;11:S2-S.

132. Palestine AG, Merrill PT, Saleem SM, Jabs DA, Thorne JE. Assessing the Precision of ICD-10 Codes for Uveitis in 2 Electronic Health Record Systems. JAMA Ophthalmology. 2018;136(10):1186-90.

133. Park JS, Wong J, Cohen H. Hepatitis C virus screening of high-risk patients in a community hospital emergency department: Retrospective review of patient characteristics and future implications. PloS one. 2021;16(6):e0252976.

134. Park KE, Ramachandran V, Tran J, Joshi TP, Garg N, Duvic M. Association of Flame-Retardant Clothing With Mycosis Fungoides: A Retrospective Analysis. Dermatology practical & conceptual. 2022;12(2):e2022091.

135. Patil SA, Dygert L, Galetta SL, Balcer LJ, Cohen EJ. Apparent lack of association of COVID-19 vaccination with Herpes Zoster. American journal of ophthalmology case reports. 2022;26(101679941):101549.

136. Pho KK, Lu R, Gates S, Cai J, Xie D, Xie Y, et al. Mobile Device Applications for Electronic Patient Portals in Oncology. JCO clinical cancer informatics. 2019;3(101708809):1-8.

137. Putka B, Mullen K, Birdi S, Merheb M. The disposition of hepatitis C antibody-positive patients in an urban hospital. Journal of Viral Hepatitis. 2009;16(11):814-21.

138. Raja A, Trivedi PD, Nabeel I. Development of a return to work tool for primary care providers for low back pain patients: pilot study assessment. 2017;9(9):S283.

139. Rajamani S, Bieringer A, Muscoplat M. Characterizing the Access of Clinical Decision Support Offered by Immunization Information System in Minnesota. Online Journal of Public Health Informatics. 2016;7(3).

140. Ram DKN, Gulzar K, Alalawi F, Ahmed M, Manuel R, Alhadari A. COVID-19 Infection in Hemodialysis Patients, Incidence, Risk Factors and Mortality. Single Centre Study. New Emirates Medical Journal. 2022;3(2).

141. Rameau A, Wang E, Saraswathula A, Pageler N, Perales S, Sidell DR. Enhancing pediatric airway safety using the electronic medical record. The Laryngoscope. 2018;128(12):2885-92.

142. Ray S, McEvoy DS, Aaron S, Hickman TT, Wright A. Using statistical anomaly detection models to find clinical decision support malfunctions. Journal of the American Medical Informatics Association. 2018;25(7):862-71.

143. Read-Brown S, Hribar MR, Reznick LG, Lombardi LH, Parikh M, Chamberlain WD, et al. Time requirements for electronic health record use in an academic ophthalmology center. Jama Ophthalmology. 2017;135(11):1250-7.

144. Redd TK, Read-Brown S, Choi D, Yackel TR, Tu DC, Chiang MF. Electronic health record impact on productivity and efficiency in an academic pediatric ophthalmology practice. Journal of American Association for Pediatric Ophthalmology and Strabismus. 2014;18(6):584-9.

145. Reddy SR, Mouchli M, Summey R, Walsh C, Mir A, Bierle L, et al. Outcomes of Young Patients With Alcoholic Cirrhosis After First Hospitalization for Cirrhosis: A Carilion Clinic Experience. Cureus. 2021;13(7):e16695.

146. Renjithlal SLM, Magdi M, Mostafa MR, Renjith K, Pillai P, Syed M, et al. Bone Mineral Density as a Predictor of Cardiovascular Disease in Women: A Real-World Retrospective Study. Journal of Endocrinology and Metabolism. 2022;12(4-5):125-33.

147. Ritchey P, Graham B, Phelan M, Hustey F, Chamberlin J, Engineer R. 177 Can an Electronic Health Record Order Set Improve Analgesia and Sedation in the Intubated Emergency Department Patient? Annals of Emergency Medicine. 2016;68(4):S70.

148. Rizk S, Axelrod D, Riddick-Burden G, Congdon-Martin E, McKenzie S, Haines C, et al. Clinical Transformation in Care for Patients With Sickle Cell Disease at an Urban Academic Medical Center. American journal of medical quality : the official journal of the American College of Medical Quality. 2020;35(3):236-41.

149. Rodriguez RR, Babar L, Lo H, Ashraf O, Monga D, Finley G, et al. A Retrospective Analysis of Factors Affecting Palliative Care Consults in Patients Undergoing Cytoreductive Surgery and Hyperthermic Intraperitoneal Chemotherapy. Cureus. 2021;13(1):e12589.

150. Rollman BL, Belnap BH, Rothenberger SD, Abebe K, Rotondi AJ, Karp JF. Online treatments for mood and anxiety disorders in primary care: a randomized controlled trial. 2016;31(2):S316‐S7.

151. Rose AC, Costea A, Jandarov R, Eckman MH, editors. POPULATION-BASED ANALYSIS OF AN EHR-EMBEDDED DECISION SUPPORT TOOL FOR LEFT ATRIAL APPENDAGE CLOSURE DEVICE IN PATIENTS WITH ATRIAL FIBRILLATION. JOURNAL OF GENERAL INTERNAL MEDICINE; 2018: SPRINGER 233 SPRING ST, NEW YORK, NY 10013 USA.

152. Rose C, Thombley R, Noshad M, Lu Y, Clancy HA, Schlessinger D, et al. Team is brain: leveraging EHR audit log data for new insights into acute care processes. Journal of the American Medical Informatics Association : JAMIA. 2022;30(1):8-15.

153. Rove KO, Warncke JC, Vemulakonda VM. Trends in pediatric urologic consultations in a tertiary care hospital setting. Journal of pediatric urology. 2018;14(1):12.e1-.e8.

154. Ruan E, Beiser M, Lu V, Paul S, Ni J, Nazar N, et al. Physician Electronic Health Record Usage as Affected by the COVID-19 Pandemic. Applied clinical informatics. 2022;13(4):785-93.

155. Ryan P, Furniss A, Breslin K, Everhart R, Hanratty R, Rice J. Assessing and Augmenting Predictive Models for Hospital Readmissions With Novel Variables in an Urban Safety-net Population. Medical care. 2021;59(12):1107-14.

156. Sahu N, Chen P-H, Shimoni Na. Telehealth to improve continuity for patients receiving buprenorphine treatment for opioid use disorder. Annals of family medicine. 2022;20(20 Suppl 1).

157. Salem JF, Bauerle WB, Arishi AA, Stoltzfus J, El Chaar M. Direct medical costs of robotic sleeve gastrectomy compared to laparoscopic approach in a single academic center. Journal of robotic surgery. 2023;17(1):49-54.

158. Sawalha K, Habash FJ, Vallurupalli S, Paydak H. Theophylline in Treatment of COVID-19 Induced Sinus Bradycardia. Clinics and practice. 2021;11(2):332-6.

159. Schleelein LE, Vincent AM, Jawad AF, Pruitt EY, Kreher GD, Rehman MA, et al. Pediatric perioperative adverse events requiring rapid response: A retrospective case-control study. Paediatric Anaesthesia. 2016((Schleelein, Vincent, Jawad, Pruitt, Kreher, Rehman, Goebel, Cohen, Cook-Sather) Department of Anesthesiology and Critical Care Medicine The Children's Hospital of Philadelphia and the Perelman School of Medicine at the University of Pennsylvania Philadel).

160. Serna MK, Fiskio J, Yoon C, Plombon S, Lakin JR, Schnipper JL, et al. Who Gets (and Who Should Get) a Serious Illness Conversation in the Hospital? An Analysis of Readmission Risk Score in an Electronic Health Record. American Journal of Hospice and Palliative Medicine. 2022.

161. Shelden D, Ateya M, Jensen A, Arnold P, Bellomo T, Gianchandani R. Improving Hospital Glucometrics, Workflow, and Outcomes with a Computerized Intravenous Insulin Dose Calculator Built into the Electronic Health Record. Journal of diabetes science and technology. 2021;15(2):271-8.

162. Shelley D, Goldfeld KS, Park H, Mola A, Sullivan R, Austrian J. System Changes to Implement the Joint Commission Tobacco Treatment (TOB) Performance Measures for Improving the Treatment of Tobacco Use Among Hospitalized Patients. Joint Commission Journal on Quality and Patient Safety. 2017.

163. Siegfried E, Darji K. Interferon-gamma treatment for children with recurrent eczema herpeticum: a retrospective review. 2015;135:S28.

164. Siff JE, Emerman CL. A computer-assisted process to reduce discharge of emergency department patients with abnormal vital signs. Journal of Clinical Outcomes Management. 2016;23(11):505-8.

165. Simmons JK, Leiman DA, Patil SU, McCoul E, Chen PG, Tang DM, et al. Increased Prevalence of Eosinophilic Esophagitis in Patients With Chronic Rhinosinusitis. American journal of rhinology & allergy. 2022;36(6):804-7.

166. Simon EL, Crouse B, Wilson M, Muir M, Sayles S, Ramos C, et al. Evaluation of missed influenza vaccination opportunities in the emergency department. The American journal of emergency medicine. 2023;68(aa2, 8309942):59-63.

167. Simon AR, Ahmed KL, Limon DL, Duhon GF, Marzano G, Goin-Kochel RP. Utilization of a Best Practice Alert (BPA) at Point-of-Care for Recruitment into a US-Based Autism Research Study. Journal of autism and developmental disorders. 2023;53(1):359-69.

168. Snyder SD, Williams A, Mitchell M, Kneebusch J. Use of Intramuscular Chlorpromazine Versus Intramuscular Olanzapine for the Management of Acute Agitation and Aggression in Youth. The journal of pediatric pharmacology and therapeutics : JPPT : the official journal of PPAG. 2021;26(1):33-41.

169. Sonstein L, Clark C, Seidensticker S, Zeng L, Sharma G. Improving adherence for management of acute exacerbation of chronic obstructive pulmonary disease. American Journal of Medicine. 2014;127(11):1097-104.

170. Sroujieh LS, Monroy D, Warren E. Using electronic health record (EHR) best practice alert (BPA) to improve RBC transfusion practices and adherence to the guidelines. Chest. 2016;150(4):599A.

171. Stapel JR, Speed JS, Clemmer JS. Endothelin antagonism reduces hemoglobin A1c in patients with pulmonary hypertension. Canadian journal of physiology and pharmacology. 2022;100(8):828-33.

172. Straub H, Adams M, Ng D, Silver R, editors. Can an electronic health record system be used to provide population-based preconception care? American Journal of Obstretrics and Gynecology Conference: 33rd Annual Meeting of the Society for Maternal-Fetal Medicine: The Pregnancy Meeting; 2013.

173. Stutz M, Phung P, Vang J, Jain A, Ross M. Clinical Decision Support to Improve of End-of-Life Planning at Inpatient Discharge. C38 IMPROVING QUALITY AND UTILIZATION OF END OF LIFE AND PALLIATIVE CARE: American Thoracic Society; 2018. p. A4874-A.

174. Su J, Javier-DesLoges J, Leapman M. MP51-09 EXAMINING POST-OPERATIVE OPIOID PRESCRIBING PATTERNS FOLLOWING UROLOGIC SURGERY USING AN ENTERPRISE-WIDE ELECTRONIC MEDICAL RECORD. The Journal of Urology. 2018;199(4S):e688-e.

175. Ter-Minassian M, Lanzkron S, Derus A, Brown E, Horberg MA. Quality Metrics and Health Care Utilization for Adult Patients with Sickle Cell Disease. Journal of the National Medical Association. 2019;111(1):54-61.

176. Tham E, Swietlik M, Deakyne S, Hoffman JM, Grundmeier RW, Paterno MD, et al. Clinical decision support for a multicenter trial of pediatric head trauma. Applied clinical informatics. 2016;7(02):534-42.

177. Tham E, Swietlik M, Deakyne S, Hoffman JM, Grundmeier RW, Paterno MD, et al. Clinical Decision Support for a Multicenter Trial of Pediatric Head Trauma: Development, Implementation, and Lessons Learned. Appl Clin Inform. 2016;7(2):534-42.

178. Torres CIH, Gold R, Kaufmann J, Marino M, Hoopes MJ, Totman MS, et al. Social Risk Screening and Response Equity: Assessment by Race, Ethnicity, and Language in Community Health Centers. American journal of preventive medicine. 2023(8704773, apl).

179. Toscos T, Daley C, Wagner S, Coupe A, Ahmed R, Holden RJ, et al. Patient responses to daily cardiac resynchronization therapy device data: A pilot trial assessing a novel patient-centered digital dashboard in everyday life. Cardiovascular digital health journal. 2020;1(2):97-106.

180. Toscos T, Coupe A, Wagner S, Ahmed R, Roebuck A, Flanagan M, et al. Engaging Patients in Atrial Fibrillation Management via Digital Health Technology: The Impact of Tailored Messaging. The Journal of innovations in cardiac rhythm management. 2020;11(8):4209-17.

181. Tsai C, Patel K, Vincent A, Verzosa N, Norris D, Tillis W, et al. 253 Electronic Best Practice Advisories' Effectiveness in Detecting Sepsis in the Emergency Department. Annals of Emergency Medicine. 2015;66(4):S91-S2.

182. Unni S, Yao Y, Milne N, Gunning K, Curtis JR, LaFleur J. An evaluation of clinical risk factors for estimating fracture risk in postmenopausal osteoporosis using an electronic medical record database. Osteoporosis International. 2015;26(2):581-7.

183. van den Broek AK, Beishuizen BHH, Haak EAF, Duyvendak M, Ten Oever J, Sytsma C, et al. A mandatory indication-registration tool in hospital electronic medical records enabling systematic evaluation and benchmarking of the quality of antimicrobial use: a feasibility study. Antimicrobial resistance and infection control. 2021;10(1):103.

184. Vemulakonda V, Janzen N, Sevick C, Chiang G. Variations in adherence to AUA guidelines for vesicoureteral reflux. 2017;197(4):e805.

185. Vemulakonda VM, Janzen N, Hittelman AB, Deakyne Davies S, Sevick C, Richardson AC, et al. Feasibility of establishing a multi-center research database using the electronic health record: The PURSUIT network. Journal of pediatric urology. 2022;18(6):788.e1-.e8.

186. Vesco K, Beadle K, Bulkley J, Stoneburner A, Leo M, Clark A. Clinician education and electronic medical record tools to enhance care for genitourinary syndrome of menopause-a randomized trial. 2016;23(12):1377‐8.

187. Vlashyn OO, Murphy CV, Cape K, Phelps MK, Nunez SM, Hafford A, et al. Evaluation of an electronic health record documentation tool use to improve pharmacist intervention tracking in the intensive care unit. JACCP Journal of the American College of Clinical Pharmacy. 2023((Vlashyn, Murphy, Cape, Phelps, Hafford, Jordan, Smetana) Department of Pharmacy, The Ohio State University Wexner Medical Center, Columbus, OH, United States(Nunez) The Ohio State University College of Pharmacy, Columbus, OH, United States).

188. Wagner LI, Ph D, Schink J, Bass M, Patel S, Diaz MV, et al. Bringing PROMIS to practice: Brief and precise symptom screening in ambulatory cancer care. Cancer. 2016;121(6):927-34.

189. Wagner K, Pingle S-R, Walker K, Floridia E, Medina C, Rowe CK. Harnessing the epic electronic medical record to track indwelling ureteral stents in a pediatric population. Journal of pediatric urology. 2022;18(1):23.e1-.e5.

190. Walter SM, Dai Z, Wang K. Obesity, Migraine, and Overlapping Comorbidities in a Rural Pediatric Population. Journal of neurosciences in rural practice. 2021;12(3):524-9.

191. Wamsley MA, Steiger S, Julian KA, Gleason N, O'Sullivan PS, Guy M, et al. Teaching residents screening, brief intervention, and referral to treatment (SBIRT) skills for alcohol use: using chart-stimulated recall to assess curricular impact. Substance abuse. 2016;37(3):419-26.

192. Wang JK, Ouyang D, Hom J, Chi J, Chen JH. Characterizing electronic health record usage patterns of inpatient medicine residents using event log data. PloS one. 2019;14(2):e0205379.

193. Wendel SK, Bookman K, Holmes M, Wiler JL. Successful Implementation of Workflow-Embedded Clinical Pathways During the COVID 19 Pandemic. Quality management in health care. 2023(bv0, 9306156).

194. Weng J, Mesko S, Das P, Koong AC, Herman JM, Elrod-Joplin D, et al. Optimizing Radiation Oncology Consult Visits Using Patient Flow Analysis (PFA). International Journal of Radiation Oncology, Biology, Physics. 2021;111(3):S93-S.

195. Williams MC, Obermeier H, Hurst AL, Saporta-Keating SR, Pearce K, MacBrayne CE, et al. Hospital-wide Description of Clinical Indications for Pediatric Anti-infective Use. Clinical therapeutics. 2019;41(8):1605-11.e0.

196. Willis C, Watanabe AH, Hughes J, Nolen K, O'Meara J, Schepart A, et al. Applying diagnosis support systems in electronic health records to identify wild-type transthyretin amyloid cardiomyopathy risk. Future cardiology. 2022;18(5):367-76.

197. Winden TJ, Boland LL, Frey NG, Satterlee PA, Hokanson JS. Care everywhere, a point-to-point HIE tool: utilization and impact on patient care in the ED. Applied clinical informatics. 2014;5(2):388-401.

198. Wu C, Shah N, Sood P, Puttarajappa C, Bernardo J, Mehta R, et al. Use of the Electronic Health Record (EHR) to Improve the Pre-Transpalnt Process for Kidney and Pancreas Transplantation.: Abstract# B1219. Transplantation. 2014;98:833-4.

199. Wu A, Mihaylova VT, Landry ML, Foxman EF. Interference between rhinovirus and influenza A virus: a clinical data analysis and experimental infection study. The Lancet Microbe. 2020;1(6):e254-e62.

200. Wyatt B, Perumalswami PV, Mageras A, Miller M, Harty A, Ma N, et al. A Digital Case-Finding Algorithm for Diagnosed but Untreated Hepatitis C: A Tool for Increasing Linkage to Treatment and Cure. Hepatology (Baltimore, Md). 2021;74(6):2974-87.

201. Yazdanshenas H, Osias E, Hwang R, Park DY, Lord E, Shamie AN. Retrospective evaluation of cervical fusion with DTRAX (R) cervical cage. Journal of craniovertebral junction & spine. 2022;13(1):48-54.

202. Zazove P, McKee M, Schleicher L, Green L, Kileny P, Rapai M, et al. To act or not to act: Responses to electronic health record prompts by family medicine clinicians. Journal of the American Medical Informatics Association. 2017;24(2):275-80.

203. Zetumer S, Tzou D, Ahmad T, Reliford-Titus S, Stoller M, Chi T. MP50-15 AUTOMATED DATA EXTRACTION FROM AN INTEGRATED ELECTRONIC HEALTH RECORDS DATA REGISTRY YIELDS HIGHER ACCURACY THAN MANUAL ENTRY: RESULTS FROM THE REGISTRY FOR STONES OF THE KIDNEY AND URETER (RESKU). The Journal of Urology. 2018;199(4S):e681-e.

204. Zhang CQ, Gogal C, Gaugler T, Blome-Eberwein S. A 6-Year Experience of Laser Treatments for Burn Scars in a Regional Burn Center-Safety, Efficacy, and Quality Improvement. Journal of burn care & research : official publication of the American Burn Association. 2021;42(1):74-81.

205. Zhou Y, Hou Y, Hussain M, Brown S-A, Budd T, Tang WHW, et al. Machine Learning-Based Risk Assessment for Cancer Therapy-Related Cardiac Dysfunction in 4300 Longitudinal Oncology Patients. Journal of the American Heart Association. 2020;9(23):e019628.

206. Zorn KK, Simonson ME, Faulkner JL, Carr CL, Acuna J, Hall TL, et al. Can Automated Alerts in the Electronic Health Record Encourage Referrals for Genetic Counseling and Testing Among Patients at High Risk for Hereditary Cancer Syndromes? JCO oncology practice. 2022;18(7):e1219-e24.
